# Biogenic nanoparticles: pioneering a new era in breast cancer therapeutics—a comprehensive review

**DOI:** 10.1186/s11671-024-04072-y

**Published:** 2024-08-03

**Authors:** Shahnawaz Ahmad Bhat, Vijay Kumar, Daljeet Singh Dhanjal, Yashika Gandhi, Sujeet K. Mishra, Simranjeet Singh, Thomas J. Webster, Praveen C. Ramamurthy

**Affiliations:** 1 Jamia Milia Islamia, New Delhi, 110011 India; 2 Central Ayurveda Research Institute, Jhansi, U.P. 284003 India; 3 Lovely Professional University, Phagwara, Punjab 144111 India; 4 Indian Institute of Sciences, Bangalore, 560012 India; 5 School of Health Sciences and Biomedical Engineering, Hebei University of Technology, Tianjin, China; 6 Program in Materials Science, UFPI, Teresina, Brazil

**Keywords:** Breast cancer, Biogenic nanoparticles, Conventional treatment, Nanomaterials, Medicinal plants

## Abstract

Breast cancer, a widespread malignancy affecting women globally, often arises from mutations in estrogen/progesterone receptors. Conventional treatments like surgery, radiotherapy, and chemotherapy face limitations such as low efficacy and adverse effects. However, nanotechnology offers promise with its unique attributes like targeted delivery and controlled drug release. Yet, challenges like poor size distribution and environmental concerns exist. Biogenic nanotechnology, using natural materials or living cells, is gaining traction for its safety and efficacy in cancer treatment. Biogenic nanoparticles synthesized from plant extracts offer a sustainable and eco-friendly approach, demonstrating significant toxicity against breast cancer cells while sparing healthy ones. They surpass traditional drugs, providing benefits like biocompatibility and targeted delivery. Thus, this current review summarizes the available knowledge on breast cancer (its types, stages, histopathology, symptoms, etiology and epidemiology) with the importance of using biogenic nanomaterials as a new and improved therapy. The novelty of this work lies in its comprehensive examination of the challenges and strategies for advancing the industrial utilization of biogenic metal and metal oxide NPs. Additionally; it underscores the potential of plant-mediated synthesis of biogenic NPs as effective therapies for breast cancer, detailing their mechanisms of action, advantages, and areas for further research.

## Introduction

Cancer, a multifaceted and intricate disease, manifests and is characterized by the uncontrolled proliferation and dissemination of abnormal cells in various forms, thereby impacting diverse organs within the body [1]. Even the human defense system fails to identify them as they seem identical to normal cells. Thus, tumors can be classified as either benign, remaining localized and non-cancerous, or malignant, possessing the ability to metastasize to distant sites via the bloodstream or lymphatic system [2]. The cancer process is associated with an extensive set of diverse up-regulation and down-regulation of cellular genes and proteins, such as those that regulate normal cell development, cell adhesion receptors allied to cell mobility, and genes associated with tumor dominance [3]. Additionally, cancer is a major contributor in global mortality rate, posing a significant barrier to increasing life-expectancy globally. As per the consensus of the World Health Organization (WHO), cancer holds the second position as a major cause of death before the age of 70 in 112 out of 183 countries. In 2020, there were approximately 19.3 million new cases (excluding non-melanoma skin cancer), resulting in 10.8 million cancer-associated deaths. It is predicted that cancer incidences will substantially increase by 47% to reach 28.4 million cases in 2040, with a more substantial increase expected in transitioning countries due to demographic changes and risks associated with globalization and economic growth [4]. Even though the United States and Europe are the most technologically advanced regions in the world [5], they are still combating intensifying cancer cases. This subsequent increase in cancer cases has been found to be associated with various factors like alcohol consumption, high-caloric intake and tobacco consumption [6].

**Fig. 1:**
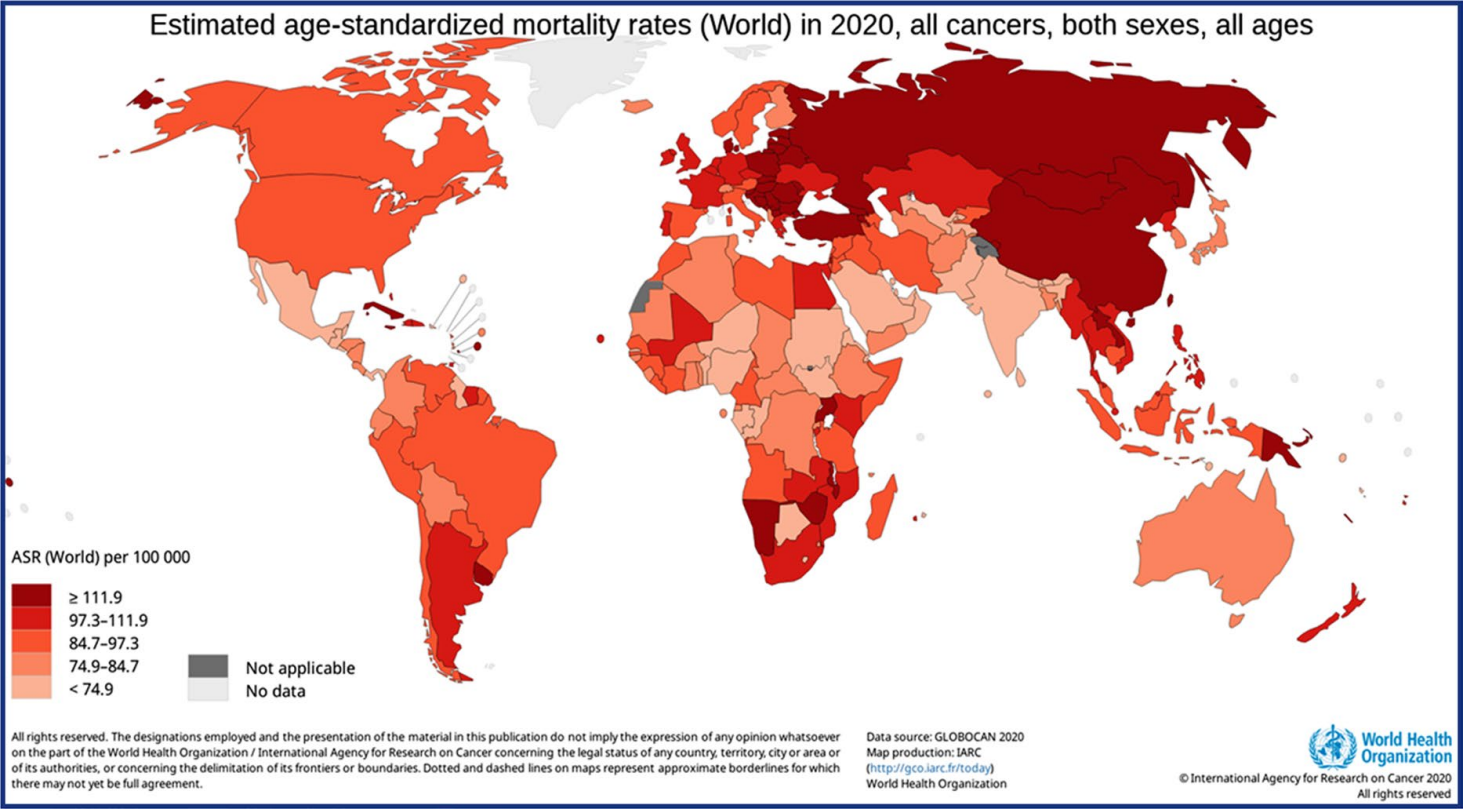
Graphical representation of age-standardized mortality rate among all ages due to different types of cancer in 2020 globally [8]

Moreover, these factors have contributed to a significant increase in cancer cases, i.e., 55%, in developing countries. Whereas, in under-developed countries, the rise of malignant tumors occurs due to the above factors and the lack of sophisticated, reliable systems for disease control and screening facilities [7]. The age-standardized mortality rate due for different types of cancer among all ages in 2020 globally are illustrated in Fig. 1.

Among all cancers, breast cancer (BC) is the highly aggressive form of cancer that develops when abnormal cells start to grow and multiply out of control, resulting in the formation of a lump or mass. According to the published consensus in 2020, approximately 2.3 million BC cases have been recorded. This malady is found to be highly frequent in females (responsible for 12% of female death) compared to males. Additionally, BC is the most prevalent form of cancer globally, ranking as the highest in terms of tumor incidence. It is also recognized as the leading cause of death, followed by lung and colorectal cancer [9]. To get rid of this dreadful malady, researchers have started exploring remedial options for its treatment.

For decades, the common treatment methods used for the eradication of cancers involve intravenous injections of chemotherapeutic medicines, vaccines [10], antineoplastic treatment, surgery, and neoadjuvant therapy (involving targeted/immunotherapy, radiation therapy, hormonal therapy, and chemotherapy). Despite the effectiveness of conventional treatment in regulating cancer, all of these therapies encounter various challenges, including limited specificity, inadequate sensitivity and potential harm to normal cells and tissues [11]. Moreover, these limitations led to the development of multi-drug resistance (MDR) cancer cells in a few cases. MDR is a phenomenon accorded in cancer cells that enables them to withstand the effects of various types of anti-cancer medications. It is characterized by the cell’s ability to survive and proliferate even after exposure to chemotherapy [12]. In fact, the low success rate of effective BC therapy can be attributed to its inherent heterogeneity, which contributes to the emergence of side-effects and multi-drug resistance. These complexities in BC tumor cells make them resilient and difficult to treat [13]. This remains a daunting challenge and a significant burden on humanity.

As a result, researchers are compelled to explore alternative solutions and treatment methods.

One such approach is the use of nanomaterials, which have emerged as an effective and innovative method for combating cancer [14] as these are revolutionary materials with unique chemical and biological properties. In fact, the large surface area, small size, regulated shape and easy amendable nature of these nanoparticles make them engineered multifunctional systems at the atomic/molecular scale that can be amended according to desirable properties. In today’s scenario, nanomaterials have improved the lives of many, especially with chronic health problems. Nowadays, advanced nanomaterials are being designed that are environmentally friendly and can act as a drug carrier for preventing and treating different tumors [15]. Even newly developed nanomaterials, like nano-devices and nano-sensors, are used as implants for uninterrupted health monitoring [16].

Thus, this nanomaterial approach is believed to uncover new possibilities for the early prevention, diagnosis, and medication of global pathogenic diseases and breast carcinoma [17].

The complexities posed by breast cancer (BC), this review take a different approach by focused on the systematic design and development of plant-mediated "biogenic nanoparticles." BC challenges have spurred the scientific community to explore diverse biogenic nanoparticles (NPs). Therefore, this review aims to comprehensively address current BC understanding, covering its various types, stages, histopathology, symptoms, causes, and epidemiology. The discourse further explores conventional BC treatment methods and management techniques. However, the primary emphasis of this review centers on examining biogenic nanomaterials and their crucial role. It also discusses various methodologies for synthesizing nanomaterials sourced from natural origins, particularly within the context of breast cancer research.

## Breast cancer

**Fig. 2:**
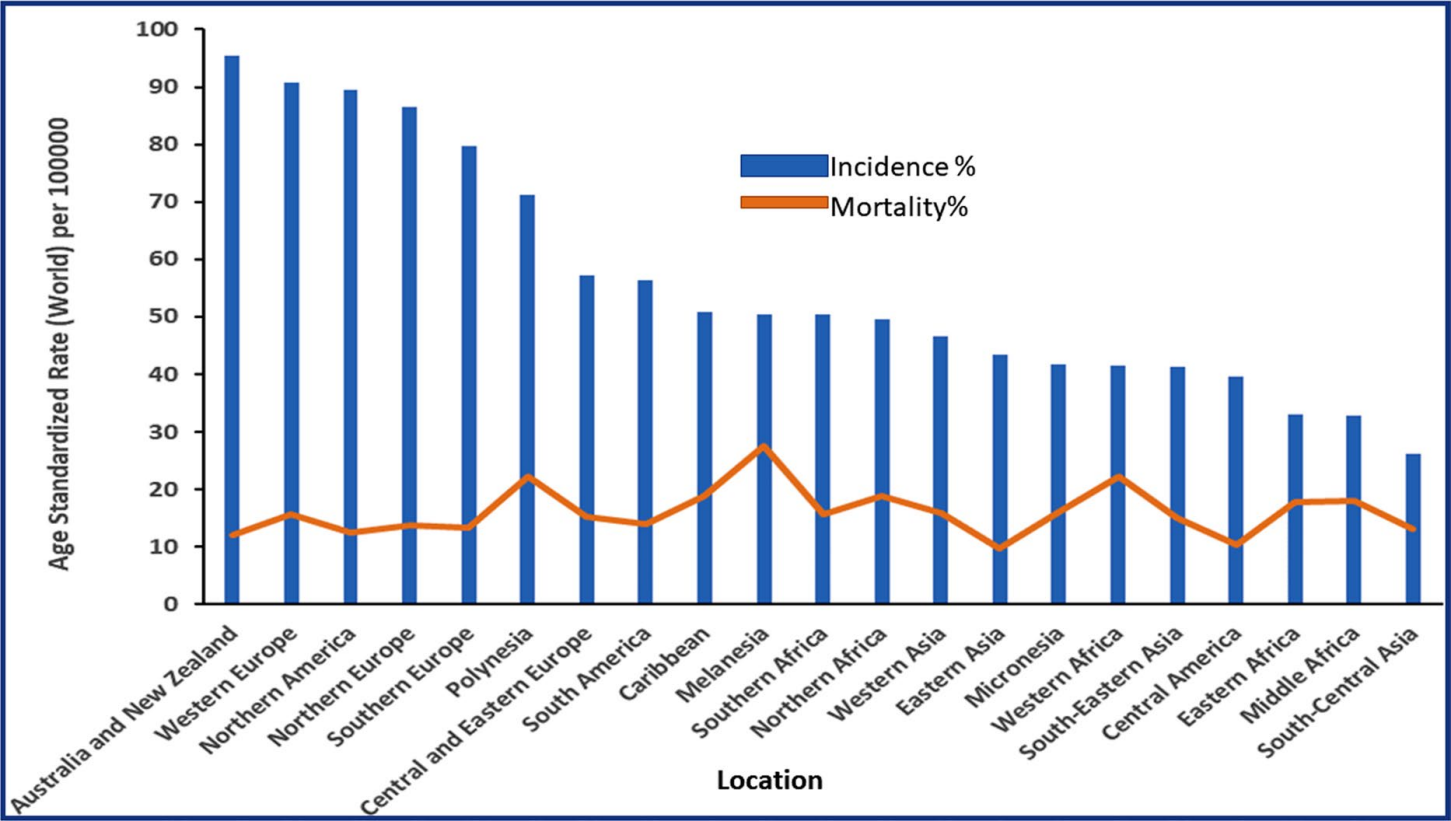
Graphical representation illustrating age standardized BC incidence and mortality statistics worldwide categorized by region (Data source: Ref. [25])

Breast cancer originates in the cells of the breast and occurs when abnormal or mutated cells start proliferating and dividing in an uncontrolled manner, leading to the formation of a lump. This can be identified via physical examination or mammograms [18]. Breast cancer is the traditional term for different BC sub-types with distinct clinical, molecular, and cellular features. Benign breast lumps are usually detected in the breasts that form in lobules or milk ducts are called benign BC, while they can propagate and invade other body parts which is known as metastasis [19]. This ailment is the foremost cause of death among women [20]. In 2020, 2,261,419 (11.7%) new cases of BC were diagnosed with 684,990 (6.9%) deaths around the globe, as represented in Fig. 2. Breast cancer incidence rates vary significantly across different countries and regions, with some Asian and African countries having rates below 40 cases per 100,000 females. In contrast, Australia, New Zealand, North America, and parts of Europe had rates exceeding 80 cases per 100,000 females. Although the variation in mortality rates was minor, transitioning countries still bear a disproportionate burden of BC deaths compared to countries that have already transitioned. By 2040, it is estimated that the global burden of BC will escalate even further, with over 3 million new cases and 1 million deaths projected annually. This increase is primarily attributed to population growth and ageing, highlighting the urgent need for preventive measures, early detection, and improved treatment options to address this growing public health challenge [21]. Invasive BC spreads from a specific site to surrounding tissues in the breast or other parts of the body. According to the American Cancer Society, invasive BC accounts for approximately 80% of all BC cases [22]. Breast cancer cases increase with age, with 95% of new cases occurring in women above 40 or older. The vast majority, i.e., 99% of women, face social embarrassment and a negative impact on daily activities, including children's studies. This dreadful sickness has been diagnosed in every country and every age after puberty in females. Moreover, it also increases the probability of developing cancer in later stages of life [23]. All tumors detected in breasts are not the same, and the type of BC varies according to their origin [24].

### Types of breast cancer

Breast cancer manifests in various forms, each with unique characteristics and stages [26]. One of the most common types of BC is ductal carcinoma in situ (DCIS), which accounts for 15% of BC cases and is regarded a pre-cancerous stage [27]. It involves abnormal cell growth within the milk duct lining but does not spread beyond the ducts. However, if left undetected, it can progress to invasive cancer [28]. Ductal cancer, which comprises 80% of all cases, starts in the milk ducts and invades adjacent breast tissues, classifying it as stage I. Lobular cancer in situ (LCIS) involves uncontrolled cell division in the lobules of the breast, but it does not invade surrounding tissues, representing a pre-cancerous condition. Regular mammograms are necessary to monitor LCIS for potential cancer development [29]. Lobular cancer, known as invasive lobular carcinoma (ILC), arises in the breast's lobules and is less likely to form a palpable lump, making it harder to detect [30]. This type often occurs in females with ages between 40–50 and accounts for 10–15% of all breast cancer cases. Triple-negative breast cancer (TNBC) is an aggressive subtype lacking the estrogen receptor (ER), progesterone receptor (PR), and human epidermal growth factor receptor 2 (HER2). It affects younger women, those with a family history, and women of African or Hispanic descent [31]. Inflammatory breast cancer is characterized by carcinoma cells obstructing lymph vessels, leading to breast redness, swelling, and other symptoms. It is aggressive and accounts for 5% of all cases, often diagnosed at stage III or IV [32]. Paget's disease of the breast affects the skin and nipple, presenting as an itchy, scaly rash. It accounts for 1–3% of cases and starts in the ducts [33]. Angiosarcoma is a rare and aggressive cancer that arises from blood or lymphatic vessel cells. It can occur in various body parts and spreads rapidly, posing treatment challenges [34]. Phyllodes tumor is a rare tumor in the breast's connective tissue, which can be benign or malignant. It originates from the breast's stroma and can become malignant if untreated, especially in individuals with Li-Fraumeni syndrome, an inherited genetic disorder [34].

**Fig. 3:**
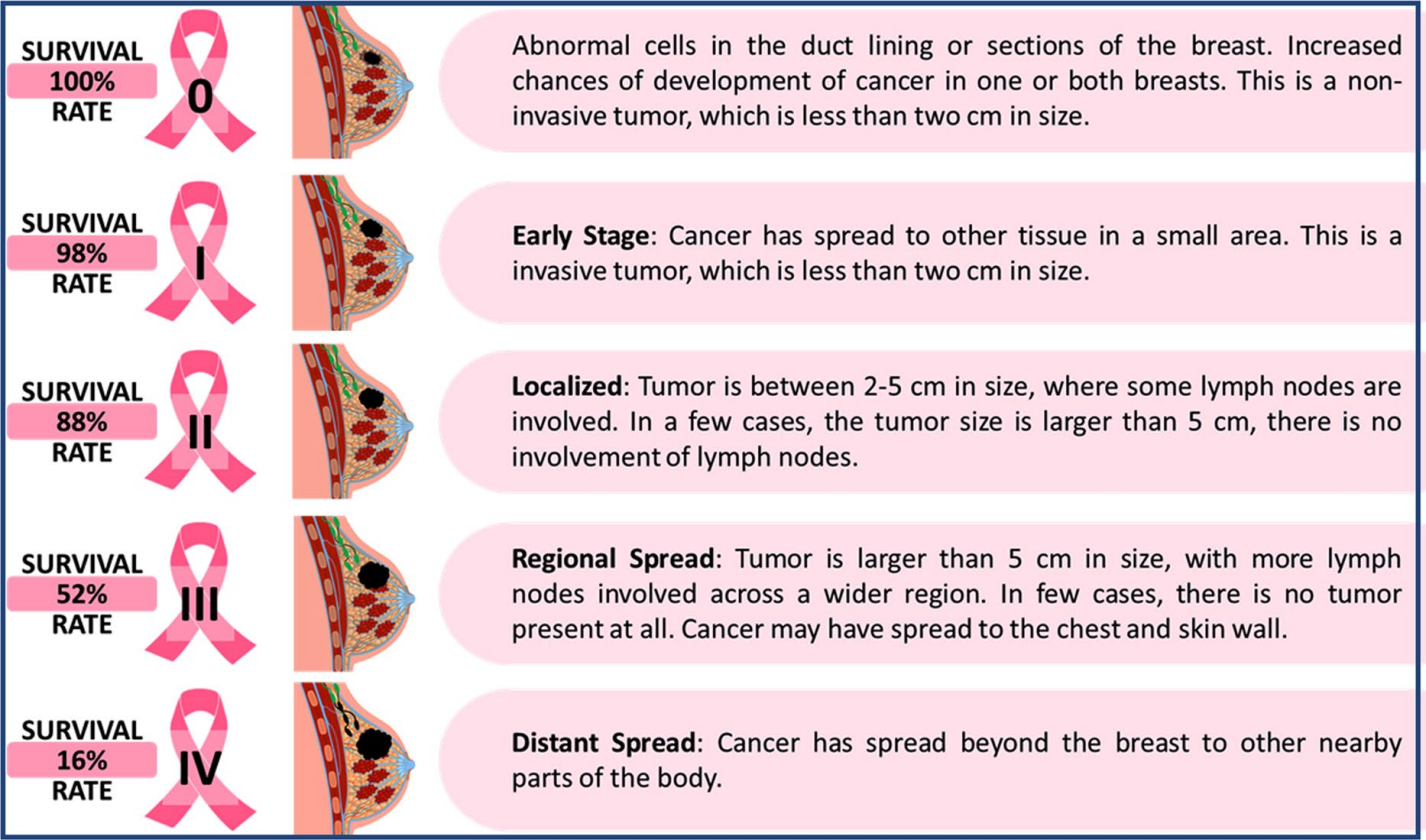
Different stages of breast cancer

Upon the detection of breast cancer, various tests are executed to decide the stage and impact of BC treatment. The International Union for Cancer Control (UICC) describes breast cancer stages as the Tumor, Node and Metastasis (TNM) BC staging: Stage 0, Stage I, Stage II, Stage III and Stage IV, as shown in Fig. 3. [35] .

## Signs and symptoms of breast cancer

Carcinoma in the breast usually appears as a lump that is thick, dimpled, and painless but sensitive to the touch. Thus, even if there is no pain associated with the lump in the breast, women need to assess it and consult a doctor. This should not be delayed for more than two months [36]. The common symptoms of BC involve: swelling of the breast, erythema, alterations in size or form of the breast, the skin of the breast may develop dimples or puckers that resemble an orange peel, discharge from the nipple that may be either bloody or clear, alteration in the appearance of the nipple (i.e., flaking or inverted), scorching or persistent scratching on the breast, and formation of lymph nodes under the arm or around the collarbone that are appear swollen or lumpy [37].

## Etiology of breast cancer

The progressive elevation in the mortality rate of women has promoted the identification of different aspects associated with an increased risk of BC. That could play a substantial role during general health screening of women. Additionally, different risk factors have been identified and broadly divided into various factors like: Demographic Factors, Reproductive Factors, Heredity Factors, and Family History, Breast Related Factors, Hormonal Factor, Lifestyle Factor and Other Risk Factors that are briefly discussed below.

### Demographic factor

#### Age

One of the primary breast cancer risk factors is age. As a person ages, their risk of developing breast cancer increases, with most cases being discovered in women over 50. This is because as a person ages, their DNA becomes more vulnerable to damage and mutations, which can cause out-of-control cell growth and the onset of cancer in breast cells. The rate of breast cancer touches its peak in menopause; however, it gradually decreases after menopause. Breast cancer in younger women is invasive, and the survival rate is very low [38].

#### Gender

Gender is another crucial risk factor for breast cancer. Breast cancer is much more common in women than in men, as 1 out of 8 women has a lifetime risk of developing breast cancer in contrast to men, which is 1 out of 833. In fact, women have much more breast tissue than men and contain a large number of hormones that can trigger the growth of cancer cells [39]. This is why breast tissue in women is more prone to cell changes that can lead to cancer.

#### Blood group

Evidence suggests women with blood group A and Rhesus positive factor have a higher risk of developing breast cancer. In comparison, the females with blood group AB and Rhesus negative have a lower risk of developing breast cancer [40]. There is no direct evidence that a women’s blood group is a cause of breast cancer. At the same time, some researchers have suggested that some blood groups may be linked with a slight increase or decrease in the incidence of some cancers, including breast cancer [41].

### Reproductive factors

Various reproductive factors have also been found to be associated with the surging of breast cancer, which include:

#### Age of menarche

Researchers suggest that the early onset of menstruation, also known as early menarche, is associated with breast cancer. Thus females who experience this are at higher risk of developing breast cancer. For instance, females who begin menstruating before age 12 have more chances of developing breast cancer than those who start in the later stages of life**.** This increased risk is believed to be linked to hormonal changes during puberty. After puberty, the ovaries begin to produce progesterone and estrogen hormones, which stimulates the uncontrolled growth of breast tissues and causes genetic mutations that lead to the development of breast tumour later in life [41]. A longitudinal study was conducted on 200 schoolgirls aged 7 to 17 years, with three assessments carried out at intervals of 1.5 years. The aim was to investigate the relationship between serum progesterone concentrations and ovulatory cycles. Ovulation was defined as a serum progesterone concentration exceeding 6.4 nmol/l (2.0 ng/ml) in the latter part of the menstrual cycle.

In contrast, ovulatory cycles were characterized by concentrations below 1.6 nmol/litre (0.5 ng/ml). Early menarche was associated with an early onset of ovulatory cycles. The duration from menarche until 50% of the cycles became ovulatory was approximately 1, 3, and 4.5 years for girls with menarcheal ages below 12.0, between 12.0 and 12.9, and equal to or greater than 13.0 years, respectively. Furthermore, girls with early menarche (below 12.0 years) exhibited higher serum estradiol concentrations but lower testosterone and dehydro-epiandrosterone concentrations than those with later menarche. These findings suggest that the increase in adrenal androgen secretion primarily relates to chronological age rather than the timing of menarche. The observation of early ovulation following early menarche contradicts the estrogen-window hypothesis, which proposes a longer duration of an-ovulatory cycles to explain the elevated risk of breast cancer associated with early menarche [42].

#### Age of menopause

According to published studies, women who go through menopause later in life (after age 55) have a slightly increased risk of developing breast cancer than women who go through menopause earlier in life (before age 45). This is believed to be the case because oestrogen and progesterone, produced by the ovaries and decreased after menopause, may increase the risk of breast cancer in women with prolonged exposure. The age of menopause in women above 55 years is associated with an upsurge risk of breast cancer [43]. A comprehensive analysis was conducted utilizing data obtained from 117 different epidemiological studies, encompassing 118,964 women diagnosed with invasive breast cancer and 306,091 women without any indications of the disease. Additionally, for each year older at menopause, the risk increased by a smaller amount of 1.029 (95% CI 1.025–1.032; P < 0.0001). Notably, none of the individuals included in the study had a history of utilizing menopausal hormone therapy. The primary objective of this analysis was to determine the adjusted relative risks (RRs) associated with two crucial factors, namely, menarche and menopause, concerning breast cancer in its entirety [44].

#### Full-time pregnancy

Pregnancy at a young age can reduce the risk of developing breast cancer later in life. There is scientific evidence suggesting that shorter pregnancy periods could increase the vulnerability to breast cancer, especially before menopause. This increased risk can be linked to hormonal stimulation and the early-stage growth of breast tissue during pregnancy, as there is limited opportunity for the subsequent maturation that typically occurs in the later stages of pregnancy [45]. Among parous females, every childbirth decreases the risk of PR + and ER + cancers by 10%, and females who give birth in later stages of life are at a higher risk of developing breast cancer [46]. Studies have shown that two or more childbirths and lactation for more than 12 months can decrease the risk of 7% and 4.3% of breast cancer, respectively [47].

#### Nulliparity

The medical term "Nulliparity" refers to a female who has never given birth to a child. It also applies to women who have never given birth or carried a foetus over the 20-week mark. Infertility, other medical issues, or a person's choice can all lead to nulliparity. During pregnancy, the progesterone and estrogen hormone level increases significantly. It believed that these hormones induce different changes to breast tissues which may contribute to a prospective cohort study, known as the Malmö Diet and Cancer Study involved the enrollment of 17,035 female participants. These individuals were subsequently tracked and monitored through linkage with the Swedish Cancer Registry until December 31, 2004. Throughout the study, a total of 622 newly occurring cases of breast cancer were identified and diagnosed among the participants. The study found that Nulliparity was linked to an increased risk of breast cancer, although the association was not statistically significant (relative risk of 1.39 with a 95% confidence interval of 0.92–2.08). Nulliparity was also associated with specific characteristics of breast cancer, including larger tumour size (> 20 mm), high levels of Ki67, high levels of cyclin D1, grade III tumours, and HER2-positive tumours. Additionally, older age at first childbirth (above 30 years) was slightly associated with an increased risk of breast cancer (relative risk of 1.39 with a confidence interval of 0.94–2.07). Late first childbirth was significantly related to lobular type tumours, grade III tumours, high levels of cyclin D1, and low levels of p27. In summary, the study suggests that nulliparity and late first childbirth are associated with breast cancer cases that exhibit more aggressive characteristics [48].

### Genetic factor

Breast cancer is associated with genetic mutations in specific genes such as BRCA1 and BRCA2. However, the connection between mutations in other genes like BRCA1 and BRCA2 and rare syndromes is caused by mutations in TP53, STK11, PTEN, CDH1, NF1 OR NBN. These rare genetic syndromes may be associated with an increased risk of developing breast cancer, but the exact nature of this relationship is still unknown. The lifetime risk of developing breast cancer is increased to 40–80% in individuals who carry mutations in the BRCA1 and BRCA2 genes. These mutations can also lead to the development of several inherited cancer syndromes like Li-Fraumeni syndrome, Cowden syndrome, Peutz-Jeghers syndrome, and E-cadherin mutations [49].

According to a study, carriers of BRAC1 mutations have a 55% to 65% risk of developing breast tumours by age 70, while carriers of BRAC2 mutations have a 45% chance of emerging [50]. Individuals who carry a mutation in the BRCA1 gene have a 72% incidence of developing breast tumours by the age of 80. In comparison, those who carry a mutation in the BRCA2 gene have a 69% incidence of developing breast tumours by the same age [51]. The emergence and onset of breast cancer may also induce changes in human interferon α-2b and other risk factors. Additionally, the case-controlled study revealed that MMP-2C allele polymorphisms, specifically MMP-2c-735-T, MMP3 5A, and MMP9 T allele, are also associated with the upsurge of breast cancer [52].

### Family history of breast cancer

It is true that having a family history of breast cancer increases the risk of developing breast cancer in females. If a first-degree relative has breast cancer, such as a mother, sister, or daughter, the risk goes up [53]. For decades, researchers have stated that a family history of breast cancer is one of the critical factors related to breast cancer. Females with a family history of breast cancer, especially if there are two or more cases among females under 50 years old or at any age. Investigating the life history of 21 women with a family history of breast cancer highlights the significant increase in breast cancer risk for individuals with such a history. The report shows that these women have approximately 11 times higher chances of developing breast cancer compared to those who do not have a family history of the disease [54]. According to research analysis, women who have a family history of breast cancer and are diagnosed with the disease are more likely to have mutations in the BRCA1, BRCA2, and TP53 genes. The investigation report indicates that out of the 36 cases with a family history of breast cancer, 17 had mutations in one of these three genes. In contrast, only three out of the 63 cases without a family history had a mutation in one of these genes [55].

### Breast-related factors

Breast-related factors include any change in physical or biological characteristics that can negatively affect a person’s health and medical conditions. Some examples of breast-related factors have discussed below:

#### Breast size

Breast size is not a direct cause of breast cancer. However, some studies have suggested that women with large breasts may have a greater chance of developing breast cancer than small breasts. The research revealed that women with large breasts (those who underwent mastectomy with weight greater than 800 g) had a significantly larger tumour than those with small breasts (P = 0.019, Mnn-whitney test) [56]. The analysis revealed no noteworthy difference in tumour grade between large and small breast size, as indicated by Kendall's tau-b value of 0.011 and a p-value of 0.57 [57].

#### Breast density

Breast density is a term used to define the percentage of different types of tissues in the breast like fat, glandular tissues, lobules and connective tissues. Mammography is a technique that has been extensively used to detect breast density and abnormalities in the breast. According to research, it is estimated that a reduction in breast density to 12.29% or less in all females can avert the chances of breast cancer up-to 48.6% [57].

### Hormonal factor

Various hormonal factors are believed to be involved in the development of Breast cancer in females, like endogenous hormones, i.e., estrogen and progesterone, produced by ovaries.

It is a medical approach that involves the administration of hormones that a female body no longer produces in sufficient amounts. The risk appears, particularly for women who use a combination of estrogen and progestin. The incidence of breast cancer linked with HRT use varies depending on several aspects, including duration of HRT use, type of HRT use, the age of the woman when she starts HRT, and personal as well as family history [58]. Research survey of 51 epidemiological causes, the use of HRT is associated with a slight increase in the incidence of breast cancer, with a relative risk (RR) of 1.023 and confidence interval (Cl) of 1.011 to 1.03 [59]. However, a study involving 30–39 years women revealed that the current use of HRT is associated with higher mortality rates and enhanced risk of breast cancer. A significant number of women started HRT at ages 40 to 44, 45 to 49, 50 to 54 and 55 to 55; the RR were similar for each set. For women who initiated HRT between ages 60 to 69 years, few cases of oestrogen-only menopausal hormonal therapy (based only on 135 exposed cases) resulted in non-significant excess risks during 5 to 14 of the current use (RR 1.08; 95% CI 0.90–3.1). However, oestrogen–progestagen HRT resulted in significant excess risk during the period (RR 1.75; 95% CI 1.48–2.06) [60]. Prolonged exposure to these hormones often leads to an increased risk of breast cancer. Factors such as early onset of menstruation, Nulliparity, delayed onset of menopause and older age at first pregnancy can result from prolonged exposure to these hormones.

### Lifestyle factors

Several lifestyle factors have been associated with an increasing number of breast cancers which include:

#### Alcohol consumption

There is strong evidence that drinking alcohol increases the menace of breast cancer. According to the world health organization, alcohol consumption accounts for 7 out of every 100 new breast cancer cases. Collaborative analysis of individual data from 53 epidemiological studies that alcohol consumption has been linked with the upsurge in breast cancer that is statistically significant at levels as low as 5 to 9.9 g per day, equivalent to 3 to 6 drinks per week (RR = 1.15; 95% CI 1.06–1.24; 333 cases/100,000 persons/year [61]. Reducing alcohol consumption is a significant step that can significantly help in decreasing the incidence rate of breast cancer [62].

#### Smoking

Although the epidemiological sign on the role of active smoking in breast tumour danger is unreliable, the current literature survey supports a modest link between active smoking and breast cancer risk [63]. Cox proportional hazard models were used to estimate hazard ratios (HR) and 95% confidence intervals (CI) to determine the relationship between smoking and breast cancer risk. This study included 89,835 females aged 40–59 and tracked them for an average of 22 years. Of these females, 6,549 were found to have breast cancer. Cox proportional hazard models were used to estimate hazard ratios (HR) and 95% confidence intervals (CI) to determine the relationship between smoking and breast cancer risk. The results showed that the number of years smoked before a female's first full-term pregnancy was associated with a higher risk of breast cancer compared to the number of years smoked after pregnancy and during adjuvant radiation therapy [64]. Specifically, the hazard ratio (HR) for smoking 5 years pre-pregnancy compared to not smoking was 1.18, with a 95% confidence interval (CI) of 1.10–1.26. These findings provide strong evidence supporting the involvement of cigarette smoking in the development of breast cancer [65].

#### Obesity and overweight

According to published literature, substantial evidence suggests obesity and overweight increase the risk of developing breast cancer in females. One possible explanation for this relationship is that adipose tissue or body fat produces hormones like estrogen, which further stimulate the growth of breast tumour cells by the activation of the oncogenic signalling pathway via leptin, a hormone produced from adipocytes, that promote the cell progression and metastasis of breast cancer cells [66]. N-glycosylation is a process in which sugar molecules are added to proteins to form glycoproteins. Research studies have suggested that alterations in N-glycosylation may be associated with the development and progression of breast cancer [67]. A study was conducted to investigate the impact of obesity on survival rates among 859 cases of newly diagnosed breast cancer. Results of the study revealed that obese females were at higher risk of 1.7 and 1.8 fold for stage III/IV cancer and grade ¾ tumours, respectively [68].

#### Coffee

Numerous studies have investigated the connection between drinking coffee and breast cancer risk, but results have been unreliable [69]. According to a study conducted in Hong Kong and China, postmenopausal women and women with a BRCA1 mutation who consumed brewed coffee showed a decreased risk of breast cancer (AOR = 0.48, 95% CI = 1.10–2.03). However, those who consumed instant coffee had a significantly increased risk of breast cancer (AOR = 0.48, 95% CI = 1.10–2.03) [70].

#### Diet

Published literature has provided strong evidence that diet plays a vital role in the progression and prevention of breast cancer. Out of a total of forty patients, 22% reported changes in their diet. When compared with the control group, patients were found to have significantly higher rates of avoiding alcohol (P = 0.0005) and three specific food items, namely smoked food (P = 0.04), milk (P = 0.02), and other dairy products (P < 0.0001). Half of all patients were classified as overweight or obese based on their BMI. The study found that breast cancer treatment accounted for 5.7% of the variation in dietary changes, with chemotherapy being the only significant predictor (P = 0.04) [71]. In another study, a meta-analysis was conducted to explore the relationship between various dietary protein sources and the risk of breast cancer, as different protein sources may have distinct effects on the human body. PubMed and other databases were searched up to December 2015. The findings indicate that consuming higher amounts of total red meat, fresh red meat, and processed meat could potentially increase the risk of premenopausal breast cancer [72].

#### Vitamin D

Numerous studies have investigated the potential association between vitamin D and breast cancer. It is an essential vitamin which promotes healthy cell growth, regulates the immune system, and maintains strong bones and teeth. Vitamin D significantly impacts the physiology of adipocytes and glucose metabolism, even though it regulates cellular processes such as proliferation, differentiation, and adhesion, which may be associated with breast cancer development [73]. In a Canadian observational trial, 8,155 participants were directed to reach 25-hydroxyvitamin D (25OHD) levels of over 100 nmol/l using vitamin D3 tablets. The study aimed to investigate the relationship between achieved serum concentrations of 25OHD and blood pressure. The result obtained revealed that the mean baseline 25OHD level was 87 ± 37 nmol/l, and the final level was 113 ± 39 nmol/l.

Moreover, the assessment unveiled that about 33% of participants took more than 8,000 IU of vitamin D3 daily. Published literature that utilized data from randomized controlled trials (RCTs) of Vitamin D supplements and one cohort study showed a significant and inverse correlation between serum concentration of 250HD and the risk of breast cancer [74]. Another study reported about the pooled cohort, which encompassed 5038 females, out of which 77 were diagnosed with breast cancer during the study. Multivariate Cox regression analysis showed that women with 250HD levels of 150 nmol/L had a risk ratio for breast cancer of 0.20 (95% CI 0.05–082) compared to those with 250HD levels of 50 nmol/L [75].

### Other risk factors

#### Air pollution

According to our findings, there may be a heightened risk of breast cancer associated with exposure to nitrogen dioxide (NO_2_), an indicator of pollution from different sources of motor vehicles. The fact that certain airborne pollutants have a stronger association with hormone receptor-positive breast tumours suggests that they may have a role in the development of this disease. Some studies have suggested that air pollutants like Nitrogen dioxide (NO_2_ and NO_x_) [76], fine particulate matter (PM10 and PM2.5) [77], and polycyclic aromatic hydrocarbons (PAHs), polychlorinated biphenyls (PCBs) [78] and dioxins are associated with breast cancer incidences [79].

#### Radiation

Exposure to ionizing radiation, particularly during adolescence or early adulthood, is also a known risk factor for breast cancer. The risk of developing breast cancer increases with increasing doses of radiation and can persist for many years after exposure. Radiation therapy is usually used to treat different cancers, but it can also increase the risk of developing breast cancer among females diagnosed with cancer. This is because the high-energy radiation used to destroy cancer cells can simultaneously damage the healthy cells in the breast, leading to genetic mutations that could eventually lead to the development of breast cancer [80]. The risk of developing breast cancer due to radiation exposure depends on numerous factors, including the dose and duration of radiation therapy, the age at which radiation therapy was received and other incidence factors such as a family history of breast cancer. During a follow-up period of 1.8 million females per year, out of 77,527 women (35,000 of them exposed to radiation), a total of 1502 breast cancer cases were detected [81].

## Histopathology and pathophysiology of breast cancer

Histopathology plays a crucial role in comprehending the pathophysiology of breast cancer. Histopathological analysis involves the microscopic examination of tissue samples, providing valuable insights into the structural changes occurring in the breast cells. Through this examination, the type of breast cancer, its aggressiveness, and the extent of spreading can be determined. The pathophysiology of breast cancer involves a complex interplay of genetic mutations, Deoxyribonucleic acid (DNA) damage, environmental factors, and hormonal influences [82]. In the pathophysiology of BC, initiation is the first stage, where genetic mutations occur in breast cells. These mutations can be inherited (such as BRCA1 and BRCA2 gene mutations), or they can result from hormone imbalances or exposure to carcinogens. These mutations affect genes involved in regulating cell growth and division [83]. Following initiation, the mutated cells undergo further changes in a process known as promotion. Growth factors, hormones, and signalling pathways interact to promote the aberrant growth and division of these cells. This leads to the proliferation of cancer cells within the breast tissue [84]. Angiogenic factors released by the cancer cells stimulate the development of a network of blood vessels, providing the growing tumor with necessary nutrients and oxygen [85]. Invasion and metastasis are critical stages in breast cancer progression. Cancer cells develop the ability to invade surrounding tissues and enter lymphatic channels or blood vessels. Through this process, they can spread to distant sites in the body, such as lymph nodes, bones, liver, lungs, or brain, forming secondary tumors through metastasis [86].

The hormone receptor status of breast cancers also contributes to their pathophysiology. Breast cancers can be classified based on the presence of hormone receptors, particularly ER and PR. ER-positive and PR-positive breast cancers have receptors on their cell surfaces that bind to estrogen and progesterone, respectively, promoting tumour growth [87].

In summary, histopathology provides a detailed understanding of the microscopic changes occurring in breast tissue, while the pathophysiology of breast cancer involves a complex series of genetic mutations, DNA damage, environmental influences, and hormonal factors that contribute to the initiation, promotion, angiogenesis, invasion, metastasis, and receptor status of BC cells.

## Evaluation of breast cancer

Firstly, a comprehensive medical history is collected to assess risk factors, family history, and prior breast-related diseases or treatment. A physical examination is then conducted to look for visible signs or symptoms of breast cancer, such as lumps, changes in breast size or shape, nipple abnormalities, or skin changes. All Imaging test have been discussed in detail as follow.

### Mammogram

**Fig. 4:**
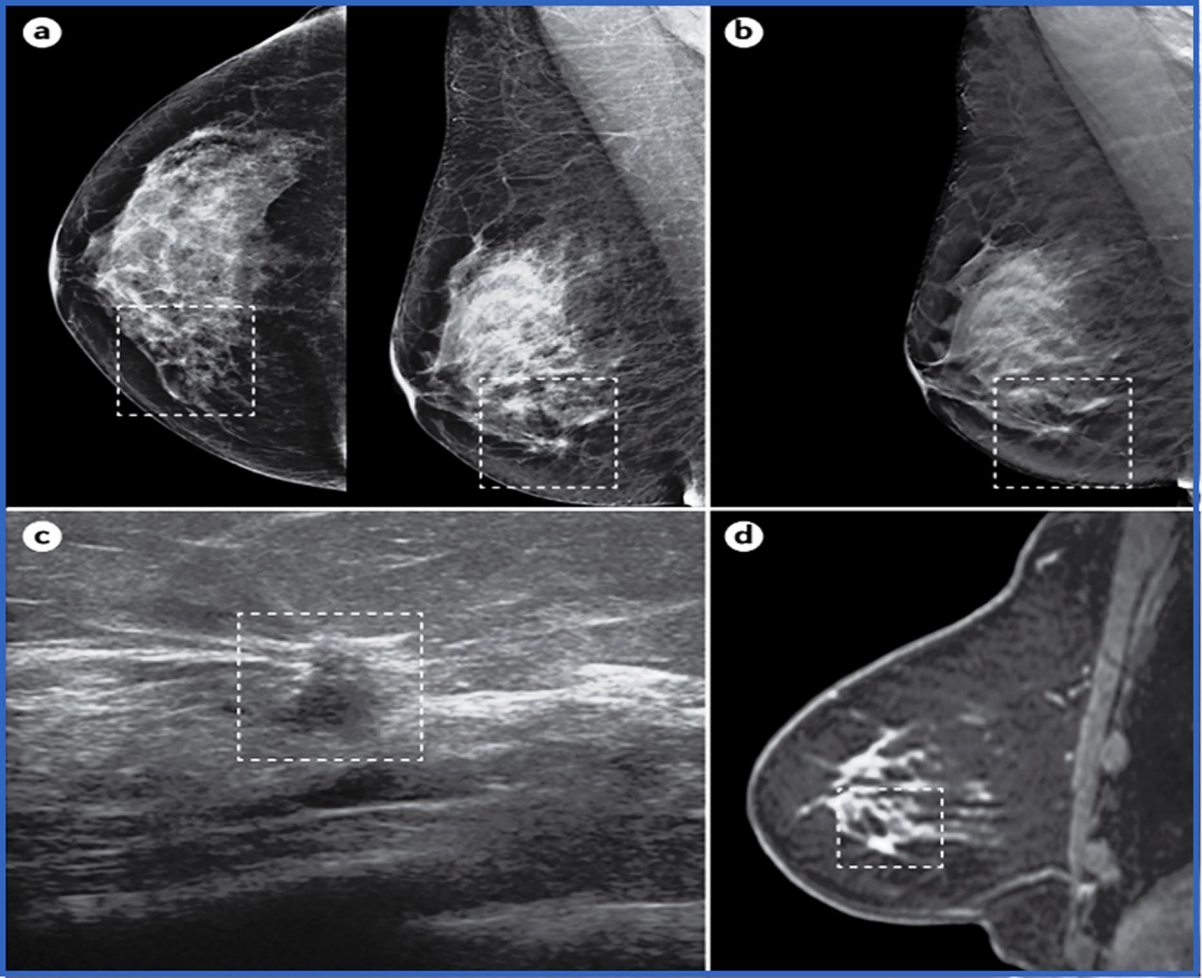
Breast cancer imaging. A postmenopausal woman 53 years of age with no family history and no clinical findings underwent routine breast screening with mammography, which detected a lesion in the right breast (panel **a**, cranio-caudal view (left) and mediolateral oblique view (right)). The images were also acquired with digital breast tomosynthesis, which showed a small spiculated lesion in the lower inner quadrant (panel **b**, mediolateral oblique view). The lesion was investigated with ultrasonography (panel **c**), and biopsy confirmed an invasive ductal carcinoma on histology. MRI showed the enhancing spiculated mass (panel **d**). The tumour is indicated within the dashed lines in each panel. [89]

It is a specific kind of imaging technique used to detect and diagnose breast cancer. It involves capturing a low-intensity X-ray of the breast tissue to look for any abnormalities, such as lumps, calcifications, or other alterations that could be signs of breast cancer. The primary objective of population screening is to detect a disease at an early stage using an accurate, non-invasive, and acceptable test to treat it effectively. The breast is gently crushed between two plates to spread out the breast tissue during mammography and improve visualization and proper interpretation of the X-ray pictures. This method is proclaimed to be the secondary prevention approach for breast cancer treatment. Albalawi et al. (2022) reported a model which involved five main phases, i.e., preprocessing, segmentation, feature extraction, feature selection, and classification. In preprocessing, the mammogram image undergoes contrast-limited adaptive histogram equalization (CLAHE) and median filtering for initial enhancement. Next, the image is segmented to isolate regions of interest. From these regions, relevant features are extracted to capture distinctive characteristics of potential tumours. A feature selection process is applied to identify the most informative features to improve the model's efficiency. Finally, based on the selected features, a classification algorithm is employed to classify the mammogram image as either cancerous or non-cancerous. By integrating these five phases, the proposed model aims to enhance the complexity of breast cancer detection using mammogram images [88]. Moreover, Mammography-related randomised controlled studies have shown strong evidence in favour of population screening, showing a significant 20% decrease in breast cancer mortality for those invited to participate in screening [89]. Regular mammograms are recommended for early detection, particularly for women over 40 or those with an increased risk of breast cancer. Figure 4 shows the mammography imaging of undiagnosed right breast lesions of postmenopausal women.

### Ultrasound

Breast ultrasonography creates digital images of the breast's interior using sound waves and their echoes. It reveals breast alterations that are difficult to be noticed via mammograms, such as fluid-filled cysts. This technique is beneficial in evaluating breast lumps or abnormalities detected during a physical examination or mammogram. Ultrasound helps distinguish solid masses (which may become cancerous) and fluid-filled cysts (typically non-cancerous). A study reported scientific advancement in breast ultrasound image analysis by introducing a novel approach combining Active Contour and Texture Feature Vectors. Integrating these models enables the extraction of discriminative features, leading to improved accuracy in detecting breast cancer. The evaluation results on the Breast Ultrasound Images dataset confirm the superior performance of our proposed method compared to existing algorithms [90]. In the subsequent phase of model training using the publicly available breast ultrasound image dataset (BUSI), multiple machine learning (ML) models were considered. Through experimental analysis, it was determined that features related to shape, texture, and histogram-oriented gradients are particularly informative for the predictive modeling task. Ensemble learners such as random forest and gradient boosting classifiers notably performed in various evaluation metrics. These metrics include accuracy, the area under the curve, F1-score, and the Mathews correlation coefficient. The proposed approach achieved state-of-the-art results on the BUSI dataset, with accuracy, the area under the curve, F1-score, and Mathews correlation coefficient values of 0.974, 0.97, 0.94, and 0.959, respectively [91].

**Fig. 5:**
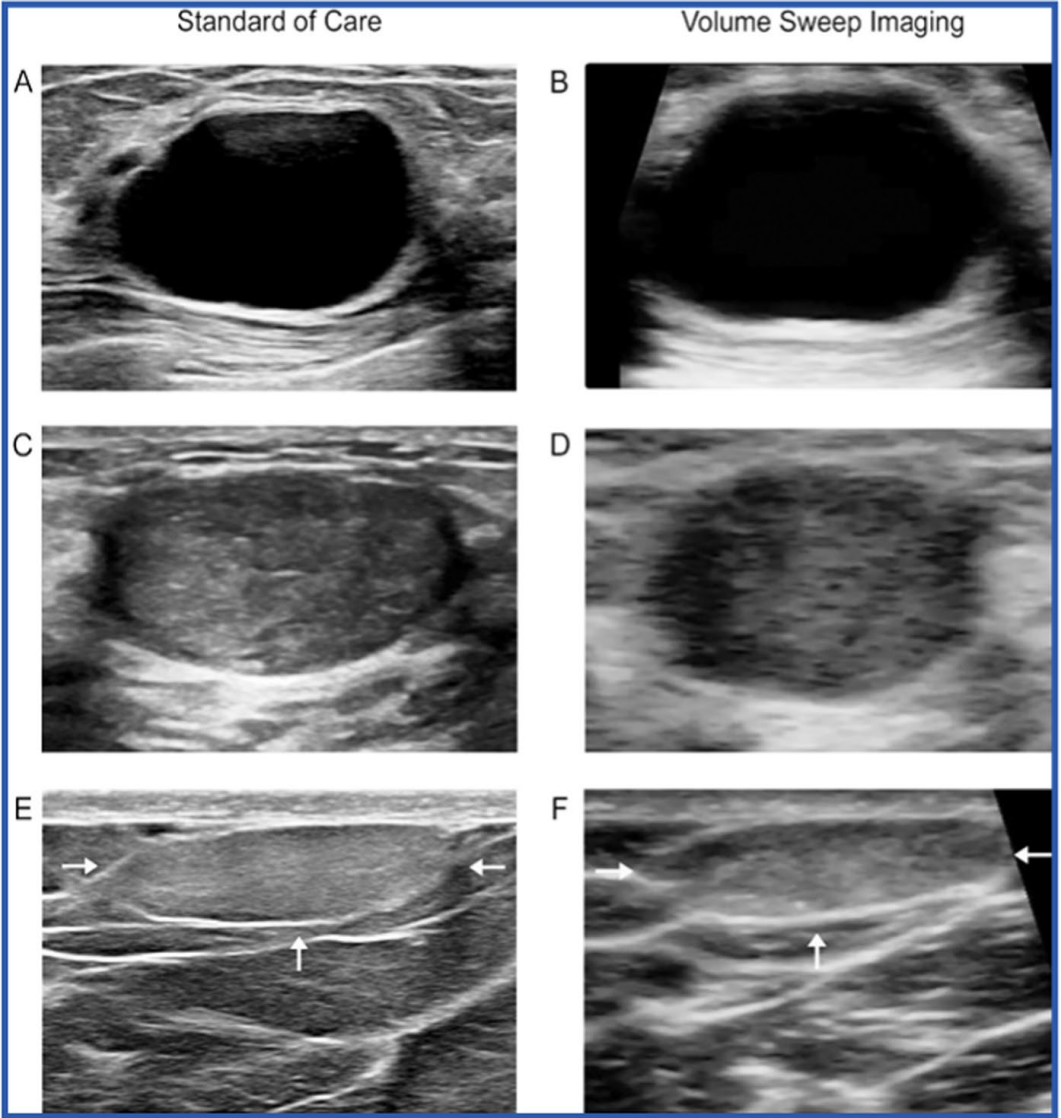
Benign breast lumps on standard of care ultrasound and volume sweep imaging (VSI). Benign anechoic cyst with posterior acoustic enhancement seen on standard of care ultrasound (**A**) and VSI (**B**). Hypoechoic well-circumscribed oval fibroadenoma seen on standard of care ultrasound (**C**) and VSI (**D**). Isoechoic or hyperechoic lipoma (arrows) in the superficial breast tissue on standard of care ultrasound (**E**) and VSI (**F**) [92]

Another study used volume sweep imaging (VSI) ultrasound protocol to evaluate palpable breast lumps shown in Fig. 5. One of the aims of utilizing this approach was to increase the accessibility to breast ultrasound, as the operations of this approach can be performed after minimal training without prior ultrasound operating experience. In this study, the group of medical students utilized the VSI protocol to examine a total of 170 detectable lumps in breast cancer patients. The VSI technique showed 100% specificity and 97% sensitivity for diagnosing breast masses. Compared to the standard of care, this resulted in a significant agreement of 97.6%, as shown by Cohen's value of 0.95 (P 0.0001). Moreover, a 100% success rate of cancer identification was achieved when the tumour was presented as a sonographic mass. Additionally, the agreement between VSI and the standard of care was determined to be high in evaluating the characteristics of the masses, with an 87% concordance in Breast Imaging-Reporting and Data System assessments. This was further supported by a Cohen's κ value of 0.82 (P < 0.0001) [92].

### Magnetic resonance imaging (MRI)

MRI is an imaging technique commonly used to detect and evaluate breast cancer. MRI uses radio waves and a strong magnetic field to produce precise images of the breast tissue. MRI is primarily utilized as an adjunctive modality with mammography or ultrasound for breast screening. Its primary application lies in assisting the assessment of women who have already received a breast cancer diagnosis, aiding in determining tumour size, identifying additional tumours within the breast, and evaluating the contralateral breast for potential tumours. For select women at a high risk of developing breast cancer, a screening MRI and an annual mammogram are recommended. Furthermore, breast MRI does not provide visualization of calcium deposits, referred to as micro-calcifications, which can serve as indicative markers of breast cancer [93]. According to prospective trials, annual supplemental MR imaging in conjunction with mammography has consistently demonstrated a significant increase in sensitivity compared to mammography alone, typically doubling sensitivity. Moreover, the combined approach generally achieves sensitivities surpassing the threshold of 90%. Among a total of 20,053 screening episodes, the cancer detection rate for breast cancer was 14.0 per 1000 (95% confidence interval [CI] = 12.4 to 15.7). The sensitivity of mammography was significantly lower than that of MRI plus mammography (40.8%, 95% CI = 29.3% to 53.5% vs 96.0%, 95% CI = 92.2% to 98.0%, P < 0.001). However, in mutation carriers aged 30–39 years, the combination of MRI and mammography had comparable sensitivity to MRI alone (100.0% vs 96.8%, 95% CI = 79.2% to 100.0%, P = 0.99), but with a statistically significant decrease in specificity (78.0%, 95% CI = 74.7% to 80.9% vs 86.2%, 95% CI = 83.5% to 88.5%, P < 0.001). For women aged 50–69 years, combining MRI and mammography has significantly increased sensitivity compared to MRI alone (96.3%, 95% CI = 90.6% to 98.6% vs 90.9%, 95% CI = 83.6% to 95.1%, P = 0.02), with a small but statistically significant decrease in specificity (84.2%, 95% CI = 83.1% to 85.2% vs 90.0%, 95% CI = 89.2% to 90.9%, P < 0.001). Annual MRI as the primary screening modality for detecting cancer in high-risk women aged 30–39 years, especially those who are mutation carriers. For women aged 50–69, the most effective approach for cancer detection involves combining annual mammography with MRI screening. In fact, this approach allows us to detect the small-size tumour, determine the location and extent of the tumour as well as the involvement of nearby lymph nodes and potential dissemination to adjacent tissues [93].

### Surface-enhanced Raman spectroscopy (SERS)

SERS is a powerful analytical technique that can be applied in various fields, such as biochemical and bimolecular diagnosis, including breast cancer treatment. SERS enhances the Raman scattering signal from molecules adsorbed on or near specially designed nanostructured surfaces. One prominent example is SERS, which shows excellent potential for genomic and proteomic research. SERS-based DNA labeling systems enhance the effectiveness of gene-probe technology in gene diagnostics. In fact, Raman imaging also holds significant promise in breast cancer diagnosis due to its unparalleled sensitivity, multiplexing capabilities, and biochemical selectivity and specificity for molecular imaging [94]. SERS is believed to be used to monitor the response of cancer cells to various treatment modalities, such as chemotherapy or radiation therapy. This could be achieved by labeling the cancer cells with SERS-active nanoparticles, which will allow us to track the changes in the molecular composition of the cells over time. This information will provide deep insights into the efficacy of the treatment and help optimize therapeutic strategies [95]. A study has been reported that employed the surface-enhanced Raman spectroscopy (SERS) technique for amplifying the Raman spectroscopy (RS) signal of serum using a composite substrate composed of high-sensitivity thermally annealed silver nanoparticles (AgNPs) and a porous silicon Bragg mirror (PSB). The result obtained from the study unveiled that SERS exhibits superior capability over RS by providing increased and more pronounced spectral peak information, thereby facilitating the identification of potential novel biomarkers associated with breast cancer [96].

### Biopsy

**Fig. 6:**
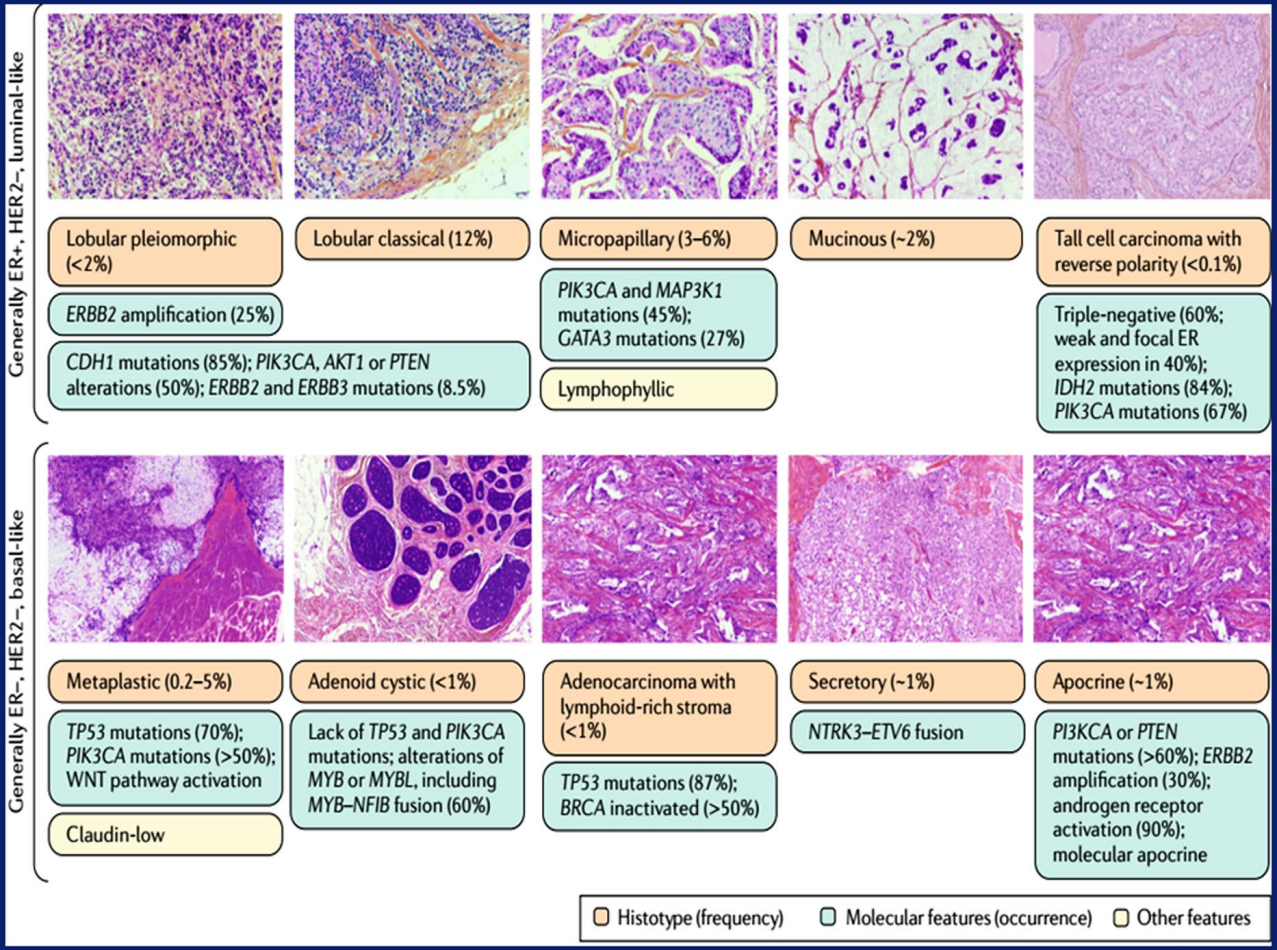
Breast cancer histological types and molecular alterations, The WHO classification distinguishes various invasive breast cancer subtypes that have particular molecular changes, some of which are shown here [98]. For instance, CDH1 mutations are present in 85% of instances of lobular carcinomas and their progenitors (lobular neoplasia), which results in the pathognomonic decrease of E-Cadherin expression [99], [100]. Additionally, they have copy number increases in ESR1, PIK3CA, PTEN, AKT1, ERBB2 and ERBB3 mutations, and PTEN. Secretory carcinomas have a particular translocation, t(12;15), which results in a gene fusion. NTRK3–ETV6 [101]. whereas t(6;9) and the fusion gene MYB-NFIB32 define adenoid cystic carcinoma [102]. Understanding these characteristics may aid in the creation of treatments that are specifically suited for certain histological subtypes [99]. Oestrogen receptor (ER), human epidermal growth factor receptor (HER2), and tall cell carcinoma with reverse polarity were identified by G. MacGrogan of the Institut Bergognié in France. (Remove the reference in the legend and cite the article from where the image has been taken)

Biopsy is a procedure used to obtain tissue samples from a suspect area of the breast for additional analysis under the microscope. In the case of breast cancer, the biopsy is essential for confirming the presence of cancer cells and determining their characteristics. Fine-needle aspiration, core needle biopsy, and surgical biopsy are a few commonly used biopsy techniques. The choice of biopsy technique depends on various factors like the size and location of the cancer cell. Figure 6 represents the histological stages and molecular alterations presented by Breast Cancer. During the biopsy, a small amount of tissue is removed under local anesthesia, and a pathologist sends the sample to a pathology laboratory for analysis. The pathologist examines the tissue under a microscope, looking for cancer cells and assessing their type, grade, hormone receptor status, and HER2 status. The biopsy results provide crucial information for accurate diagnosis, staging, and treatment planning in breast cancer patients [97].

Staging is essential during breast cancer evaluation as it allows for determining the extent of the spread of cancer. The TNM system is commonly used for staging breast cancer, considering the tumour size (T), lymph node involvement (N), and the presence of distant metastasis (M). Furthermore, additional factors like tumour grade, hormone receptor status, and HER2 status also influence the stage and help in taking a decisive decision for treatment [103].

## Treatment/management of breast cancer

The WHO has launched a Global Breast Cancer Initiative Framework with the aim of saving 2.5 million lives from breast cancer by 2040. This initiative emphasizes three key strategies: promoting early detection through health campaigns, ensuring timely diagnosis, and providing comprehensive management of breast cancer. It urges all nations to adopt these strategies to achieve the goals set forth in the initiative [104]. In the case of early breast cancer, neo-adjuvant treatment is often recommended. Neo-adjuvant therapy, which encompasses targeted therapy, hormone therapy, or chemotherapy, is administered before surgery. The purpose of neo-adjuvant therapy is to shrink the tumor, improve surgical outcomes, and increase the possibility of breast-conserving surgery (lumpectomy) rather than a mastectomy [105]. Locoregional therapy is another approach for early breast cancer, targeting the tumour and surrounding lymph nodes. Typically, radiation therapy is administered after surgery to eliminate any remaining cancer cells and minimize the risk of spreading of cancer to other parts of the body [106]. The combination of breast-conserving surgery and radiation therapy has shown comparable recurrence rates compared to mastectomy, although the specific recurrence rates may vary depending on the subtype of BC [107]. Radiation therapy itself plays a crucial role in managing early-stage breast cancer. It employs high-energy X-rays or other radiation sources to target and destroy cancer cells in the breast and adjacent tissues, aiming to minimize the risk of recurrence in those areas. Advances in radiation therapy techniques, such as hypo-fractionated whole-breast irradiation and stereotactic body radiation therapy, have improved treatment outcomes and may continue to evolve in the future [108]. Chemotherapy is a common treatment option for invasive or advanced breast cancer. It involves administering powerful drugs orally or through injection to target and inhibit the growth of aggressive cancer cells [109]. Neo-adjuvant and adjuvant chemotherapy are used depending on the risk of recurrence, and the optimal schedule typically includes taxane-based therapy with or without anthracyclines. Dose-intensified chemotherapy has shown superiority over conventional-dose therapy in some cases [110].

Hormonal therapy is a treatment approach specifically used for breast tumours that are positive to hormone receptors. It works by blocking the effects of estrogen or progesterone, which stimulate the growth of cancer cells. Treatment options include tamoxifen or aromatase inhibitors like exemestane, anastrozole and letrozole. Aromatase inhibitors have shown a greater risk to reduce the recurrence compared to tamoxifen, and an extended adjuvant therapy for up to 10 years has been investigated for high-risk patients [111].

## Treatment of advanced breast cancer

Metastatic breast cancer, also referred to as advanced breast cancer, occurs when cancer cells spread beyond the breast tissues and adjacent lymph nodes to other parts of the body. It is currently treatable but not curable, and most patients eventually succumb to the disease. The survival rate for women with advanced breast cancer is approximately 2–3 years [112].

Treatment for advanced breast cancer typically involves a combination of therapies, including radiation therapy, targeted therapy, chemotherapy, hormone therapy, and surgery. The objective of treatment is to control the tumor, manage symptoms, and improve the quality of life. There are two types of BC that can lead to distant metastasis. De novo metastatic BC refers to the condition where BC has already spread to adjacent organs at the time of initial diagnosis. Recurrent BC, on the other hand, occurs when cancer returns or spreads to other parts of the body after the successful treatment for early-stage BC. Both types require appropriate and personalized treatment to lower the risk of death from metastatic BC. Recurrent BC is typically more resistant to treatment and is more aggressive, while de novo metastatic disease presents challenges in addressing the benign tumor [113]. Although metastatic BC is generally considered incurable but progressive development in treatment options have increased survival rates and improved life quality for women with the disease. For instance, combining chemotherapy with liver resection for liver metastases from breast cancer has resulted in 5-year survival rates exceeding 40% in some cases. However, surgical management for metastases is currently limited to carefully selected patients treated at specialized high-volume centers [114].

Hormone receptor-positive breast tumor therapy involves blocking estrogen or progesterone effects to inhibit uncontrolled cancer cell growth. Aromatase inhibitors are used when front-line or sequential treatments have shown a greater reduction in the absolute risk of recurrence at 10 years compared to tamoxifen. These inhibitors have been found to be effective in reducing the recurrence incidence and mortality in postmenopausal females with ER-positive early BC [111]. Moreover, radiation therapy plays a decisive role in managing symptoms associated with metastases in various organs like bone, the brain, and soft tissue. It can aid in alleviating pain, neurological symptoms, and impede cancer proliferation to other parts of the body.

Conventional approaches that are not effective in treating primary tumors are now also under investigation for improvement. Radiation therapy has shown potential in eliciting immune responses that can act on non-irradiated tumor cells adjacent to or distant from the irradiated site [115]. Thus, it is essential to thoroughly study, monitor, and manage these side effects, particularly in patients with metastatic BC. For instance, chemotherapy, a common treatment approach can cause different side-effects like vomiting, nausea, fatigue, early menopause, mouth sores, cardiotoxicity, infertility, susceptibility to infections, neuropathy and cognitive dysfunction [116]. Medications, such as carvedilol and duloxetine, have shown efficacy in mitigating heart function reduction and treating chemotherapy-induced peripheral neuropathy, respectively [117]. Endocrine therapy, such as tamoxifen, can cause side effects like hot flashes. Ovarian suppression-based regimens can be effective but often lead to severe hot flashes. Managing hot flashes may involve the use of medications like venlafaxine and gabapentin, as well as mental techniques like hypnosis and acupuncture. Aromatase inhibitors commonly cause side effects, such as vaginal dryness and dyspareunia [118]. Radiation therapy, while an important treatment for breast cancer, also has side effects, including lymphedema. Other side effects after breast cancer surgery include pain, numbness, limited range of motion, fatigue, scarring, and emotional impact. Radiation therapy can also lead to dermatitis, pneumonitis, acute fatigue, chronic pain, and cosmetic issues, affecting the patient's quality of life [119].

In addition to physical side effects, breast cancer treatments impose financial and psychosocial burdens on patients. These can include job loss, care costs, emotional stress, and dependence on others for support. As conventional treatments become less effective in advanced stages, there is a need for new, more targeted and effective treatment options for breast cancer patients.

## Nanomaterials: an alternative solution

**Fig. 7:**
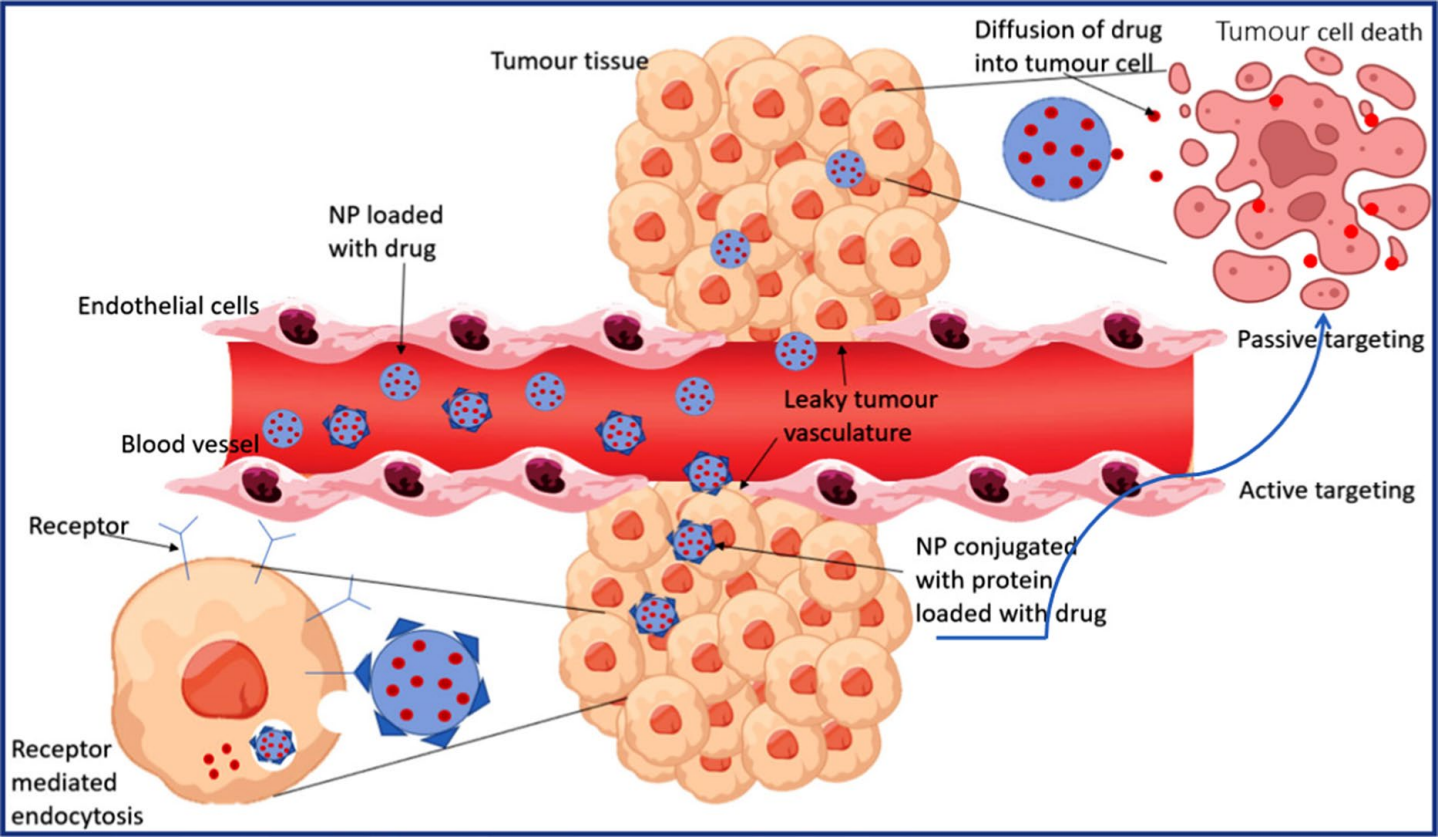
Illustrates a comparison between passive and active targeting of drugs for breast cancer treatment [123]

Nanomaterials have emerged as a potential new approach for treating BC that could reduce these side effects while drastically increasing the effectiveness of treatment. Owing to the challenges associated with the conventional treatment methods, the utilization of nanomaterials has gain significant interest and emerged as promising tool for therapy [120]. Nanomaterials refer to materials with one dimension (at least) measuring below 100 nm. Multimodal synergistic therapy involves combining multiple therapies onto a single platform, which has been made possible by advancements in nanotechnology in the biomedical field. Nanoparticles (NPs) have advantages over other materials, as they can quickly integrate with multiple therapies through adsorption and binding forces. Nanoparticles can be engineered to target tumor sites actively or passively, offering precise delivery of therapeutic agents while minimizing side effects shown in Fig. 7.

### Passive targeting

#### Enhanced permeability and retention (EPR) effect

Tumors have leaky blood vessels with larger gaps (fenestrations) in their endothelial linings compared to healthy tissues. Nanomaterials, particularly those with sizes ranging from 10 to 200 nm, can exploit this by circulating in the bloodstream and passively extravasating through these leaky blood vessels into the tumor interstitium. Nanomaterials can be tailored in size and surface properties to exploit the EPR effect. Smaller nanoparticles tend to extravasate more efficiently, while surface modifications such as polyethylene glycol (PEG) can prolong circulation time and reduce recognition by the reticuloendothelial system (RES), enhancing accumulation in tumors [121].

### Active targeting

Nanomaterials can be functionalized with ligands such as antibodies, peptides, aptamers, or small molecules that specifically recognize and bind to receptors or antigens overexpressed on the surface of cancer cells. This active targeting enhances the specificity and efficiency of nanoparticle accumulation at the tumor site. These ligands can target various molecules, including receptors for growth factors or angiogenesis-related proteins, which are often up-regulated in cancer cells and tumor vasculature. By exploiting these molecular signatures, nanomaterials can be guided to the tumor site with high specificity shown in Fig. 7. Upon binding to their targets, nanomaterials can be internalized by cancer cells through receptor-mediated endocytosis, delivering therapeutic payloads directly into the tumor cells [122].

## Synthesis of nanomaterials

Nanomaterials possess distinctive characteristics that render them suitable for various applications in the field of nanomedicine. They can be classified based on their structure, ranging from zero-dimensional (0D) to three-dimensional (3D) nanomaterials. Different types of nanomaterials include Quantum Dots (0D), nanorods, nanowires (1D), nanosheets (2D), and nanocubes (3D). The synthesis of nanoparticles involves creating particles with precise characteristics like composition, shape, size and structure for diverse applications in catalysis, the environment, electronic, and medical therapies [124].

There are two main approaches to synthesizing nanomaterials:

1. Top-Down Approach: This method involves reducing bulk materials into smaller particles through physical or chemical means. It typically uses mechanical methods like milling or grinding to break down the parent material into nanoparticles. Physical methods like high-energy milling, vapour condensation, sputtering, lithography, and laser ablation are commonly used in this approach. It is often utilized for the production of metal nanoparticles like copper, gold, and silver [124].
2. Bottom-Up Approach: This method involves building nanoparticles from smaller units, such as atoms or molecules. The process starts with a solution containing precursor molecules or atoms, which undergo chemical reactions to form nanoparticles. The bottom-up approach offers precise control over the size, shape, and composition of the nanoparticles [125]. It includes methods like chemical vapor deposition, sono-chemical synthesis, hydrothermal synthesis, electrical deposition, sol–gel method, co-precipitation, cluster assembly, self-assembly, and atomic layer deposition. Biological approaches can also be considered as part of the bottom-up approach.

These synthesis methods enable the production of nanomaterials with specific properties, facilitating their application in various fields.

## Nanoparticle-loaded/modified nanomaterials for treating breast cancer

In the last decade, nanomedicine has emerged as a promising treatment for difficult–to–treat maladies such as BC. NPs possess distinct properties like an improved permeability and retention (EFR) effect and nano-size, which enable delivery of anti-cancer agents to targeted tumorous tissues and facilitate controlled release of drugs at specified sites [125]. Drug delivery systems based on nanomaterials offers various advantages for BC compared to traditional delivery systems. These include reduced side-effects, prolonged circulation half-life, enhanced therapeutic effects, improved permeability and patient compliance [126]. The two nanomaterials that are extensively used for targeted and non-targeted delivery of chemotherapeutic agents are organic NPs (such as dendrimers, exosomes liposomes, micelles and polymers) and solid lipid NPs (carbon-based).

### Liposomes

Liposomes are spherical particles composed of a phospholipid bilayer that can encapsulate drugs. For instance, a liposomal formulation named “Doxil” contains doxorubicin (DOX) within it that has been used as a treatment option for BC [127]. DOX is a “small anthracyclin molecule” which effectively fights rapidly dividing cancer cells but can become toxic if given repeatedly. Therefore, its dosage has been limited to 450–550 mg/m^2^ to prevent risks of cardiotoxicity [127]. Moreover, PEGylation of DOX prevents the uptake by the phagocyte system of mononuclear cells, resulting in prolonged drug release with a half-life of 3–4 days [128]. Moreover, Doxil and DOX are fluorescent at a wavelength of 479/559 nm, which allows for the monitoring of the transport and delivery of drugs through live-cell fluorescence imaging [129].

In another study, researchers aimed to improve the treatment outcomes for TNBC, which is known to be aggressive and resistant to many cancer therapies, even targeted therapy. They explored a novel approach using macrophages, which can actively target tumor sites, as carriers for chemotherapeutic drugs. By attaching DOX-carrying liposomes to the surface of macrophages using biotin-avidin interactions, they created a macrophage-liposome (MA-Lip) system [130]. Porous silicon nanoparticles (PSiNPs) can also be used for targeted cancer chemotherapy, as it mimics exosomes and is biocompatible with tumor cells. To produce exosome-sheathed DOX-laden PSiNPs (DOX@E-PSiNPs), DOX can be added to the PSiNPs before they are loaded with exosomes and exocytosed from tumor cells. When given intravenously, these particles have demonstrated a better ability to reach deeper regions of tumor tissue, leading to increased DOX accumulation at the targeted tumor site [131]. This drug delivery system can also extravasate from blood arteries, improving drug delivery to malignant cells.

### Polymeric nanoparticles

Another type of drug delivery system includes polymeric NPs, made up of biodegradable polymers and can be used to encapsulate hydrophobic and hydrophilic drugs. These NPs improve drug solubility and stability and have been designed to target specific BC cells. Abraxane is another U.S. Food and Drug Administration-approved drug delivery system for treating metastatic breast and lung cancer. Abraxane is composed of albumin-bound NPs of paclitaxel, with a 130 nm size, which upon evaluation, has shown superior efficiency and reduced toxicity of ABI-007 in contrast to standard paclitaxel [132]. The albumin protein serves as a natural carrier for paclitaxel, which aids in targeted drug delivery and reduces toxicity and has been found to have lower plasma concentrations than Taxol when assessed using both human and animal models. Research has shown that Abraxane eliminates cancer stem cells (CSC) but has similar cytotoxicity to Taxol in SUM149 cells [133].

### Polymeric micelle

A polymeric micelle is a nanoscale structure composed of amphiphilic (having both hydrophilic and hydrophobic properties) block copolymers. These block copolymers consist of two or more different types of polymer chains linked together. Genexol-PM is a Taxane-based drug delivery system formulated as a polymeric micelle to improve drug solubility and targeted delivery to BC cells. The active ingredient in Genexol-Pm is paclitaxel, which restricts the division and growth of BC cells. A recent study reported that stereo-complex PEG-PLA micelles fabricated by researchers with the help of stereo-selective interactions of enantiomeric PLA, improved structural stability and drug loading capacity. These micelles act as an effective drug delivery system for targeting tumor cells. In order to improve the therapeutic outcomes, a hypoxia-responsive moiety has been incorporated as a hypoxia-cleavable linker. This innovative design allows the micelles as Genexol-pm to respond sensitively to the hypoxic tumor environment, facilitating controlled drug release. The goal of this approach was to reverse hypoxia-induced cell migration and drug resistance, hence increasing bioavailability under hypoxic conditions [134].

### Dendrimers

Dendrimers are intricately branched polymers resembling trees, possessing a precisely controlled and well-defined structure. They offer the ability to incorporate targeting ligands or encapsulate drugs to treat cancer. In BC therapy, dendrimers have emerged as a promising tool due to their ability to improve the delivery of chemotherapy drugs and enhance their therapeutic efficiency. Research analysis reported that dendrimers derived from poly (amido amine), namely G1(Phe)6, G1(Dan)3, and G2, were investigated for their potential to incite cell death in a BC (MCF-7, MDA-MB-231) cell line and leukemia cell line. The results showed that the dendrimers had a concentration-dependent effect on mammary cancer cells [135]. Another research group intended to augment the transportation of docetaxel (DTX) to HER2-positive BC cells by synthesizing dendrimers grafted with trastuzumab (TZ). To achieve this, researchers employed a hetero-crosslinker called MAL-PEG-NHS to bioconjugate TZ onto the surface of dendrimers. Additionally, fluorescein isothiocyanate was also attached to the dendrimers to enable imaging of cancer cells and in vitro analysis demonstrated that the specific dendrimers were more selective and exhibited higher anti-proliferation activity against HER2-positive MDA-MB-453 than a HER2-negative MDA-MB-231 cell line [136].

### Carbon dots

Carbon dots (CD) are additional promising nanomaterials for various medical applications, including BC treatment. CD are fluorescent carbon NPs with numerous advantages like easy amendment, biocompatibility, and low toxicity [137]. Research analysis has highlighted the potential of CD derived from N-hydroxy phthalimide (CD-NHF) in modulating tumor cell movement and invasion in both in vivo and in vitro conditions, thereby influencing cancer progression. The study found that CD-NHF significantly hindered the movement and invasion of metastatic BC cells, effectively impeding the malignant phenotype. These findings were further supported by the down-regulation of cellular markers, like Ki65 and HSP90, in a synergistic model. The accorded outcomes highlight the significant role of CD-NHF in suppressing the invasive characteristics of BC cells and suggest its potential utility as a therapeutic intervention for managing cancer progression [138]. Another study reported the loading of Gemcitabine onto nitrogen-doped CQD NPs via electrostatic interactions and was used for evaluating the viability of MCF7 cells. In vivo imaging and bio-distribution studies on BC cells growing in mice were also conducted. The results obtained from the study indicated that these N-CQDs exhibited exceptional luminescent properties and demonstrated high accumulation specifically in tumor tissues. These findings suggest that Quinic acid-conjugated N-CQDs hold great promise as an effective candidate for multifunctional theranostic purposes [139].

Another study examined the latest developments in carbon nanotubes, particularly conjugated single-walled carbon nanotubes (SWNT), and their potential to improve BC treatment. These nanotubes demonstrated promising attributes such as cancer-specific accumulation, biocompatibility, easy elimination from the body, and low toxicity [140]. Research investigation regarding CNTs and BC involved studying their potential cytotoxic effects on MC4L2 cells and mice analyzing their influence on the ablation of breast tumors [141].

### Inorganic nanocarriers

Inorganic nanoplatforms composed of metal/metal oxide NPs like gold (Au), silver (Ag), nickel (Ni), cobalt (Co), copper (Cu), silicon dioxide (silica), iron oxide (Fe_2_O_3_, Fe_2_O_3_, Fe_3_O_4_), nickel oxide (NiO), titanium dioxide (TiO_2_), copper oxide (CuO), and semiconductors (like cadmium sulfide (CdS)), offer added advantages such as high quantum yields, modifications that not only enable controlled drug release but also exhibit low toxicity, enhanced bioavailability, and superior drug loading capacity. Moreover, modified surfaces, compared to their organic counterparts, showcase remarkable attributes such as high photo-stability and a significantly larger surface area, further bolstering their utility in the biomedical field. Carbon nanotubes, graphene, metal–organic and upconversion nanocrystals are a few other inorganic NPs that are currently under investigation. One approach being investigated involves the uses of ligand shells or organic components on functionalized inorganic NPs, with the improvement of their colloidal stability and amendment of surface chemistry. This enables the selective eradication of cancer cells in BC treatment while minimizing harm to healthy cells, making them an appealing approach for therapy [142].

**Fig. 8:**
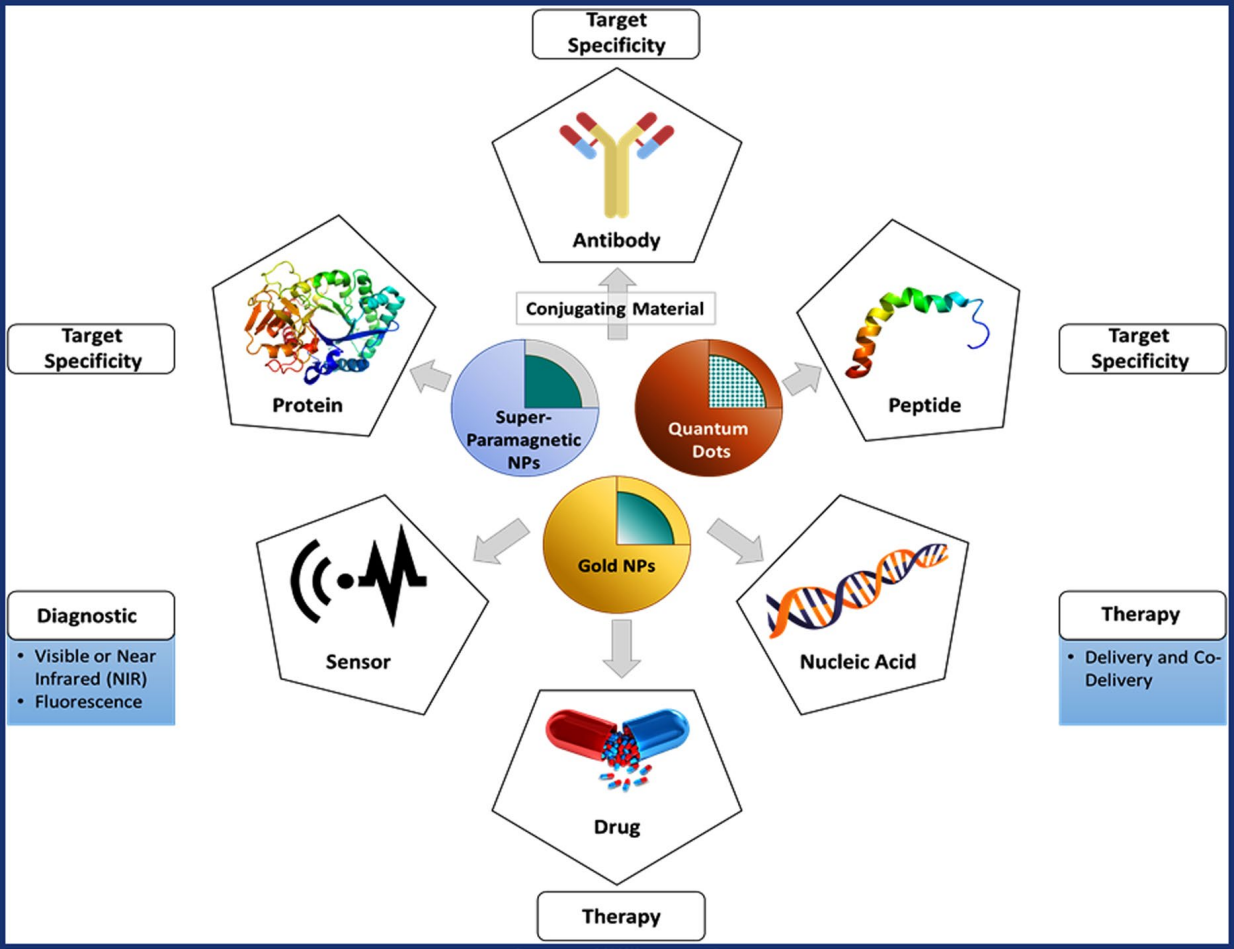
Graphical demonstration of inorganic nanoparticles along with their conjugating materials and their potential applications

AuNPs have emerged as a promising candidate for BC treatment and these NPs can easily be synthesized in various size and shapes like shells, rods and spheres, to meet commercial demand. Usually, Au-NPs are relatively inert and can be easily modified with various surface chemistries, expanding their potential for functionalization. Furthermore, the unique tunable optical and thermal characteristics of Au-NPs, attributed to surface plasmon resonance (SPR), make them suitable for cell ablation applications. Gold and other inorganic NPs can be functionalized and used as delivery vehicles for treating and diagnosing BC, as elucidated in Fig. 8. [143] .

Au-NPs synthesized within micellar networks formed by an amphiphilic block copolymer have emerged as effective nanocarriers for targeted drug delivery. These Au-NPs offer advantages like immunogenicity and low cytotoxicity. The nanocarrier, called Au-NM, combines the unique properties of an inorganic gold material with a poly(ethylene glycol)-block-poly(propylene glycol)-block-poly(ethylene glycol) (PEG–PPG–PEG) amphiphilic polymer. To improve the functionality of Au-NM, it was modified by attaching the dual tyrosine kinase inhibitor ZD6474 (FDA-approved), resulting in a conjugate called ZD6474–Au-NM. The physicochemical properties of ZD6474–Au-NM were investigated, revealing a stable structure with a diameter of approximately 70 nm under physiological pH conditions. In acidic conditions (pH 5.2), ZD6474 exhibited a gradual-release pattern from Au-NM, enabling controlled drug release in acidic tumor environments. In vitro experiments using TNBC cells demonstrated that ZD6474–Au-NM significantly reduced their proliferation rate, migration and invasion, while inducing programmed cell-death [144].

A novel approach has been adopted to concurrently target mitochondrial DNA (Mt-DNA) and nuclear DNA (n-DNA) utilizing a targeted theranostic nanodelivery vehicle (TTNDV). In this study, folic acid-anchored p-sulfo-calix[4]arene (SC4)-capped hollow gold nanoparticles (HGNPs) were meticulously loaded with antineoplastic doxorubicin (Dox) and its mitochondrion-targeted analogue, Mt-Dox, in a pre-defined ratio (1:100) to enable sustained thermoresponsive release of cargo [145]. A modified form of tamoxifen, a thiol-PEGylated tamoxifen derivative, was developed to specifically target and deliver plasmonic Au-NPs to BC cells expressing estrogen receptors. This modified tamoxifen showed increased drug potency up to 2.7 times greater than the original tamoxifen when tested in laboratory conditions. The increased efficacy of tamoxifen when delivered using nanoparticles is attributed to its enhanced intracellular transport through nanoparticle endocytosis [146]. The presence of estrogen receptor alpha on the cancer cell membrane is believed to facilitate the selective uptake and retention of these therapeutic nanoparticle conjugates. This dual targeting specificity and improved potency offer opportunities for the development of multimodal endocrine treatment approaches and the potential use of adjunctive laser photothermal therapy [147].

In another study, researchers developed a drug delivery system (DDS) called Au–Se@MSN, which utilizes silica mesoporous NPs (MSN) to deliver resveratrol effectively. To achieve controlled drug release and prevent premature release, selenol-modified uPA-specific peptides are employed as gatekeepers on Au-NPs. These gatekeepers effectively hinder interference from glutathione present in the bloodstream. The Au–Se@MSN(RES) system exhibits sustained release of resveratrol at the tumor site and demonstrates a significant therapeutic effect in in vitro conditions. Intriguingly, the administration of pharmacological doses of resveratrol MDA-MB-231 cells resulted in the generation of increased levels of NAD(P)H instead of H_2_O_2_. This suggests that reductive stress, rather than oxidative stress, plays a crucial role in the therapeutic mechanism of resveratrol. In vivo studies using mice bearing TNBC tumours demonstrated that Au-Se@MSN significantly enhanced the chemotherapeutic potential of resveratrol while minimizing harm to normal tissues and cells [148].

Studies have shown that silver nanoparticles (AgNPs) can also inhibit the growth and proliferation of BC cells and can induce apoptosis in tumor cells. Exposure to AgNPs resulted in a depletion of cellular antioxidants and endoplasmic reticulum stress in TNBC cells but not in non-malignant breast epithelial cells. In a 3D cell culture, AgNPs caused extensive DNA damage in TNBC tumor nodules but did not disrupt the typical architecture of breast apoptosis. Furthermore, systemically administered AgNPs were effective at reducing TNBC tumor xenograft growth in mice at non-toxic doses. The results point that AgNPs have the ability to be a targeted and represent a safe therapeutic option for TNBC [149].

The synthesis of Au–Ag@HA NPs through the reduction of HAuCl_4_ and AgNO_3_ in the presence of trisodium citrate was followed by the alteration of its surface by hyaluronic acid (HA). The introduction of HA modification enabled the targeted binding of Au–Ag@HA nanoparticles to CD44 receptor-overexpressing 4T1 BC cells. Notably, the NPs exhibited both ionizing radiation capability and peroxidase-like activity, leading to the generation of hydroxyl radicals (•OH) and the controlled release of Ag^+^ ions (toxic) specifically at the tumor sites. Consequently, this approach led to a highly effective therapeutic outcome for treating tumors. The findings from this research demonstrated it to be a promising treatment strategy involving the combination of radiation, nano-enzymes, and the release of Ag^+^ ions for cancer therapy [150]. Albumin-coated silver NPs (ASNPs) have been synthesized and assessed for their ability to combat cancer in the MDA-MB 231 human BC cell line. The cells were examined using inverted fluorescent microscopy, which revealed notable morphological changes and DNA ladder patterns on gel electrophoresis, indicating cell death through apoptosis. The LD_50_ of ASNPs against MDA-MB 231 cells (5 μM) was determined to be 30 times greater than that for healthy white blood cells (152 μM). The synthesized ASNPs exhibited several desirable characteristics, including an intact albumin coating, enhanced cytotoxicity against cancer cells compared to normal cells, induction of apoptosis-mediated cell death, and reduced tumor size in mice [151].

To enhance the anticancer properties of silver nanoparticles (Ag NPs), a biosynthesis approach using turmeric-powder extract was employed to create turmeric-based silver nanoparticles (TuAgNPs). The TuAgNPs exhibited significant anticancer activity with EC_50_ values of 0.324 mg/mL, 0.141 mg/mL and 0.146 mg/mL, against MCF 10A, MDA-MB-231, and MCF-7 cell lines, respectively. Additionally, the TuAgNPs demonstrated a notable antioxidant activity of 77% at 0.5 mg/mL. In antibacterial tests against gram-negative strains, the TuAgNPs showed promising results with a minimum inhibitory concentration (MIC) of 6.25 µg/mL for ATCC (25,922) control and CTXM-15 negative strains, and 12.5 µg/mL for CTXM-15 positive strains. The minimum bactericidal concentration (MBC) values were 6.25 µg/mL, 25 µg/mL, and 50 µg/mL for the ATCC (25,922) control, CTXM-15 negative, and CTXM-15 positive strains, respectively. These findings highlight the potential of TuAgNPs as effective agents for both anticancer and antibacterial applications [152].

Copper is a naturally occurring trace element imperative in various biological processes, including angiogenesis, apoptosis and cell proliferation. The use of chitosan-copper sulfide nanocomposites for the controlled release of the therapeutic from mesoporous silica have potential applications in both therapy and diagnosis for BC [153]. Recent research explored the use of polymeric copper chelator–based NPs for metastatic BC [154]. However, it is important to note that cobalt nanomaterials are effective against BC cells. Cobalt spinel ferrite NPs (CFO) and bismuth-doped cobalt spinel ferrite NPs (CBFO) were synthesized using an auto-combustion method with glycine as the fuel agent. The biocompatibility of the NPs was evaluated on human BC cells (T47-D and MDA-MB-231) and prostate cancer cells (PC3). CBFO nanoparticles showed higher efficacy in inducing cancer cell death, with IC_50_ values of 134.38 µM, 66.41 µM and 61.12 µM for PC3, T47-D and MDA-MB-231cancer cells, respectively. Furthermore, the cytotoxicity of CBFO NPs towards normal human cells was minimal, with a percentage lower than 2% even at high particle concentrations. Therefore, CFO and CBFO compounds are promising therapeutic agents against cancer cells [155]. In another study, graphene oxide (GO)/cobalt ferrite NPs were utilized for heat treatment of the MCF7 cell line; in vitro experiments revealed that after 72 h of nanoparticle treatment, it resulted in an IC_50_ value of cell survival of 58%. Hyperthermia reduced cell viability to 30%. In vivo, the tumor size decreased with 0.001 and 0.002 g/ml concentrations and 400 and 250 kHz magnetic frequencies for 10 min. MRI confirmed NP presence in tumor and tissues after 27 days [156].

### Magnetic nanoparticles

Magnetic nanoparticles, such as Fe_3_O_4_ and γFe_2_O_3_, have been extensively studied for their potential use in BC diagnosis and treatment. The magnetic technique employs superparamagnetic iron oxide particles (SPIO) called Sienna + ®, while the radioisotope technique involves Tc99 with or without a blue dye. Analysis of data from 107 patients demonstrated that the magnetic tracer achieved a per-patient detection rate of 98.13%, whereas the standard technique achieved a rate of 92.26%. Regarding nodal detection, the magnetic tracer identified 93.07%, whereas the standard technique identified 96.53%.

Remarkably, in the subset of 31 patients with positive sentinel lymph nodes (SLNs), both techniques achieved a 100% detection rate for SLNs, successfully identifying all 31 cases. Furthermore, a separate study focused on functionalizing superparamagnetic La0.7Sr0.3MnO_3_ nanoparticles (SPMNPs) by modifying them with an oleic acid-polyethylene glycol (PEG) structure. These modified SPMNPs exhibited a high loading capacity of approximately 60.45% for the anticancer drug “DOX”, enabling efficient in vitro delivery into cancer cells. Upon loading DOX, the cancer cell death rate reached 89% within 24 h, comparable to the effectiveness of free DOX (75%).

Furthermore, by employing a strategy involving DOX-conjugated SPMNPs-induced hyperthermia, we achieved a remarkable cancer cell extinction rate of up to 80% within 30 min under in vitro conditions. In order to evaluate the in vivo performance of the micellar SPMNPs, a mice model was employed, and a dose of 50 mg/kg of SPMNPs was administered. Through biodistribution analysis, the presence of the micellar SPMNPs was observed in multiple tissues. These results collectively indicated that the developed polymeric micelle SPMNPs hold significant potential as a multifaceted strategy for effective combined chemotherapy-hyperthermia cancer therapy [157].

In vitro studies were performed using SKBR3 and T47D BC cells. These cells were exposed to superparamagnetic mesoporous hydroxyapatite nanocomposites (SPmHANs) conjugated with 1 μM DOX and 0.5 mM 2-Deoxy-d-Glucose (2DG). Subsequently, the cells were subjected to γ-ray irradiation at doses of 1 and 2 Gy. This combination treatment resulted in a significant decrease in viability of cells in contrast to radiotherapy alone. The loading efficiency of 2DG on SPmHANs was determined to be 42% for 200 ppm, while DOX had a loading efficiency of 93% for 5 ppm. The viability of the cells was found to be 60.9% and 68%, which was lower in contrast to radiotherapy alone for T47D and SKBR3 cells, respectively. Additionally, the ankyrin repeat protein (DARPin) G3 was designed as a high-affinity binding protein for HER2. Superparamagnetic nanoparticles (SPIO-G3-5MF) modified with fluorescein-5-maleimide-labelled DARP in G3 demonstrated a uniform size (≈100 nm), low cytotoxicity and good dispersity. These nanoparticles selectively bound to HER2-positive BC cells, even in the presence of trastuzumab. Biodistribution analysis confirmed substantial aggregation and prolonged retention of SPIO-G3-5MF in HER2-positive models of BC. Furthermore, MR imaging exhibited selective imaging of HER2-positive tumours with significant T2 signal reduction (50.33 ± 2.90% at 6 h and 47.29 ± 9.36% at 24 h). The findings suggest the potential of SPIO-G3-5MF as a molecular probe for monitoring and diagnosing HER2 expression in BC [158].

Polymer encapsulated super-paramagnetic NPs are effective in BC theranostics, combined with magnetic hyperthermia and controlled drug release. Superparamagnetic iron oxide nanoparticles (SPIONs) are made in situ utilizing a poly (ethylene glycol) (PEG) reactor and microwave hydrothermal process on reduced graphene oxide nanosheets (rGO). These nanocomposites possess the ability to generate localized heat when subjected to an external magnetic field, with a specific absorption rate (SAR) value of 1760 ± 97 W/g. The temperature can reach up to 43 ± 0.3 °C, resulting in substantial ablation of MCF-7 cells. This remarkable magnetothermal property is achieved through the synergistic effects of rGO and PEG functionalities, which also contribute to the improved synthesis, stability, and biocompatibility of the SPIONs [159]. In the BC mice model, the combined effect of the Fe_3_O_4_/SiO_2_-CUR nanocomposite with photodynamic therapy (PDT) and photothermal therapy (PTT) was assessed. Immunohistochemistry (IHC) analysis was done to evaluate Bax and Caspase3 protein expression. The study included six treatment groups: control, CUR + PDT, Blue + NIR lasers, NC, NC + PTT, and NC + PDT + PTT. The NC + PDT + PTT group exhibited a significant reduction in tumor volume compared to the other groups (P < 0.05). Remarkably, the tumor volume in the NC + PDT + PTT group was reduced by 27% in comparison to its initial volume. No notable losses of weight or severe effects on vital organs were recorded. IHC analysis revealed significantly higher expression of proapoptotic Bax and Caspase3 proteins in the NC + PDT + PTT group, indicating apoptosis. These findings suggest that the NC + PDT + PTT strategy holds promise as an alternative to chemotherapy for treating triple-negative breast cancers.

### Mesoporous silica nanoparticles

A study reported the development of mesoporous silica NPs tailored with monoclonal antibodies to target BC cells specifically. A novel Her-Dye@MSN nanoparticle was developed by conjugating a green fluorescent dye-loaded mesoporous silica nanoparticle (MSN) with an anti-HER2/neu monoclonal antibody (mAb). The mAb was linked to Her-Dye@MSN via a polyethylene glycol spacer. The study demonstrated the remarkable targeting efficacy of Her-Dye@MSN nanoparticles towards BC cells expressing high levels of HER2/neu [160]. Another study reported focusing on the uptake mechanisms of amino-functionalized silica nanoparticles (Si-NPs) in two cell lines: MCF-7 human breast adenocarcinoma cells and MCF-7-derived breast cancer stem cells (BCSCs). The results demonstrated that actin depolymerization significantly influenced the uptake of Si-NPs, particularly amino-functionalized Si-NPs, in MCF-7 cells. In contrast, disrupting scavenger receptors led to a stronger inhibition of amino-functionalized Si-NP uptake in BCSCs. These findings highlight the distinct endocytic mechanisms of functionalized Si-NPs in BCSCs and their potential for developing nanosized drug delivery systems with enhanced selectivity for cancer stem cell-targeted therapies [161]. Landgraf and his teammates conducted a study to address the solubility and specificity challenges of the cytotoxic compound camptothecin (CPT). To overcome these limitations, researchers loaded CPT into porous silicon nanoparticles (pSi-NP) and modified them with an antibody called cetuximab, which targets the epidermal growth factor receptor (EGFR). The primary objective of this approach was to develop a targeted nanoscale delivery system for effective cancer treatment. The study demonstrated the effectiveness of targeted camptothecin-loaded porous silicon nanoparticles (pSi-NPs) in vitro on MDA-MB-231BO cells. Upon obtaining the effective results in cell lines, these NPs were further investigated in a mouse model with humanized bone metastasis from BC [162]. Another study reported the synthesis of monodispersed and spherical mesoporous silica NPs (MSNs) with an average diameter of approximately 50 nm via the sol–gel method. These MSNs were then loaded with anticancer CDs that were synthesized hydrothermally. The resulting composite, known as MSNs-CDs, was further modified by functionalizing it with chitosan and targeting it with an anti-MUC1 aptamer. The functionalization process involved the utilization of a glutaraldehyde cross-linker. The study demonstrated strong and specific anticancer activity of functionalized mesoporous silica nanoparticles conjugated with carbon dots (MSNs-CDs) against MCF-7 and MDA-MB-231 cancer cells. Treatment with 100 μg/mL of functionalized MSNs-CDs for 48 h resulted in significant cell mortality, with 66% for MCF-7 cells and 71.8% for MDA-MB-231 cells. This indicates a substantial reduction in cell viability and suggests that the functionalized MSNs-CDs had a considerable anticancer effect on these cell lines [163].

The utilization of nickel, cobalt, and copper nanoparticles was investigated for their potential in BC treatment and targeted drug delivery. A study investigated the interaction between Ni/NiO@LAA_PEG NPs with an average size range of 75–80 nm and MCF-7 breast cancer cell lines. The study aimed to assess the cyto-genotoxicity of nickel, cobalt, and copper nanoparticles using MTT analysis and compared their efficacy with the standard drug “DOX”. Furthermore, theoretical simulations using Gaussian-09@Autodock 4.2 were performed to correlate the NP performance with the wet method of preparation by docking with PDB entries of the MCF-7 cancer cell line [164]. In another study, glutamic acid-functionalized nickel oxide nanoparticles (NiO@Glu/TSC) showed a significant growth inhibition of MCF-7 cells with an IC_50_ value of 298.33 μg/mL. No significant toxicity was accorded in HEK293 cells [165].

### Quantum dots

Quantum dots are nanoscale semiconductors particles with unique optical and electrical properties, making them the candidate of choice for various applications in biomedical research, including BC detection and treatment [166]. A novel approach was utilized to create luminescent/magnetic nanocomposite particles by combining polymer-coated γ-Fe_2_O_3_ superparamagnetic cores with CdSe/ZnS quantum dot (QD) shells. The particles consisted of a single layer of QDs attached to thiol-modified magnetic beads via thiol-metal bonds. Carboxylic groups were introduced to modify the NPs to enhance their compatibility in aqueous solutions. Anticycline E antibodies were immobilized on the nanoparticle surface to showcase their practicality, and these antibody-coated particles were utilized to target MCF-7 cells lines within the serum solutions. The selective binding of anticycline E antibodies to cycline, a BC cell surface protein, enabled effective separation. The luminescent/magnetic nanocomposite particles emitted intense luminescence, allowing easy visualization of the separated breast cells using fluorescence imaging microscopy [167].

Some studies have reported that graphene dots have potential anti-cancerous activity against BC and can be functionalized with targeting molecules that bind tumor cells. Examination of the graphene quantum dot (GQD) photophysical properties in diverse therapeutic applications, including photothermal, hyperthermic, and photodynamic therapy was conducted. This study highlights the potential of GQDs as a carrier of drugs and as a guide for combinatorial drug delivery strategies for treating breast cancer. It provides valuable insights for the scientific community to advance theranostic approaches using GQDs [168]. In a separate study, GQDs were combined with an amino porphyrin through an amide linkage using two different methods: thionyl chloride (SOCl_2_) and 1-ethyl-3-(3'-dimethyl aminopropyl) carbodiimide coupling. Under white light, these newly formed hybrids were then examined as phototherapeutic agents in high-incidence BC. The result obtained from the study showed a significant photo-cytotoxic effect at a concentration of 10 nM. Furthermore, the conjugation of GQDs to the amino porphyrin facilitated efficient uptake by the T-47D cells compared to the non-immobilized porphyrin. These findings indicated the great potential of these hybrid materials for further exploration as theragnostic agents. Furthermore, photodynamic therapy effects were also observed from graphene dots conjugated with amino porphyrins [169].

Although these physical and chemically synthesized NPs were effective in the treatment of BC, there were a few drawbacks associated with their use discussed below [170]:

- Size distribution: This method occasionally leads to irregular nanoparticle sizes, which makes it difficult to control the properties of nanomaterials.
- Cost and scalability: the cost of equipment for the top-down method is highly expensive and may not be suitable for large-scale production. Even the chemical synthesis approach is expensive due to the cost of chemicals, energy, and equipment required, thus limiting its scale-up for industrial applications.
- Material wastage: breaking down more extensive materials leads to surface defects in the synthesized NPs, such as dislocations and vacancies. These defects further affect the properties of the nanomaterials and deteriorate their performance.
- Environmental concerns: A chemical synthesis method often involves using toxic solvents and reducing agents, which can adversely affect the ecosystem.
- Risk of contamination: chemical synthesis methods can result in impurities and by-products, which can affect the quality and purity of the NPs.

## Biogenic nanoparticle synthesis methods

**Fig. 9:**
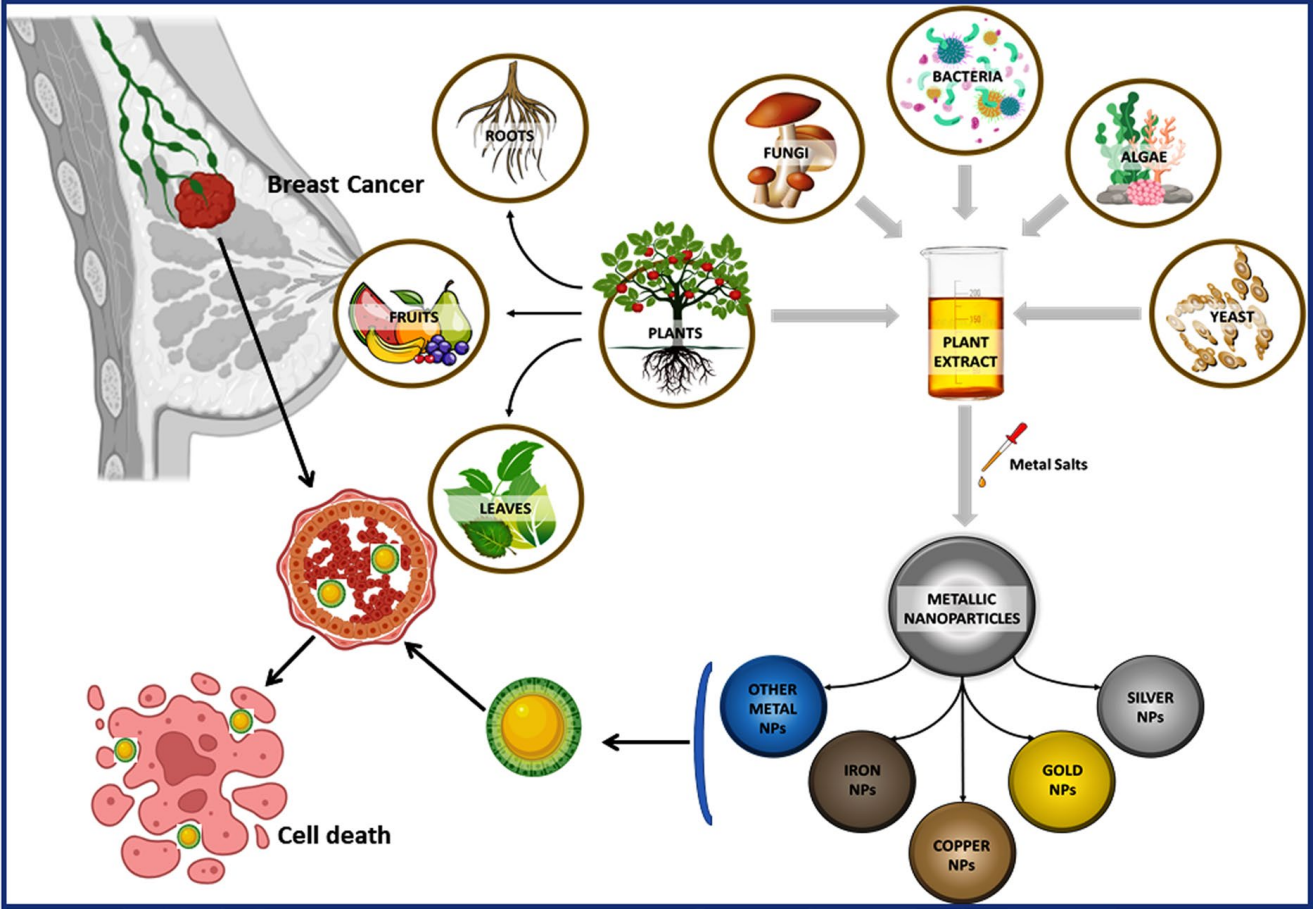
Outlines an overview of review articles focusing on the green synthesis of various metallic nanoparticles using different biological agents, along with their potential breast cancer activity

Scientists and engineers have long recognized the value of seeking inspiration from nature. This practice remains ongoing as they endeavor to replicate and surpass the remarkable properties exhibited by biological systems. In biomaterials, there are two primary methods: one involves using natural molecules and possesses that control for the formation of materials. At the same time, the other approach aims to replicate the structure and function of biogenic materials through chemistry and fabrication techniques. Biogenic NPs, also known as biosynthesized or bio-inspired or conjugated NPs, are synthesized with the help of biological systems like microbes, algae, plants, fungi and phytochemicals, as illustrated in Fig. 9. [171]

*Agents* The biological approach offers enormous advantages over physical and chemical nanomaterial synthesis methods. A few advantages of the biological synthesis of NPs have been stated below:

*Eco-friendly* Biogenic NPs are environmental-friendly and sustainable since they do not need harmful chemicals or high temperatures.

*Cost-effectiveness* Biogenic NPs can be synthesized using readily available sources such as plant and microbial extracts that makes it lower in cost than chemical and physical methods.

*Biocompatibility* Bio-inspired NPs are biocompatibility and can be employed for various biological applications like imaging, tissue engineering and drug delivery.

*Size and shape control* Biogenic nanoparticle synthesis offers excellent control on morphological parameters like size and shape of NPs.

### Microbial synthesis of biogenic nanoparticles

The microbial synthesis of NPs involves the use of microorganisms such as bacteria, yeast and fungi. Since microbes are known to possess enzymes such as reductases, which convert metal ions into metal NPs. While biomolecules such as amino acids, carbohydrates or enzymes are reducing agents. Moreover, the bacterial cell wall contains a high concentration of functional groups such as carboxyl, amino, and thiol groups. This method is relatively easy and involves culturing microorganisms in a suitable growth medium supplemented with a metal precursor. A study reported about *Bacillus cereus* obtained from contaminated soil was used for the synthesis of silver NPs. In this study, response surface methodology along with central composite design was used for optimizing different experimental factors. The antimicrobial efficacy of the synthesized NPs was evaluated against MDR microorganisms via a disc diffusion method. The MIC and minimum lethal concentrations (MCL) were determined, and the scavenging ability of the NPs against free radicals was assessed using the DPPH assay [172, 173].

Another study reported that a pretentious pigment called phycocyanin extracted from *Nostoc linckia* acted as a reducing agent in the biogenic synthesis of Ag NPs. The biogenic Ag NPs demonstrated potent inhibitory effects against pathogens including gram-positive and gram-negative bacteria species. Additionally, the Ag NPs exhibited noteworthy cytotoxic activity against MCF-7 cells, with an inhibitory concentration of 27.79 μg/ml [174]. Another study was conducted, which aimed to optimize the production of biosynthetic zinc oxide NPs (ZnO NPs) through the identification of a suitable precursor and the establishment of effective cellular compartmentalization. The endophytic strain *Streptomyces albus* E56 was cultured in an optimized medium using various techniques to improve the production of biosynthesized ZnO NPs. The effect of these NPs on cell growth was assessed by treating normal (HFB-4) cells and tumor cell lines (A549, Caco-2, HepG-2, and MDA) at different concentrations of ZnO NPs (ranging from 0–200 μg/mL) for 24 and 48 h. Furthermore, the antimicrobial and anticancer potential of the controllable biosynthetic ZnO NPs were investigated in in vitro conditions by assessing their effects at various dosages [175]. In another study, *Aspergillus terreus* was used to synthesize a nano-biocomposite of ZnO NPs combined with L-asparaginase. Further, the anti-cancer potential of this nano-biocomposite was investigated on the MCF-7 cell line via the MTT assay. The results obtained from this study showed that cell viability decreased to 35.02% in MCF-7 cells when treated with the L-asparaginase conjugated zinc oxide nano-biocomposite [176]. A study reported the utilization of biomass of *Alternaria solani,* a fungi type, for synthesizing biocompatible titanium dioxide (TiO_2_) NPs. The results obtained from the study reported inhibitory effects on gram-positive which were more distinct than gram-negative bacterial strains. Furthermore, the synthesized NPs exhibited notable cytotoxicity against human lung cancer cell lines (A549) in contrast to human BC cell lines (MCF-7) [177].

The bacterium *Stenotrophomonas malophilia* has been discovered as a novel living factory for Au-NP synthesis. In this study, the synthesis process involved the use of a particular NADPH-dependent enzyme present in the strain, which facilitated the reduction of Au^3+^ to Au^0^ by shuttling electrons. This environmentally friendly method of gold nanoparticle biosynthesis offered a straightforward, economically feasible, and sustainable approach [178]. A recent study demonstrated a fungal-mediated biosynthesis as well as stabilization of Au and Au–Ag alloy NPs. The obtained Au–Ag NPs were then subjected to different analytical techniques for characterization [179].

Another study reported that selenium nanorods synthesized by *Streptomyces bikiniensis* strainEss_amA-1 exhibited significant anticancer activity. This biosynthesized selenium nanoparticle was reported to cause the death of Hep-G2 and MCF-7 human cancer cells. The LD_5_ value of these biogenic NPs for Hep-G2 cells was 75 μg/ml, while for MCF-7 cells, it was 61.86 μg/ml [180]. Baskar et al. reported the characterization of *Bacillus cereus* strain HMH1-developed magnetic iron oxide (Fe_3_O_4_) NPs through extracellular biosynthesis and was found to impede the growth of 3T3 and MCF-7 cell lines [176]. A study demonstrated the biosynthesis of functionalized CuFe-hybrid nanocomposites (FCFNCs) using *Streptomyces cyaneofuscatus*. The FCFNCs exhibited strong anticancer properties, inducing significant apoptotic effects (> 77.7%) on Caco-2, HepG-2, MCF-7, and PC-3 cancer cells, with an IC_50_ value of ≤ 17.21 μg/mL. Notably, the FCFNCs demonstrated superior efficacy compared to functionalized CuNPs (FCNPs) and FeNPs (FFNPs), as evidenced by their ability to significantly increase p53 and caspase 3 expression while reducing the levels of Ki-67, a marker associated with cell proliferation. In terms of antimicrobial activity, FCFNCs displayed substantial growth reduction (> 70%) against a wide range of prokaryotic and eukaryotic pathogens, indicating their potential as effective agents against various microbial species [181].

### Algae-mediated synthesis of biogenic nanoparticles

An area of research in nanomedicine that is fast expanding is the algae-mediated production of biogenic NPs. Algae is photosynthetic organisms that can change inorganic materials into organic chemicals. AgNPs were synthesized using marine algae species extracts including *Cystoseira myrica*, *Gracilaria foliifera*, and *Ulva rigida*. These algal extracts acted as reducing and capping agents. The biosynthesized AgNPs exhibited dose-dependent cytotoxicity and demonstrated significant anticancer activity against MCF-7 breast cancer cells. The IC_50_ values for *G. foliifera*, *U. rigida* and *C. myrica* AgNPs against MCF-7 cells were 43 µg/ml, 13 µg/ml, and 13 µg/ml, respectively. *U. rigida* AgNPs showed the highest selectivity, inhibiting MCF-7 cell growth by 92.62%, while *C. myrica* AgNPs also exhibited significant anticancer activity [182]. Biogenic Ag-NPs have also been synthesized by *Chaetomorpha ligustica* [183] aqueous and the *Nannochloropsis* [184] algae extract, which were subjected to an in vitro evaluation of its antimicrobial, antioxidant and cytotoxic potential. NiO nanoparticles (NiO NPs) were synthesized using a cell-free extract derived from *Spirogyra* sp., a filamentous green macroalgae. The synthesized NiO NPs demonstrated antibacterial efficacy against both gram- positive and *gram-negative bacterial strains*, with an IC_50_ value of 10 mg/L. The antimicrobial activity showed concentration dependence, with a MIC of 10 mg/L. Additionally, the NiO NPs exhibited significant antioxidant potential as evaluated by DPPH and TAC assays [185].

Biogenically synthesized (*Champia parvula*) Cp-AuNPs exhibited a remarkable capability in terms of antioxidant activity and scavenging free radicals, which can be attributed to the presence of abundant antioxidant components. The anti-cancer efficacy of Cp-AuNPs was examined explicitly in A549 lung cancer cells, revealing a pronounced and promising anti-cancer potential. The study findings indicated that the synthesized Cp-AuNPs had significant therapeutic potential for lung cancer treatment [186]. In another study, *Cystoseira crinita*, a type of brown algae, was used to produce metabolites that served as a biocatalyst for eco-friendly magnesium oxide NP synthesis (MgO-NPs). The MgO-NPs exhibited noteworthy efficacy against diverse microorganisms, including Gram-positive bacteria, Gram-negative bacteria, and *Candida albicans*. The MIC values for the MgO-NPs ranged from 12.5 to 50 μg/mL. Furthermore, the MgO-NPs exhibited an IC_50_ value of 113.4 μg/mL against a cancer cell line (Caco-2) and 141.2 μg/mL against a normal cell line (Vero cell) [187].

### Fungi-mediated synthesis of biogenic nanoparticles

Another source of NP synthesis is fungi, and due to their affordability and eco-friendliness, their biosynthesis has recently attracted a lot of attention. *Aspergillus*, *Fusarium*, *Penicillium*, and *Trichoderma* are a few fungi extensively reported for NP synthesis [188], [189]. A study reported the synthesis of silver NPs via fungal endophyte *Penicillium oxalicum* i.e., (POAgNPs), which exhibited notable inhibitory effects against various pathogenic bacterial and fungal strains. The NPs exhibited potent antioxidant activity by effectively neutralizing various free radicals, including DPPH, superoxide, hydroxyl, and nitric oxide. Their EC_50_ values were 9.034, 56.378, 34.094 and 61.219 μg/mL, respectively. Additionally, the NPs demonstrated cytotoxic effects on MDA-MB-231 and MCF-7 BC cells, with IC_50_ values of 20.080 and 40.038 μg/mL, respectively. They also inhibited wound closure and induced nuclear morphology alterations in the cancer cells [190]. Kaplan and his teammates reported a microwave-assisted technique for synthesizing AgNPs using extracts from *Tricholoma ustale* and *Agaricus arvensis*. The antimicrobial properties of the synthesized AgNPs were evaluated against various gram-positive and gram-negative bacterial and fungal strains, using the MIC and disc diffusion methods. Cytotoxicity assessments on human BC (MCF-7), lung cancer (A549), colon cancer (HT-29), and osteosarcoma (Saos-2) cells revealed a dose-dependent anti-proliferative effect of the AgNPs, mediated by intrinsic apoptotic signaling pathways [191].

A change in brilliant yellow to dark brown coloration has been reported to be an effective indicator for NP synthesis. These AgNPs have remarkable antimicrobial properties against three types of Gram-positive and Gram-negative bacteria. Furthermore, they also displayed potent antioxidative capabilities, confirmed by a DPPH assay. Moreover, these AgNPs have also demonstrated the highest efficacy by inhibiting the growth of MCF-7 cells, reaching a rate of 62.69% at a concentration of 100 µg/mL and showing an IC_50_ value of 66.07 µg/mL [192]. Dias and his colleagues reported that Ag-NPs were synthesized via the extract from the reishi mushroom (*Ganoderma lucidum*). The synthesized Ag-NPs were assessed for antioxidant activity using the DPPH assay and investigated for their antimicrobial properties. MIC values were determined, revealing the antibacterial potential of Ag-NPs against gram-positive bacteria and gram-negative bacteria [193, 194].

*Cordyceps militaris*, a medicinal mushroom, has also been used to produce Cor-ZnO NPs. These NPs exhibit a moderate ability to counteract free radicals, as demonstrated by their effectiveness in scavenging DPPH, ABTS, hydroxyl radicals, nitric oxide, and superoxide radicals. Moreover, Cor-ZnO NPs show effective antibacterial activity against gram-positive and gram-negative bacterial strains [195]. However, there are a few drawbacks associated with this method, including the quality and purity of nanomaterials that get impacted by contamination caused by other microorganisms when microbes synthesize NPs. The yield of microbial biogenic nanoparticle synthesis is often very low, limiting its scalability for large-scale production. The cost of microbial biogenic NP synthesis is also high as specialized equipment and expertise are required, and some microbial species used in NP synthesis also generate toxic compounds that are harmful to human health and the ecosystem [196]. Owing to above stated challenges and obstructions, plants have emerged as an effective option for NP synthesis.

For centuries, plants have been well-known for their medicinal value by various tribes and cultures worldwide because plants encompasses diverse bioactive compounds like alkaloids, terpenoids, flavonoids and phenolic compounds that exhibit medicinal properties [197]. These benefits have promoted the use of plants for NP synthesis due to their characteristic advantages like non-toxicity, easy availability, environmental friendly, diversity of plant species and biocompatibility. Moreover, plant-mediated NP synthesis represents a highly efficient and economical strategy for producing an abundant amount of stable metal/metal oxide NPs. In contrast to conventional methodologies, plant-mediated synthesis primarily relies on aqueous extracts at ambient temperature and pressure, leading to substantial energy conservation. Due to these developments, the one-step plant-based NP synthesis has gained significant attention and has become a practical alternative to the above discussed conventional and other biogenic methods [198].

## Plant-mediated synthesis of biogenic nanoparticles

**Fig. 10:**
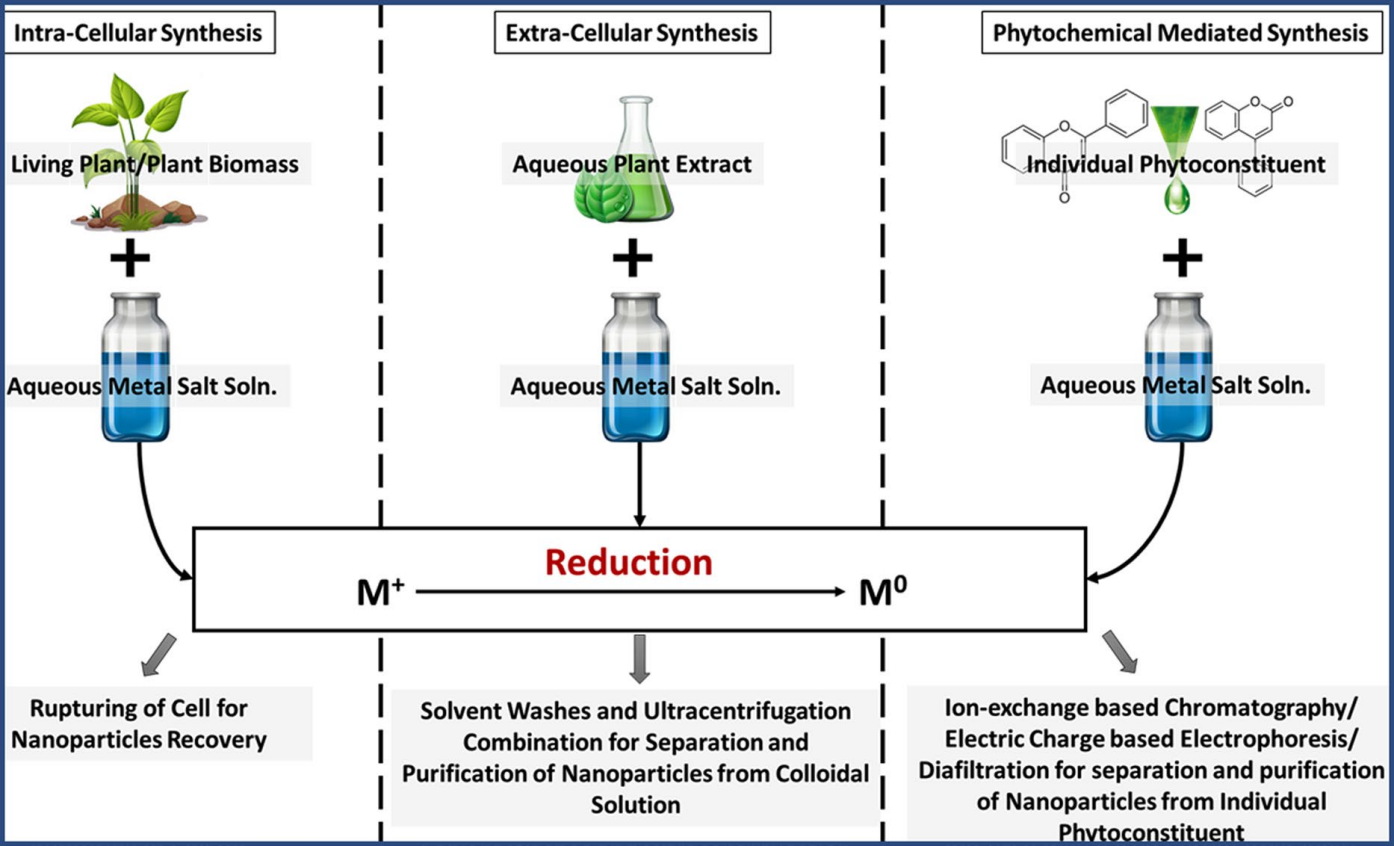
Illustration depicting plant-mediated approaches for nanoparticle fabrication

The biogenic synthesis of metal/metal oxide nanoparticles (NPs), including Au-NPs, Ag-NPs, and iron oxide NPs, using living plants (intra-cellular), plant extracts (extra-cellular), and phytochemicals, has gained considerable attention as an alternative to conventional methods. Various plant-mediated synthesis methods are commonly employed for this purpose, which is illustrated in Fig. 10.

Researchers are actively investigating biogenic approaches for synthesizing metal/metal oxide NPs. They are particularly interested in utilizing specific phytoconstituents, such as polyphenols, proteins, and organic acids, from plants with the aim of enhancing the precision and manipulation of the size and morphology during nanoparticle synthesis [199]. Broadly, the intracellular or plant biomass-based synthesis of biogenic NPs, extracellular or plant extract mediated synthesis of biogenic NPs and phytochemical based synthesis of biogenic NPs are the primary approaches for biogenic NPs that are discussed below as follows.

### Intracellular or plant biomass-mediated synthesis of biogenic nanoparticles

Researchers are currently focusing on utilizing various plant species phytoextraction and phytoremediation abilities to synthesize metal NPs, as some plants demonstrated significant potential for accumulating heavy metals. The hyperaccumulation process of trace metals from soil to shoots can be summarized as follows: (a) Metal activation in the rhizosphere via root-rhizosphere microbes interactions, (b) Increased uptake facilitated by metal transporters in cell membranes, (c) Metal detoxification through mechanisms such as binding to cell walls and chelation within the cytoplasm using ligands like metallothioneins, phytochelatins and metal-binding proteins, and (d) Sequestration of metals into the vacuole using tonoplast transporters [200].

The metallophytes *Brassica juncea* and *Medicago sativa* were inspected for their potential to accumulate and sequester Au from KAuCl_4_ solutions in their epidermis, cortex, and vascular tissue, with a higher concentration in xylem parenchyma cells. The size distribution of these NPs varied from 2 nm–2 μm in *B. juncea* and 2 nm–1 μm in *M. sativa*, and their distribution varied based on the plant location [200].

Marchiol and his teammates conducted in vivo synthesis of Ag-NPs in the roots, stems, and leaves of *Brassica juncea*, *Festuca rubra*, and *Medicago sativa* plants. Transmission electron microscopy (TEM) analysis affirmed the occurrence of Ag-NPs in different plant parts, including the cortical parenchymal cells and xylem vessel cell walls in the roots [201]. To date, only a few plant species, including *B. juncea*, *M. sativa*, [202], *Sesbania drummondii* [202], *Triticum aestivum* [203], *Avena sativa* [204], *Festuca rubra* [201] and *Chilopsis linearis*, have been reported to synthesize different metallic NPs via an intracellular route [205]. However, limited literature is available on the intracellular synthesis of metallic NPs due to the laborious and cost-effective steps involved in retrieving these NPs from plant biomass.

### Extracellular or plant extract mediated synthesis of biogenic nanoparticles

Plant extracts contain diverse phytochemicals, including phenolics, sugars, flavonoids, xanthones, and other substances. The synthesis of metal/metal oxide NPs by plants through a biogenic process can be simplified by directly extracting and using phytochemicals responsible for reducing metal ions. This approach is referred to as the extra-cellular method, which researchers prefer because it enables easier downstream processing and aids in scaling up the production as compared to the intra-cellular method for NP synthesis.

Scientific investigations have revealed that employing plant extracts for bio-inspired synthesis enables the development of NPs possessing a spherical shape and a highly reactive face-centered cubic (fcc) structure [206], which is energetically favorable. Studies have reported that NPs that prefer spherical growth along the (111) plane of fcc lattice demonstrate enhanced activity, making them more advantageous for various biomedical applications. The research findings indicate that plant extracts play a crucial role in controlling the growth of metal NPs, determining their shapes, sizes, and structures. These findings also shed light on the phytochemical basis and mechanism behind the biocompatible synthesis of Ag-NPs using the *Hypericum perforatum* extract. The study employed ultra-performance liquid chromatography coupled with a photodiode array and high-resolution mass spectroscopy for comprehensive analysis. The results obtained from the study revealed that the reduction of Ag^+^ ions is primarily mediated by phenolic acids and flavonoids present in the extract.

**Fig. 11:**
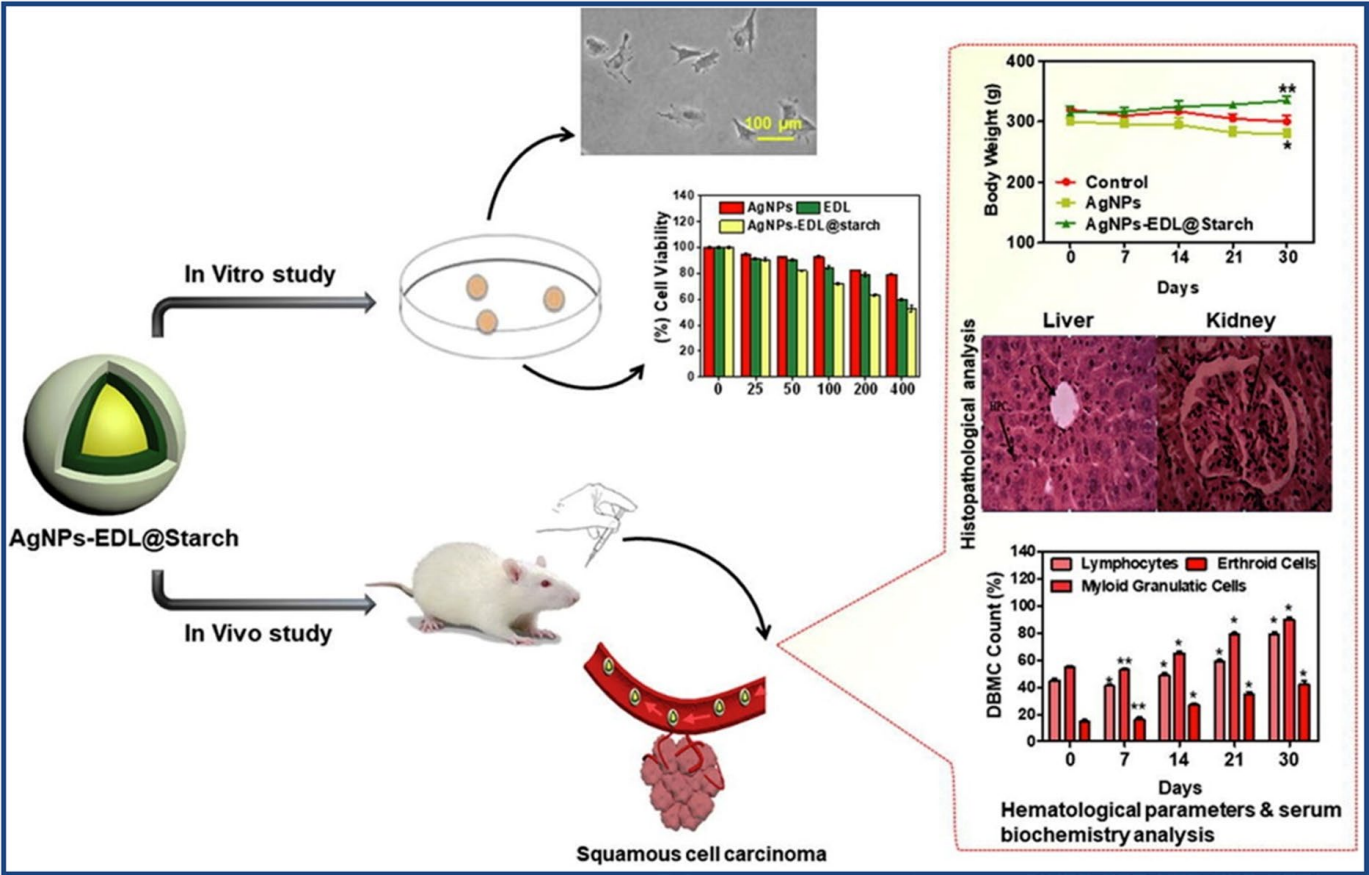
An overview of in vivo and in vitro assessment of grass-mediated biogenic synthesis of AgNPs. Symbol ‘g’ represent ‘Gram’, ‘%’ represents ‘Percentage’, ‘*’ signifies ‘p ≤ 0.05’ and ‘**’ signifies ‘P ≤ 0.001’. Reprinted with permission from Ref. [208]. Copyright 2021. Elsevier

The capping agents in the synthesis process of Ag-NPs include phloroglucinols and xanthones. Interestingly, the non-polar fraction of *H. perforatum* extract, rich in xanthones and phloroglucinols, did not possess the ability to reduce Ag^+^ ions. In contrast, the polar fraction of the extract, containing flavonoids and naphthodianthrones, along with specific compounds from these classes (like hypericin, kaempferol-3-glucoside, protohypericin, pseudohypericin, quercetin and quercetin-3-glucoside), demonstrated effective reduction of Ag^+^ ions [207]. The study conducted by Gul et al. (2021) introduced a novel approach using annual meadow grass (*Poa annua*) to produce AgNPs in an environmentally friendly manner. The antioxidant assay demonstrated an effectiveness of 61.42 ± 0.04% and 81.00 ± 0.12% for AgNPs and AgNPs-EDL@Starch, respectively. The hemolysis assay showed 8.9% and 3.8% rates for AgNPs and AgNPs-EDL@Starch, respectively. The MTT assay revealed that AgNPs inhibited SCC7 cell lines by approximately 60%, while AgNPs-EDL@Starch exhibited around 40% inhibition. Further, an in vivo assessment on Sprague Dawley rats (n = 3) demonstrated that a high dose of AgNPs (300 mg/kg) administered orally for 7 days did not induce significant cytotoxicity [208, 209]. The overview of this study is illustrated in Fig. 11.

Rani et al. (2023) utilized an *Azadirachta indica* leaf extract to synthesize ZnO NPs with concentrations ranging from 100–250 μg/mL. The anticancer activity of these ZnO NPs was assessed on SiHa cancer cell lines using the MTT assay. The results demonstrated a significant and dose-dependent cytotoxic effect, with decreased cell viability as the concentration of ZnO NPs increased. The IC_50_ values for L1, L2, L5, and L10 nanoparticles were 141 μg/mL, 132 μg/mL, 127 μg/mL, and 115 μg/mL, respectively, after 24 and 48 h of incubation [210]. Safdar et al. (2023) demonstrated a novel method for synthesizing selenium nanoparticles (Se NPs) using a *Bombax ceiba* flower extract. The Se NPs exhibited notable antibacterial activity, particularly against *Staphylococcus aureus*, with a zone of inhibition (ZOI) measuring 20 mm. Additionally, the Se NPs displayed significant inhibitory effects against *Klebsiella pneumoniae* and *Pseudomonas aeruginosa*, with a ZOI of 28 mm at a concentration of 100 µg/mL [211]. Another study reported the method of biogenic synthesis for developing cadmium sulfide quantum dots (CdS QDs) using a *Camellia sinensis* leaf extract. The study utilized a tea leaf extract as a stabilizing agent to form non-toxic quantum dots with particle sizes ranging from 2–5 nm.

The CdS quantum dots (QDs) exhibited strong fluorescence signals when interacting with A549 cancer cells, enabling high-contrast fluorescence imaging. Fluorescence emission analysis and flow cytometry techniques were utilized to investigate the bioimaging properties and cytotoxic effects of CdS QDs on A549 cells. Flow cytometry analysis revealed that CdS QDs arrested the growth of A549 cells at the S phase of the cell cycle, effectively inhibiting their proliferation [212].

Nagaraj et al. (2020) synthesized a nanocomposite of graphene oxide (GO) and ZnO using a *Dalbergia latifolia* leaf extract. The antibacterial potential of the nanomaterials, including ZnO-NPs, GO, and ZnO/GO nanocomposite (NC), was evaluated against *Staphylococcus aureus*. In vitro cytotoxicity studies on lung cancer cells (A549) and human BC cells (MCF-7) were conducted to assess the anticancer potential. The ZnO/GO NC exhibited significant anticancer activity against MCF-7 cells, with an IC_50_ value of 15 μg/mL, surpassing the efficacy of ZnO NPs and GO [213]. Padilla-Cruz et al. (2021) used a green synthesis method for creating Ag-Fe bimetallic nanoparticles with an aqueous extract of *Gardenia jasminoides* leaves. The synthesized NPs demonstrated magnetic properties and exhibited a synergistic antimicrobial effect, effectively killing bacteria. This effect surpassed the individual effects observed with the mono-metallic NPs when tested against yeast, as well as Gram-positive and Gram-negative bacteria strains resistant to multiple drugs [214]. Another study reported the biosynthesis of Ag-NPs using an aqueous leaf extract of *Cucumis prophetarum*, which was assessed for anti-proliferation potential against cancer cell lines [215].

Moreover, in two-dimensional and three-dimensional tumor models, ZnO-NPs synthesized by an extract of a *Annona muricata* leaf proved to be an efficient anticancer treatment [216]. The synthesis of MgO-NPs employing an aqueous extract of *Trachyspermum ammi* leaf via an eco-friendly approach has also been reported and evaluated for biological and medical applications [217]. A study reported a biogenic method to develop MgO-NPs using *Saussurea costus* biomass and has effective cytotoxic activity against MCF-7 cells [218]. *Abutilon indicum* leaf extracts facilitate the biogenic synthesis of CuO-NPs, which have been evaluated to impede the growth of BC (MDA-M-231) cancer cells [219]. Another study reported the biogenic synthesis of bimetallic ZnO-CuO NPs using a *Sambucus nigra* leaf extract, which showed significant cytotoxicity against A375 and A549 cells [220]. Ag-Au bimetallic NPs using a leaf extract of *Stigmaphyllon ovatum* were studied for their anticancer potential in laboratory conditions [221].

Additionally, the bio-inspired synthesis of CuO-NPs using pectin was assessed for its ability to combat human cervical cancer [222]. The biogenic synthesis of hematite (α Fe_2_O_3_) NPs using a *Rhus punjabensis* extract was assessed for their ability to fight against tumor and pathogenic diseases [223]. The anti-cancer potential of plant-mediated synthesis of iron oxide NPs using a *Punica Granatum* fruit peel extract has also been reported [224]. However, the extracellular or plant extract mediated synthesis of biogenic NPs is an effective approach for nanoparticle synthesis in contrast to intracellular plant-biomass-mediated nanoparticle synthesis, but plant extracts are known to contain many secondary metabolites that get adsorbed onto the surface of NPs resulting in the contamination of the NPs, further limiting their stability and performance.

#### Possible mechanism of plant extract-mediated biogenic nanoparticle synthesis

Fabricating a biogenic NP involves three essential components: stabilizing agents, reducing agents and a solvent medium. In the case of using plant extracts to synthesize metal/metal oxide NPs, the phytochemicals of the plant extracts serve as both reducing and capping agents. This approach typically involves an aqueous medium and is considered eco-friendly. Currently, extensive research is being conducted on using plant extracts for the biogenic synthesis of NPs [225].

However, due to the intricate nature and wide variety of phytochemicals found in the plant extracts, it is difficult to determine a particular bio-reducing and stabilizing agent responsible for developing and stabilizing the NPs. Phytochemicals like polyphenols (flavonoids, phenolic acids and terpenoids), organic acids, and proteins are considered potential bio-reducing and stabilizing agents for producing NPs. Research has reported that polyols (terpenoids and flavones) and polysaccharides found in the *C. zeylanicum* extract work together in reducing metallic ions [226]. Multiple phytoconstituents in the plant extracts act in synergy to reduce Ag ions [227]. Phytoconstituents refer to bioactive compounds found in plants that exhibit various medicinal and therapeutic properties. Specific phytoconstituents are selected based on their distinct chemical characteristics, including functional groups, antioxidant potential, and metal ion chelating ability. For example, flavonoids can enhance the antioxidant and antimicrobial properties of NPs [228], whereas alkaloids can contribute to their cytotoxic and anticancer ability [229]. Owing to the challenges of plant extract-mediated nanoparticle synthesis, researchers have started exploring the phytochemicals that can participate as a bio-reducing and stabilizing agent during the synthesis of biogenic NPs.

### Phytochemical mediated synthesis of biogenic nanoparticles

Phytochemicals, or natural compounds found in plants, have diverse properties like antioxidative, anti-inflammatory, anti-cancer, and cardiovascular protective effects, making them valuable for promoting health. The mechanism of plant extract-mediated synthesis of metal/metal oxide NPs is not yet completely comprehended. Several studies have reported that polyphenols and flavonoids may play a significant role in synthesizing and stabilizing metallic NPs [230]. Research analysis has indicated that the combined presence of polyols, specifically terpenoids [231] and flavones [228], along with polysaccharides, exhibits a synergistic effect in reducing metallic ions. Due to their distinctive chemical composition and intrinsic properties, individual phytoconstituents are frequently utilized in synthesizing biogenic NPs.

#### Polyphenols

Polyphenols, a class of phytochemicals found in plants, possess antioxidant properties due to their multiple phenolic rings. As previously said, flavonoids are a form of polyphenols that are further classified into six subgroups: anthoxanthins, flavanones, flavans, flavanonols, anthocyanidins, and isoflavonoids [232]. A study reported that the aqueous alcohol extract of pomegranate leaves (*Punica granatum*) which was fractionated in an ethyl acetate fraction, was analyzed for assessing the abundance of polyphenols. The primary phenolic compound in the fraction was isolated and identified as an ellagic acid through conventional and spectroscopic methods. In addition, AgNPs were successfully synthesized using the polyphenol-rich fraction as a catalyst. The antimicrobial activities of both the AgNPs and the polyphenol-rich fraction were evaluated against different microorganisms [233].

#### Flavonoids

Flavonoids, a type of phytoconstituents, serve as bio-reducing and stabilizing agents during plant-mediated NP synthesis. There are numerous phenolic hydroxyl groups that can decrease metal ions and start NP synthesis. They can also adsorb on the surface of the NPs, giving stability and decreasing agglomeration [232]. 'Green' nanomaterials mediated by flavonoids are stated to be a potential nanomedicine system to treat different health ailments. Kaempferol-Au-NCs, were synthesized and exhibited a specific affinity for cancer cell nuclei, causing targeted damage. Interestingly, this composite nanocluster demonstrated reduced toxicity towards normal human cells while displaying increased toxicity towards A549 lung cancer cells [234]. The utilization of flavonoid electron and hydrogen donation properties for synthesizing metallic NPs is presently a widely explored area [235].

#### Phenolic acid

Phenolic acids exhibit significant potential for NP synthesis as both capping and reducing agents. Numerous studies have documented the successful fabrication of metal NPs, such as Au, Ag, and platinum (Pt) using phenolic acids. This efficacy is attained through the hydroxyl groups present in phenolic acids, which possess electron-transfer capabilities, enabling the reduction of metal ions and subsequent NP formation. Additionally, the carboxylic acid group found in phenolic acids also functions as an effective capping agent, providing stabilization to the NPs and preventing their undesired aggregation. A study used cinnamic acid (CA) as a stabilizing agent to synthesize room temperature gold nanoparticles (Au-NPs), which exhibited self-assembly into a nanowire or pearl necklace structure depending on the molar ratio of CA to HAuCl_4_ and the pH of the colloidal solution. In another study, gallic acid (GA) served as both a capping and reducing agent in an environmentally friendly synthesis method for GA-AgNPs and GA-AuNPs. Further, cytotoxicity assessment was performed on human fibroblast cells (WI-38) over 24 h and 3 days, revealing complete biocompatibility of these NPs towards the tested cell line [236].

#### Terpenoids

Terpenoids, classified as isoprenoids, are naturally occurring chemical compounds synthesized by plants with a low molecular weight. They serve a wide range of biological purposes, such as those of pigments, scents, and hormones, as well as roles in defense against infections and predators. Carotenoids give fruits and vegetables vivid colors; menthol, a component of peppermint oil, and taxol, a chemotherapy medication derived from the Pacific yew tree, are a few well-known terpenoids [237]. A novel system utilizing terpenoid (xylaranic acid) Ag-NPs has been developed, improving solubility, bioavailability, and pharmacological properties. The enhanced anticancer efficacy involves apoptosis stimulation, validated through qRT-PCR analysis of apoptosis regulatory gene expression (p53, bcl-2, caspase-3) [231].

#### Proteins

Proteins are organic polymers consisting of amino acids that can regulate the emergence and expansion of NPs. A few proteins can also function as stabilizers, reducing agents, or templates for NP synthesis. Because of their complex structural makeup, proteins can be challenging to utilize in bio-reduction for NP synthesis. Previous studies have demonstrated that some biomolecules, such as amino acid residues, including arginine, cysteine, lysine, and methionine, can interact with silver ions [238]. Proteins and peptides that serve as templates for biomimetic mineralization synthesis, a process that involves the formation of inorganic nanoparticles (NPs) with specific characteristics. The resulting inorganic NPs are classified into various classes, including metal nanoclusters (MNCs), quantum dots (QDs), gadolinium derivatives, and metal sulfide nanoparticles (MSNPs). This approach utilizes the natural structures and properties of proteins and peptides to guide and control the formation of inorganic NPs, allowing for the development of biomimetic materials with tailored functionalities. By leveraging these biomolecular templates, scientists can explore the synthesis and application of different types of inorganic NPs for various purposes, ranging from biomedical imaging to catalysis and beyond [239].

#### Organic acid

A class of organic chemicals known as organic acids has one or more carboxyl (-COOH) functional groups. They are frequently present in plants and are crucial to many biological functions, including metabolism and signalling. According to published reports, these organic acids function as bio-reducing agents that can be employed in the production of various metallic nanoparticles. The presence of Ag^+^ and Au^3+^ ions in a solution, when exposed to an extract obtained from the leaves of *P. pedicellatum*, reduces these ions and the subsequent synthesis of Ag-Au bimetallic nanoparticles in the solution. The chemical constituents of the leaf extract, namely coumaric acid, catechin, protocatechuic acid and gallic acid, were identified as potential agents responsible for reducing, stabilizing, and capping the synthesized NPs [240]. In a study, malic acid which is another organic acid was employed as a surface modifying agent in the development of biocompatible Fe_3_O_4_ magnetic nanocarriers (MMNCs) for efficient delivery of the DOX. The primary objective of this study was to enhance the water dispersibility of the MMNCs while maintaining their biocompatibility. The optimization of electrostatic conjugation between the drug and the MMNCs involved varying the DOX to MMNCs ratio, resulting in a maximum loading efficiency of 72% at a ratio of 1:10. The DOX-coupled MMNCs exhibited pH-dependent controlled release properties. Furthermore, these DOX-MMNCs displayed cellular uptake that depended on the administered dose and retained the substantial cytotoxicity of DOX against the BC cell line MCF-7 [241]. Bio-inspired NPs have a lot of promise for developing more effective and tailored BC treatments.

### Limitations

#### Standardization challenges

The properties of biogenic nanoparticles can vary depending on factors such as the source material, extraction method, and environmental conditions, making standardization and quality control challenging.

#### Limited stability

Biogenic nanoparticles may exhibit lower stability compared to their synthetic counterparts, particularly under certain environmental conditions or storage conditions.

#### Scale-up issues

Scaling up the production of biogenic nanoparticles for commercial applications may pose challenges due to the variability in synthesis methods and the need for consistent quality.

#### Purity concerns

Biogenic nanoparticles synthesized from natural sources may contain impurities or contaminants, requiring thorough purification steps for certain applications.

Biogenic nanoparticles boast natural compatibility with biological systems, fostering their suitability for medical uses like drug delivery and diagnostics. Their utilization promotes environmental sustainability, adhering to green chemistry principles and lessening the ecological footprint of nanoparticle synthesis. With diverse functionalities and potential for novel properties, biogenic nanoparticles offer versatile applications across various industries, potentially garnering public acceptance due to their natural origin and perceived safety over synthetic counterparts. To further investigate this, the researchers discovered specific phytoconstituents that are involved in NP formation and focused on isolating these antioxidant constituents for the development of NPs. However, a few studies reported the utilization (direct) of phytochemicals for metallic NP synthesis [242]. However, more research is needed to optimize these NPs and assess their safety and efficacy in clinical studies [243].

## Characterization of biogenic nanoparticles

Nanoparticles derived from biological sources, with dimensions typically falling within the range of 1 to 100 nm, possess distinct and adjustable properties that set them apart from bulk materials. Their behavior is intricately tied to their size, influenced by factors such as their heightened surface area-to-volume ratio, quantum effects, and increased reactivity. Comprehensive characterization of these minute structures is essential for understanding and harnessing their diverse applications in fields like medicine, electronics, and catalysis. Initial analysis of nanoparticles entails the examination of their morphology, dimensions, and chemical composition through techniques such as high-resolution transmission electron microscopy (HR-TEM), scanning electron microscopy (SEM), and energy-dispersive X-ray spectroscopy (EDX). This analytical approach aids in discerning the distribution of metallic elements and any compositional variances within the biogenically derived nanomaterials. High-Resolution Transmission Electron Microscopy (HR-TEM) facilitated a comprehensive examination of the structural attributes of nanoparticles. Through this technique, invaluable insights were gained into the nanoscale morphology, crystal arrangement, and imperfections of the material. Analysis of both Au NPs and Pt NPs revealed the presence of spherical nanoparticles spanning sizes between 6 to 15 nm, along with the distribution of particle sizes. SEM is well known technique in research and industry to obtained the high-resolution imaging of versatile materials along with composition. SEM become more effective and powerful tool when coupled with EDX/EWS due to its accurate data prophecy ability regarding the composition, morphology, shape and size [175].

X-ray diffraction (XRD) is a valuable method utilized for analyzing the atomic and molecular structure of crystalline substances. By subjecting a sample to X-rays and observing the resulting diffraction pattern, XRD offers detailed insights into how atoms are arranged within the material. lattice spacing with a value of d = 0.23 nm is displayed, indicating the (111), (200), (222) and 311) planes of MgO nanoparticles, in accordance with reference No. 39-7746 from the International Centre for Diffraction Data (JCPDS). The optical properties of the nanoparticle, such as their absorption and scattering behaviors, were explored using UV–visible spectroscopy. This study provided clarification on the plasmonic characteristics of the biogenic nanostructures [218].

X-ray photoelectron spectroscopy (XPS), also known as electron spectroscopy for chemical analysis (ESCA), is a surface-sensitive technique that provides information about the elemental composition, chemical state, and electronic state of biogenic Ag nanoparticles. The analysis revealed the presence of C, O, N, Ag, along with minor traces of chlorine. High-resolution spectrum specifically focusing on the Ag 3d region. The analysis employing Gaussian–Lorentzian curves unveiled two distinct chemical states of silver. The curve attributed to Ag^0^ nanoparticles exhibited an electron binding energy of 368.5 eV for the Ag 3d5/2 level, with an energy separation (∆) of 6.0 eV between the Ag 3d5/2 and Ag 3d3/2 levels [244].

BET (Brunauer–Emmett–Teller) analysis was carried out to assess the specific surface area and porosity of the bimetallic nanoflowers. The biogenic Fe_2_O_3_ nanoparticles he synthesized exhibit a Type IV adsorption–desorption isotherm. Calculations revealed that the nanoparticles possess a (BET) surface area of 48.8 m^2^/g, a pore volume of 0.096 cm^3^/g, and a diameter of 7.9 nm [245].

## Breast cancer treatment from biogenic plant extract-mediated nanoparticles

Plant extracts, as sustainable and renewable sources, exhibit the potential to be utilized to synthesize biogenic NPs, thereby offering an environmentally benign substitute for synthetic chemical procedures. Lately, plant extracts have emerged as promising sources of therapeutic compounds for the treatment of BC as their extracts contain various bioactive constituents such as polyphenols, alkaloids, flavonoids, and terpenoids, which have already been identified for potent anticancer properties. They can effectively impede BC cell proliferation, induce programmed cell death (apoptosis), inhibit angiogenesis (formation of new blood vessels to supply tumors), and reduce inflammation. Preclinical studies have shown the remarkable efficacy of several plant extracts against BC. For example, extracts from *Taxus brevifolia* (Pacific yew) [246] and *Catharanthus roseus* (Madagascar periwinkle) [247] contain compounds such as paclitaxel and vinblastine, respectively. These compounds are well-known to interfere with crucial cellular processes and exhibit potent anticancer activity.

Additionally, traditional medicinal plants like *Curcuma longa* (turmeric) [248] and *Trigonella foenum-graecum* (fenugreek) [249] have been investigated for their anti-breast cancer properties. Curcumin from turmeric and diosgenin from fenugreek has shown promising results in inhibiting cancer cell growth, promoting apoptosis, and suppressing tumor progression. Although plant extracts hold great promise for BC treatment, further research fully elucidating their mechanism of action, optimizing their formulation and dosage, and evaluating their safety and efficacy in clinical trials is needed. Nonetheless, the rich reservoir of bioactive compounds present in plant extracts offers immense potential for developing new biogenic NPs and effective therapies for BC patients. The biocompatibility of these biogenic NPs implies that they are less likely to have adverse effects on the body, which is a crucial advantage of this approach. Furthermore, biogenic NPs can be fabricated for targeting, particularly cancer cells, increasing their effectiveness and minimizing harm to healthy cells.

### Metal nanoparticles

Metal NPs are nanoscale particles composed of metals that offer promising opportunities in the medical field, with their unique properties being harnessed for applications such as targeted drug delivery, imaging, diagnostics, and therapeutic intervention [250]. Krishnaraj et al. (2014) reported Ag-NPs and Au-NPs biogenically synthesized using an *Acalypha indica Linn* extract. The NPs exhibited significant cytotoxic effects against MDA-MB-231 cell as demonstrated by MTT assays, AO/EB staining, caspase-3 assays, and DNA fragmentation assays, particularly at a concentration of 100 μg/ml, indicating apoptotic characteristics [251]. Au-NPs were synthesized using an aqueous extract of *Commiphora wightii*, and their in vitro anticancer activity against MCF-7 cells was evaluated, with an IC_50_ value of 66.11 μg/mL. Cw@AuNPs induced a significant increase in apoptotic cells at higher concentrations and caused the arrestment of MCF-7 cells at the G2/M phase of cell cycle [252].

Ganeshkumar et al. (2013) developed a novel technique using a *Punica granatum* fruit peel extract to synthesize monodispersed gold nanoparticles (PAuNPs). A toxicity assessment of PAuNPs and 5-fluorouracil (5-Fu), as well as 5-Fu loaded onto PAuNPs (5Fu@PAuNPs), was performed on zebrafish embryos. In vitro cytotoxicity studies on MCF-7 cells showed that the IC_50_ value of 5Fu@PAuNPs-Fa was significantly lower compared to free 5-Fu [253]. Chen et al. (2022) reported the facile method for synthesizing AuNPs utilizing a *Foeniculum Vulgare* aqueous extract as a green reducing and stabilizing agent. The cytotoxicity and anti-breast cancer effects of Au chloride, a *Foeniculum Vulgare* aqueous extract, and AuNPs were assessed on ZR-75–30, T47D, and HCC1187 breast cancer cell lines using the MTT assay. Results indicated that AuNPs had superior anti-breast cancer properties without cytotoxicity, and they displayed dose-dependent activity against the cancer cell lines while being non-toxic to a normal cell line (HUVEC) [254]. In another study, Zi-AuNPs were synthesized with the help of an aqueous extract of *Ziziphus spina-christi* leaves. The evaluation of cytotoxicity was performed using a MTT assay, which demonstrated a notable anticancer efficacy for both Zi-AuNPs and a *Z. spina-christi* extract against human BC cells (specifically, the MCF7 cell line) with IC_50_ values of 48 and 40.25 μg/mL, respectively [255]. Pechyen et al. (2022) reported Au-NPs synthesis using a *Spondias dulcis* peel extract (SPE) as a biogenic reducing and stabilizing agent. Furthermore, the toxic effects of SPE-Au-NPs on Vero normal cells and MCF-7 BC cells were investigated using the MTS assay. Cell viability was assessed at different concentrations of SPE-Au-NPs (50, 100, 200, and 400 μg/mL). The study showed that the viability of MCF-7 cells was dose- and time-dependently inhibited compared to the control cells. Conversely, Vero cells, serving as a model for normal human cells, exhibited lower cytotoxicity even at a high concentration of 400 μg/mL of SPE-Au-NPs [256]. Au-NPs were synthesized using Aloe vera, honey, and *Gymnema sylvestre* leaf extracts and were conjugated with three anticancer drugs. The study achieved targeted drug delivery to breast cancer cells while minimizing cytotoxicity against normal cells. The AuNPs derived from Aloe vera showed significant effectiveness in treating MCF7 and MDA-MB-231 cell lines when combined with (Z)-4-Hydroxytamoxifen [257].

**Fig. 12:**
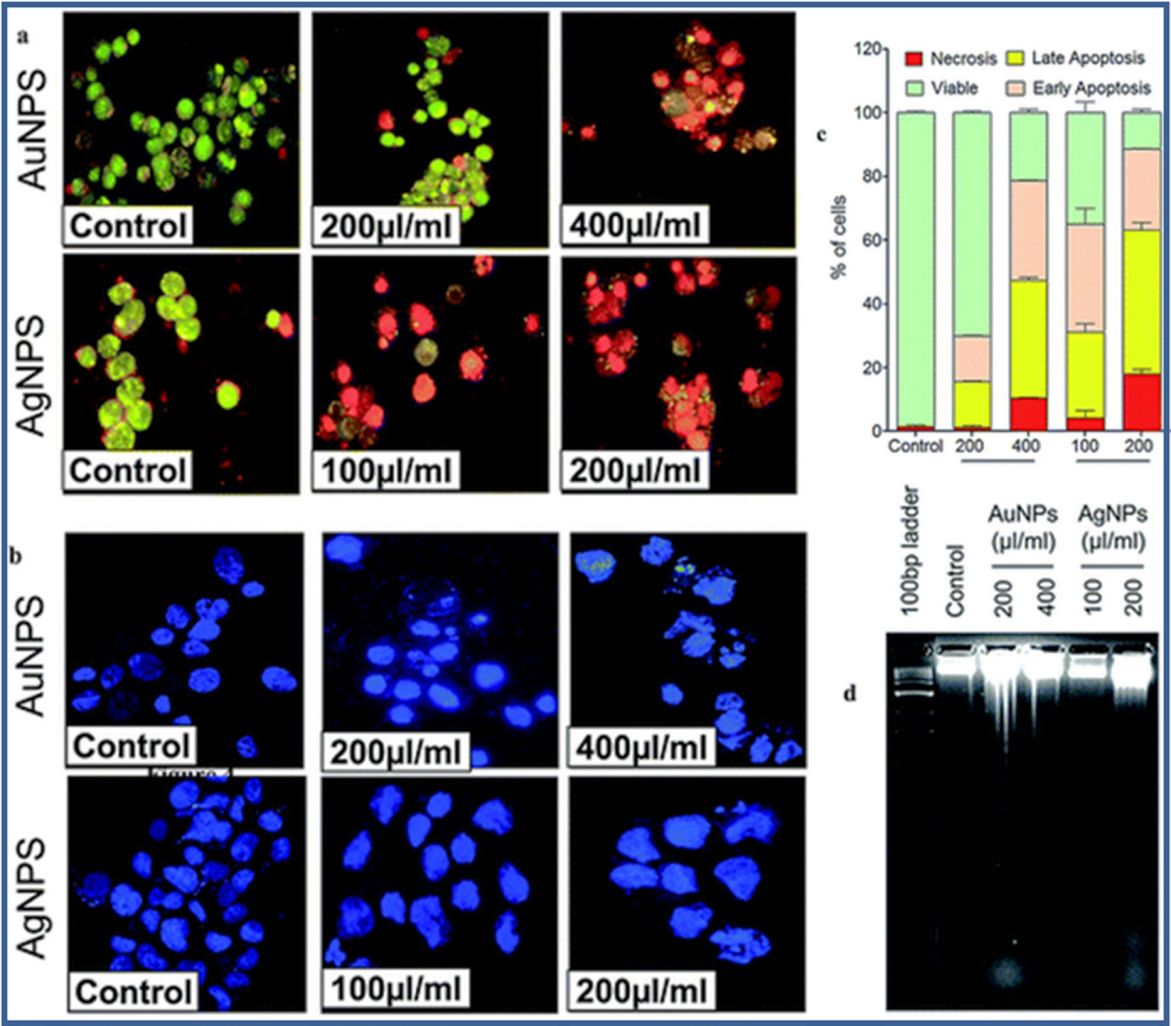
Apoptosis induced by Au-NPs and AgNPs in MCF-7 cells. **a** Photomicrographs of MCF-7 cells stained with AO/EB after 24 h incubation with Au-NPs or AgNPs. Viable cells appear light green, early apoptotic cells appear in bright green fluorescence, late apoptosis cells appear in red to orange fluorescence and necrosis cells appear in red fluorescence. **b** Representative fluorescent micrographs of MCF-7 cells stained with Hoechst 33,258 fluorescent dye after 24 h incubation with either Au-NPs or AgNPs at ×200 Magnification. **c** Percentage distribution of apoptotic cells including viable, early apoptotic, late apoptotic and necrotic cells. **d** AgNPs induced inter-nucleosomal DNA fragmentation. MCF-7 cells were treated with different concentrations of AgNPs (0, 100 and 200 μl ml^−1^) for 24 h. Cells were harvested, and DNA was extracted. Fragmented DNA was extracted and analyzed via agarose gel electrophoresis. Representative gels from one of the three experiments. Reprinted with permission from Ref. [259]. Copyright 2011 RSC advances

A study investigated the effects of Ag and Au-NPs on MCF7 cells, observing a time- and dose-dependent decrease in cell viability. Apoptosis induction by the NPs was assessed through caspase activation, condensation of chromatin, and the formation of apoptotic bodies containing intact cellular components. AOEB staining was used to detect these apoptotic changes after exposure to the NPs [258]. The results obtained from the study revealed the more significant apoptotic effect of NPs (as shown in Fig. 12a) compared to the control cells. Further, the impact of NPs on the nuclear morphology of MCF7 cells was assessed using Hoechst 33,258 staining and fluorescence microscopy (as shown in Fig. 12a). After 24 h of NP treatment, apoptotic characteristics like nuclear blebbing, condensation, and fragmentation were observed (as shown in Fig. 12b). In contrast, the control group exhibited regular cell morphology with minimal detachment (as shown in Fig. 12b). The distribution of apoptotic cells, including viable, early apoptotic, late apoptotic, and necrotic cells, is represented in Fig. 12c. [259]. The critical outcome of this research is that the administration of Ag-NPs and Au-NPs resulted in a reduction of the MCF7 cell population in the G1 phase of the cell cycle and an increase in the subG1 phase (as shown in Fig. 12d). Moreover, the mRNA expression levels of p53 were observed to be upregulated in the NP-treated cells, confirming the activation of p53-mediated DNA damage response [259].

Another study reported the cytotoxic properties of Tin NPs synthesized from a *Calendula officinalis* leaf extract (SnNPs@C. officinalis), which were evaluated using the MTT assay on both normal (HUVEC) and breast malignancy cell lines, including MCF7, Hs 319.T, and MCF10 cells. In this study, the treated cells were exposed to various concentrations of SnNPs@*C. officinalis* for 48 h. interestingly, even at concentrations as high as 1000 μg/mL, SnNPs@*C. officinalis* exhibited negligible cytotoxicity towards the normal cell line (HUVEC), while demonstrating a dose-dependent decrease in viability in malignant breast cell lines. The IC_50_ values of SnNPs@*C. officinalis* were determined to be 132 µg/mL, 126 µg/mL, and 119 µg/mL against MCF7, Hs 319.T, and MCF10 cell lines, respectively [260]. Venugopal et al. (2017) assessed the cytotoxic effects of Ag-NPs synthesized with a *Syzygium aromaticum* extract on MCF-7 breast cancer and A549 lung cancer cell lines. The IC_50_ values of the Ag-NPs were determined to be 60 μg/mL for MCF-7 cells and 50 μg/mL for A549 cells, while the IC_50_ for *Syzygium aromaticum* extract was 70 μg/mL for both cell lines [261]. Hemlata et al. (2020) utilized an aqueous extract of *Cucumis prophetarum* leaves to synthesize AgNPs, which were then tested for their antiproliferative activity on cancer cell lines. The IC_50_ values of Cp-AgNPs on A549, MDA-MB-231, HepG2, and MCF-7 cell lines were 105.8, 81.1, 94.2, and 65.6 μg/mL, respectively, indicating higher potency against MCF-7 breast cancer cells [215]. Ag/AgCl NPs were synthesized using a *Z. mauritiana* fruit extract and demonstrated significant antiproliferative effects on MCF-7 and EAC cells, with IC_50_ values of 28 and 84 μg/mL, respectively, through the Fas pathway. In MCF-7 cells, these NPs completely prevented colony formation compared to the control group. Additionally, Ag/AgCl NPs triggered apoptosis and promoted the generation of reactive oxygen species (ROS) in MCF-7 cells. Upon assessment, a significant increase in the levels of caspase-8, FADD and FAS genes expression was accorded, while a substantial decrease in the expression of the PARP gene was accorded [262]. A scientific investigation revealed the ability of the fruit shell derived from *Tamarindus indica*, a pharmacologically significant tree, to biosynthesize Ag NPs. These synthesized Ag NPs demonstrated cytotoxic effects against MCF-7 cell lines associated with BC. A cytotoxicity study was conducted by using an MTT assay exhibiting a dose-dependent response, and the inhibitory concentration (IC_50_) was determined to be 20 μg/ml. The anticancer activity of Ag NPs was authenticated by performing live and dead assays (using Ao/EtBr), as well as ROS and Rho123 assays [263]. Another study demonstrated the eco-friendly synthesis of Ag NPs using methanolic extracts of *F. cretica*, which were then conjugated with albumin and encapsulated in liposomes. The efficacy of these Ag NPs was evaluated using an MTT cell viability assay on MCF-7, Hep-2, HUH-7, and HCEC cell lines. The expression of genes related to the apoptotic pathway was assessed using quantitative real-time polymerase chain reaction (qRT-PCR). Upon assessment, the zeta potentials of liposomes, Ag NPs, and albumin NPs were determined to be −18.6 mV, −15.5 mV, and −18.3 mV, respectively. In vitro, Ag NPs showed strong anticancer potential, surpassing albumin and liposome NPs. The IC_50_ values of Ag NPs on MCF-7, Hep-2, and HUH-7 cell lines were determined to be 0.101 ± 0.004 mg/mL, 0.177 ± 0.03 mg/mL, and 0.434 ± 0.022 mg/mL, respectively [264]. Ansar et al. (2020) reported the environmentally friendly synthesis of Ag-NPs using *Brassica oleracea* (BO) and examined their antibacterial, anticancer, and antioxidant properties. The cytotoxicity of green-synthesized BO-Ag NPs exhibited a proportional increase with higher concentrations. The maximum cytotoxic effect was observed at a concentration of 100 μg/ml, with an IC_50_ value of 55 μg/ml [265]. A study was conducted which reported the conjugation of Ag-NPs with a *Rubus fairholmianus* extract and the bio-conjugated product formed was tested for its effects on apoptotic proteins such caspase 3, Bax, and P53 using ELISA and western blotting. Upon assessment, the exposure of RAgNPs showed reduced cell growth and increased cytotoxicity in MCF-7 cells. Compared to untreated cells, 10 μg/mL RAgNPs increased cytotoxicity 1.83-fold (P < 0.05). All RAgNP concentrations caused nuclear damage and intracellular ROS generation. Moreover, Caspase 3, Bax, and P53 protein expression was determined to be the highest at 5 and 10 μg/mL (P < 0.05, 0.01) [266].

Lichens, specifically *Xanthoria parietina* (Xa) and *Flavopunctelia flaventior* (ff) [267], along with an extract obtained from *Viburnum nervosum* [268], were utilized to biosynthesize Ag-NPs for their potential application against MCF-7 cells. Another study reported the cytotoxicity of an aqueous extract derived from *Beta vulgaris,* which was also evaluated against MCF7, A549, and Hep2 cell lines, demonstrating an IC_50_ value of approximately 70 μg/ml after 48 h of incubation. Furthermore, the cytotoxicity of Ag NPs synthesized from *Beta vulgaris* was assessed against the same cell lines, revealing IC_50_ values of approximately 47.6 μg/ml, 48.2 μg/ml, and 47.1 μg/ml for MCF7, A549, and Hep2, respectively, after 48 h of incubation [269]. Notably, Xa-AgNPs and Ff-AgNPs exhibited a more pronounced cytotoxic effect than their respective lichen extracts, Xa and Ff. The study indicated that Xa-AgNPs and Ff-AgNPs had higher cytotoxicity against FaDu and HCT 116 cell lines than MDA-MB-231 cells. Moreover, Ff-AgNPs exhibited greater efficacy compared to Xa-AgNPs [269]. Importantly, in vitro studies on VN extract using BC cells (MCF-7) and epidermal carcinoma cells (A431) demonstrated biocompatibility. This study used a *Ceropegia bulbosa Roxb* extract to synthesize biogenic selenium nanoparticles (Se NPs). The in vitro cytotoxic potential of Se-NPs was evaluated using MTT tests at different concentrations (0, 5, 10, 15, 20, 25, 30, 35, 40, 45, 50 μg/mL) on HBL-100 and MDA-MB-231 cell lines. Se NPs showed anti-proliferative and concentration-dependent cytotoxic action. After 48 h, Se NPs killed 50% of MDA-MB-231 cells, revealing an IC_50_ value of 34 μg/mL. After 48 h of exposure, cytotoxicity increased when the inhibitory concentration approached 50 μg/mL. Se NPs interacted with the target protein BRCA2, which contained amino acids with high electronegativity on its surface. Through the implementation of the Glide docking protocol, the binding energy between Se-NPs and BRCA2 protein was determined to be approximately −6.45 kJ/mol [270]. Pt NPs were biogenically synthesized via a *Nigella sativa* L. extract, serving as a reducing agent. The cytotoxic effects of these Pt NPs were evaluated on HeLa and MDA-MB-231 cancer cell lines using the MTT assay and microscopic visualization. The Pt NPs coupled with *Nigella sativa* L. extract exhibited higher cytotoxicity on MDA-MB-231 and HeLa cell lines, with increasing concentrations (25–150 µg) leading to a noteworthy inhibition of cellular proliferation after 24 h. The highest anti-proliferative effect was observed with 150 µg of Pt NPs stabilized with a *Nigella sativa* L. extract, resulting in 93% and 96% inhibition of cellular proliferation in MDA-MB-231 and HeLa cell lines, respectively [271].

### Metal oxide nanoparticles

**Fig. 13:**
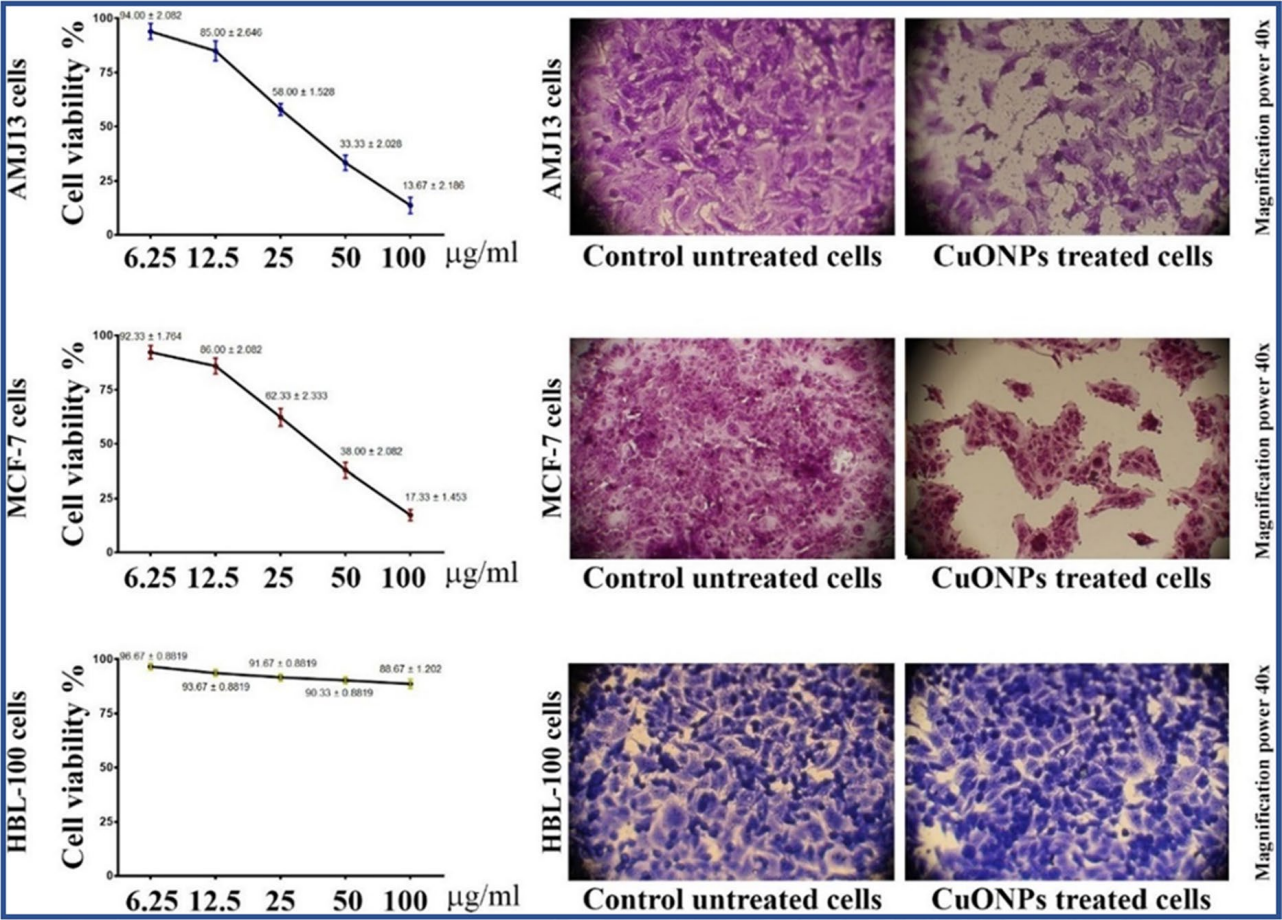
The antiproliferative activity of CuO NPs against AMJ-13, MCF-7, and HBL-100 cancer cell lines. Triplicate samples of each cancer cell lines were prepared (concentration of 1 × 10^4^ cells/mL) and treated with CuO NPs, which were subjected to 72 h incubation. Viability of cells was assessed via MTT assay by measuring the absorbance at a 492 nm wavelength. (Under Creative Commons CC BY License)

Metal oxide NPs are nanoscale particles composed of metal and oxygen atoms, and their remarkable properties make them highly attractive for diverse applications ranging from targeted drug delivery and imaging to regenerative medicine and therapeutics [272]. *Cantaloupe* peels were used to produce and functionalize bacterial cellulose (BC) with biogenic copper oxide nanoparticles (CuO NPs) synthesized from a *Punica granatum* peel extract. The cytotoxicity of the resulting BC/PVA/Ch/CuO NPs composite was assessed using an MTT assay on normal and cancer cell lines. The viability of normal HSF cells was higher than that of cancer cells when treated with the composite. Increasing the concentration of CuO NPs in the composite resulted in lower IC_50_ values, indicating increased cytotoxicity. The control membrane (BC/PVA/Ch) showed no cytotoxic effects. The composite had low IC_50_ values against Caco-2, HepG-2 and MDA cell lines. Among the tested composite membranes, Caco-2 cells were highly susceptible to treatment [273]. In a study conducted by Mahmood et al. (2020), a bio-synthesis approach was employed to synthesize CuO-NPs using an *Annona muricata L* plant extract. The cytotoxic effects of the synthesized CuO NPs were evaluated on HBL-100, AMJ-13, and MCF-7 cell lines. The anti-tumor characteristics of the CuO NPs were assessed by examining their ability to inhibit the proliferation of malignant cells. The results obtained from the study demonstrated significant and highly toxic effects of the synthesized CuO NPs on AMJ-13 and MCF-7 cells. However, there was no evidence of cytotoxicity induced by the CuO NPs on the healthy HBL-100 cells. Nonetheless, the nanoparticles exhibited anticancer properties by inhibiting or blocking the growth of cancer cells, as illustrated in Fig. 13. The AMJ-13 and MCF-7 cell lines showed a similar response to the exposure of CuO NPs, indicating their potential to reduce the proliferation of cancer cells. Furthermore, when assessing the genotoxic potential of two *A. muricata* extracts on MCF-7 cells using the alkaline comet assay, it was observed that these extracts exhibited higher genotoxicity. This finding suggests that the *A. muricata* extracts can induce DNA damage in MCF-7 cells [274].

Another study reported the production of CuO NPs using an *Olea europaea* leaf extract. After 24 h of exposure to CuO NPs (0.39 to 50 mg mL^−1^), AMJ-13 and SKOV-3 BC cell lines were tested via MTT assays. Upon assessment, the CuO NPs inhibited AMJ-13 cell growth in a concentration-dependent manner. A dose of 50 mg mL^−1^ CuO NPs inhibited 94% cell death, while 0.39 mg mL^−1^ caused 10% cell death. Moreover, the *O. europaea* extract had less cytotoxicity on AMJ-13 cells than CuO NPs. SKOV-3 cells also showed similar results with an IC_50_ value of 2.27 mg mL^−1^. Furthermore, the normal cells had less cytotoxicity than cancer cells, although, at 200 mg mL^−1^, they had 78% toxicity [275]. CuO NPs bio-functionalized from *Eucalyptus globulus* exhibited an apoptotic effect on human BC cells and toxicity against *Aspergillus flavus*. Microwave (MW) treatment enhanced the activity of electrochemically-engineered CuO NPs (16.9 nm) on MCF-7 cells at concentrations of 25–100 μg/ml, resulting in a significant decrease in cell viability. This decrease in viability was found to be associated with disintegration of mitochondrial membrane potential, aggregation of ROS within the cells, and disturbance of the cell cycle progression. Additionally, the mRNA expression levels of critical genes involved in apoptosis, including p53, bax, caspase-3, and caspase-9, increased in MCF-7 cells treated with the synthesized electrochemically-engineered CuO NPs, further confirming their mechanistic anti-cancer potential [276].

CuO NPs were also synthesized using *Helianthus tuberosus* (Ht) plant extracts and further encapsulated with starch (ST) and coupled with folic acid (FA) for targeted release in MDA-MB-231 cells. The cytotoxicity evaluation revealed that FA-ST-Ht-CuO NPs exhibited a higher cytotoxic effect on MDA-MB-231 cells, with an IC_50_ value of 21.03 μg/mL. This cytotoxicity was attributed to the activation of ROS production, nuclear damage, reduction in mitochondrial membrane potential, and upregulated expression of apoptosis-related proteins [277]. Studies have been carried out to explore the antimicrobial and antiproliferative properties of copper nanoparticles (Cu-NPs) synthesized using leaf extracts of *Manilkara zapota* [278] and *Wrightia tinctoria* [279]. The Cu-NPs showed minimal hemolytic activity in in vitro haemolytic assays, with hemolysis percentages of 5.73%, 3.34%, and 0.5% at concentrations of 100, 50, and 25 μg/mL, respectively. In the antiproliferative assay, the IC_50_ values were determined as 53.89 μg/μL for MCF7 (BC cells) and 883.69 μg/μL for Vero cells (kidney epithelial cells). Intriguingly, in vitro cytotoxicity studies revealed that Wt-CuNPs-treated MCF-7 cells and Vero cells exhibited 50% cell death at concentrations of 119.23 μg/mL and 898.75 μg/mL, respectively.

In another study, iron oxide NPs were efficiently synthesized using the *Celosia argentea* leaf extract. The cytotoxicity of these NPs was evaluated on MCF-7 breast cancer cells using MTT analysis and dual staining with Acridine Orange/Ethidium Bromide, along with a Hoechst control. Two concentrations of the synthesized NPs, 25 and 50 μg/mL were employed, while cancer cells without NPs served as the control. The IC_50_ value was determined to be 25 μg/mL. These findings revealed that iron oxide NPs accounted for 50% inhibition of cancer cells. Notably, a significantly higher inhibition percentage of 86 ± 3.59% was achieved with a concentration of 50 μg/mL after a 24-h exposure period [280]. In a study conducted by Rahmani et al. (2020), superparamagnetic iron oxide nanoparticles (SPIONs) were synthesized using iron salts in aqueous extracts derived from *Aloe Vera* leaves and *Flaxseed*. The potential cellular toxicity of these NPs was evaluated on a MCF-7 cell line, considering a wide range of concentrations up to 4.7 μg/ml. The observed cytotoxicity in the samples was influenced by multiple factors such as disintegration products, synthetic compounds, cell type, substrate coverage, and related variables. The study also recognized the cytotoxic effects of the composite sample (*Aloe vera*/SPIONs) by accordance with scientific standards [281].

Fe_3_O_4_ NPs were synthesized using an extract from *Ocimum basilicum* leaves. The cytotoxicity of Fe_3_O_4_ NPs on MDA-MB-231 was evaluated at different concentrations. After 48 h, IC_50_ of Fe_3_O_4_ NPs mediated by the leaf extract was determined as 17.75 µg/mL. These findings highlight the significant cytotoxic properties of Fe_3_O_4_ NPs against MDA-MB-231 breast cancer cells, suggesting their potential as a therapeutic agent [282]. Sulaiman et al. (2017) reported the synthesis of magnetic iron oxide nanoparticles (MNPs) using an *Albizia adianthifolia* leaf extract. The viability of AMJ-13 and MCF-7, BC cells, was assessed using the MTT assay. After 24 h exposure to various concentrations of MNPs, ranging from 1.9–62.5 lg/mL. The AMJ-13 cell line showed a significant inhibitory effect on cell growth following treatment with MNPs compared to control cultures (P < 0.05). Furthermore, the degree of inhibition exhibited a concentration-dependent pattern. The highest level of inhibition (92%) was observed at an MNPs concentration of 62.5 lg/mL, while a concentration of 1.9 lg/mL resulted in 53% cell death [283].

ZnO NPs were biogenically synthesized using *Pandanus odorifer* leaf extract. MCF-7, HepG2, and A-549 cells were exposed to various concentrations (1–100 μg/mL) of ZnO NPs for 24 h. Cell viability remained within the range of 80–100% at concentrations of 1–25 μg/mL. However, at higher concentrations of 50 and 100 μg/mL, the viability of all cancer cells decreased to 70% and 60%, respectively. Further analysis focusing on MCF-7 cells revealed that approximately 40% underwent total cell death, with 15.5%, 16.23%, and 8.27% corresponded to apoptotic, necrotic, and late apoptotic cells, respectively [284]. ZnO NPs were synthesized using an extract from *Hertia intermedia* as a reducing and stabilizing agent. The cytotoxic effects of ZnO NPs were evaluated on Caco-2, SH-SY5Y, MDA-MB-231, and HEK-293 cell lines using the MTT assay. The IC_50_ values for these cell lines were found to be 177, 184, 168 and 240 μg/mL, respectively. The production of ROS was measured using the DCFH-DA assay, indicating increased ROS levels after 24 h of treatment with 200 μg/mL ZnO NPs, suggesting the induction of oxidative stress [285]. Another study focused on utilizing the pumpkin (*Cucurbita pepo* L.) seed leaf extract for the biogenesis of ZnO NPs and assesses their cytotoxicity on the MDA-MB-231 cell line. The synthesized ZnO NPs exhibited dose-dependent cytotoxic effects, with a 50% inhibitory concentration (IC_50_) value of 10 µg/mL [286].

Khaleghi et al. (2022) reported the synthesis of ZnO NPs using leaf extract from *Origanum majorana*. According to the cytotoxicity evaluation in MCF7, HT-29, and normal cells (HFF), the IC_50_ concentrations after 48 h treatment were determined to be 16.8 6.7, 194.3 5.84, and 33.5 g/ml, respectively [287]. A novel methodology was developed by Al-Ajmi et al. (2018) for the synthesis of highly crystalline ZnO NPs using an *Alstonia macrophylla* leaf extract. The cytotoxic effects of ZnO NPs were investigated on A-549, HepG2 and MCF-7 cell lines at concentrations ranging from 1–100 μg/mL. The results from diverse cytotoxicity assays and morphological analyses demonstrated a significant concentration-dependent reduction in cancer cell viability upon exposure to ZnO NPs [288].

There have been reports on the environmentally friendly biosynthesis of polycrystalline titanium dioxide (TiO_2_ NPs) using the leaf extract of *Justicia gendarussa* [289] and *Limonia acidissima* [290]. The impact of these TiO_2_ NPs on the proliferation of MCF-7 and MDA-MB-231 cells was examined by subjecting the cells to varying concentrations of the NPs. In the case of the MCF-7 cell line, the viability dropped from 89.02% at a concentration of 1.0 μM to 55.64%. Similarly, in the MDA-MB-231 cell line, the viability decreased from 92.92 to 53.82% at the same concentration of 1.0 μM TiO_2_. The concentration-dependent manner in which the viability of both cell lines decreased suggests that TiO_2_ NPs can reduce cell viability.

*Synadenium cupulare* plant extracts were used as precursors for synthesizing a CdO/CdCO_3_ nanocomposite, using cadmium nitrate tetrahydrate as the precursor salt. The cytotoxicity of the standard drug emetine was assessed, showing a significant inhibitory activity with an IC_50_ value of 0.002 μg/mL. The inhibitory effects of annealed as well as unannealed CdO/CdCO_3_ nanocomposites on MCF-7 cell lines were investigated. The annealed nanocomposite demonstrated substantial inhibition, with an IC_50_ value of 0.652 μg/mL, while the unannealed nanocomposite exhibited lower inhibition, with an IC_50_ value ≥ 100 μg/mL. The CdO/CdCO_3_ nanocomposite inhibited cell growth, with the annealed and unannealed forms exhibiting IC_50_ values of 3.770 and 3.088 μg/mL respectively [291].

### Metal sulfide quantum dots

**Fig. 14:**
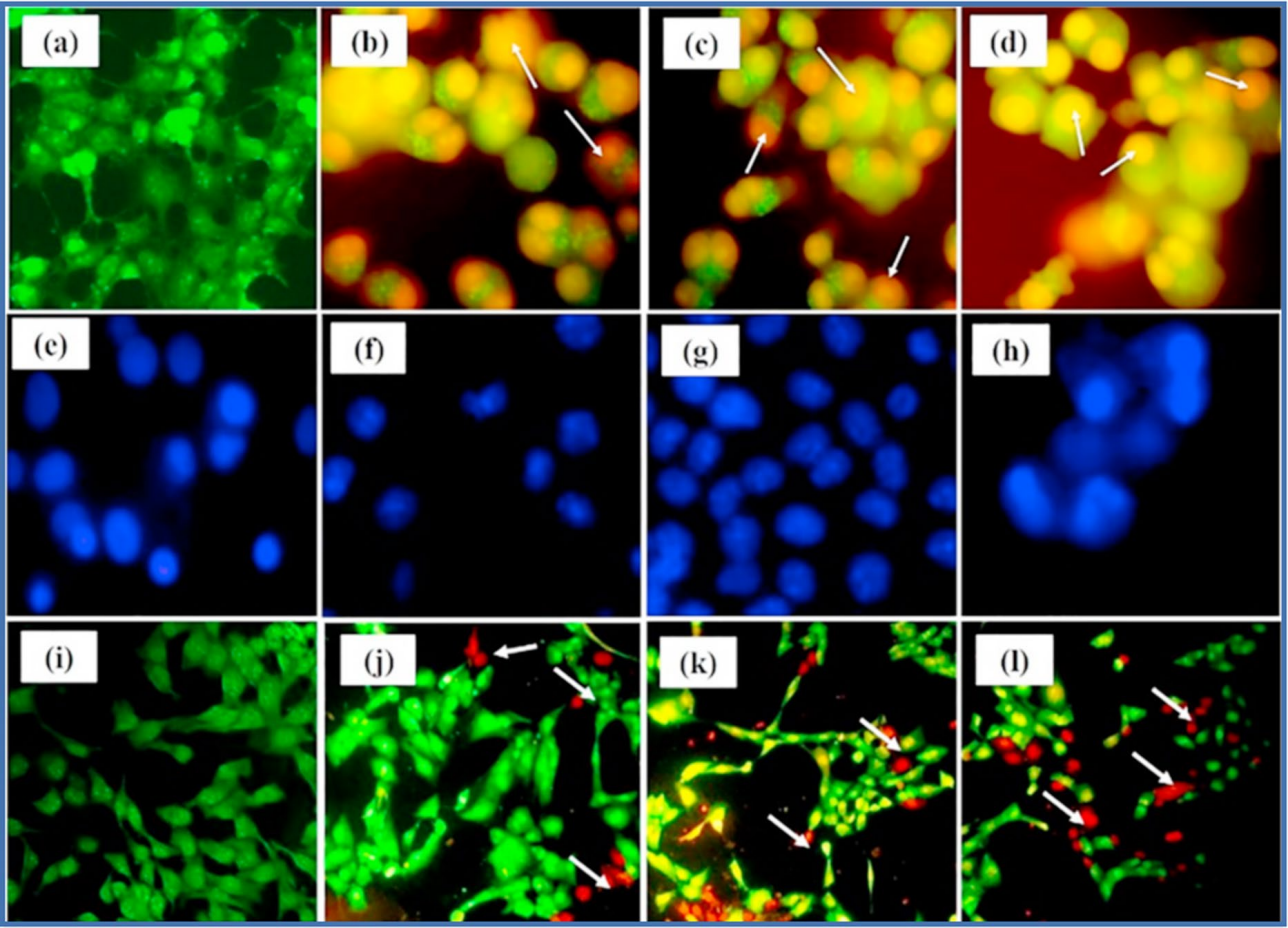
Fluorescence microscopy images illustrating the effects of CdS QDs on MCF-7 cell line. **a** Representative image of untreated MCF-7 cells. Images of MCF-7 cells treated with different concentrations of CdS QDs: **b** 15 μg/mL, **c** 30 μg/mL, and **d** 45 μg/mL. The cells were stained with AO/EtBr, where live cells appeared green, early and late apoptotic cells appeared yellow and reddish orange, respectively. Additional images of MCF-7 cells stained with DAPI: **e** untreated cells, **f** 15 μg/mL, **g** 30 μg/mL, and **h** 45 μg/mL of CdS QDs treated cells after 24 h. Apoptotic analysis using Annexin V/FITC staining: **i** untreated MCF-7 cells and CdS QDs treated MCF-7 cells with different concentrations: **j** 15 μg/mL, **k** 30 μg/mL, and **l** 45 μg/mL. Apoptotic cells are indicated by red color spots (arrow). These findings provide insights into the apoptotic effects of CdS QDs on MCF-7 cell line, as observed through fluorescence microscopy and Annexin V/FITC staining. Reprinted with permission from Ref. [294]. Copyright 2019 Elsevier

Metal sulfide quantum dots (QDs) are nanoscale semiconductor particles composed of metal and sulfur atoms, and their exceptional optical and electrical properties make them a compelling choice for a wide range of applications including sensing, imaging, solar cells, and bioimaging in the fields of medicine and biotechnology [292]. A study reported about the *Rhaphanus sativus* L. hairy root regeneration for the biosynthesis of CdS quantum dots in a single step. For the biosynthesis of CdS quantum dots (QDs), its extracts were used as the organic source for reducing and stabilizing cadmium (Cd) and sulfur (S) precursor ions. Further, the results of the MTT assay revealed a dose-dependent decrease in cell viability upon treatment with a CdS QDs solution, compared to cells treated with a blank plant extract serving as a control. The reduction in cell viability was more pronounced with higher concentrations of CdS QDs in both cell lines, with MCF-7 cells exhibiting a more significant response. The viability of MCF-7 cells was markedly reduced at a concentration of 12.5 mg/mL, showing the highest effectiveness with 20% inhibition at the maximum tested concentration of CdS QDs [293]. Shivaji et al. (2019) employed waste-matured tea leaves (*Camellia sinensis*) as bio-surfactants for the green synthesis of CdS QDs. An MTT experiment assessed the MT-CdS cytotoxicity against MCF-7 cells. Figure 14a–d illustrates orange AO/EB staining, displaying apoptotic characteristics such as cell shrinkage, nuclear condensation, and fragmented nuclei. Increased concentrations of MT-CdS QDs resulted in enhanced apoptosis. Figure 14e shows untreated MCF-7 cells with spherical nuclei and weak fluorescence, while MT-CdS QDs-treated cells exhibited high fluorescence (Fig. 14f–h), indicating apoptotic nuclei and nuclear condensation. Morphological alterations in MT-CdS QDs-treated MCF-7 cells were observed using a bright field inverted microscope. Annexin V-FITC staining of untreated MCF-7 cells (Fig. 14i) displayed green emission, indicating cell viability. However, MCF-7 cells treated with different doses of MT-CdS QDs exhibited red emission in fluorescence images (Fig. 14j–l), indicating apoptosis. Moreover, MT-CdS QDs resulted in increased red spot density, indicating effective cell killing [294].

### Bimetallic nanoparticles

Bimetallic nanoparticles, composed of two different metals, offer unique properties and synergistic effects that make them highly attractive for a variety of applications, including catalysis, sensing, energy conversion, and biomedical fields [295]. Another study focused on the efficient and environmentally friendly synthesis of silver (Ag), gold (Au) NPs, and bimetallic Au-AgNPs composites using a bio-waste extract derived from *Trapa natans*. In this study, the cytotoxicity of the synthesized Ag-Au composite NPs was evaluated by conducting cell viability assays on different cancer cell lines treated with varying concentrations (25–400 μg/mL) for 48 h. The exposure of this NPs composite resulted in reduced cell viability in HeLa (cervical) and MDA-MB-231 (breast) cell lines [296]. Adeyemi et al. (2019) conducted a study focusing on the synthesis of monometallic NPs using a Kei apple (*Dovyalis caffra*) fruit extract, specifically Ag and Au-NPs, as well as their bimetallic counterparts, Au–Ag nanoparticles (BNPs). The results obtained from the study revealed that among the synthesized NPs, AuNPs exhibited the lowest anticancer potency, while Au–Ag BNPs2 demonstrated the highest potency. This suggests that the combination of gold and silver in the bimetallic NPs improved anticancer activity, particularly for Au–Ag BNP2 and Au–Ag BNP3. Interestingly, the activity of Au–Ag BNPs2 was 2.6 times higher than that of the individual monometallic NPs. These findings unveiled the synergistic effects of the individual metals that contribute to the enhanced anticancer properties of the bimetallic NPs [297].

A study reported a green synthesis approach for producing palladium-silver nanoparticles (Pd–Ag NPs) using a *Nigella sativa* seed extract, where their inhibitory effects were assessed on different cancer cell lines, namely human BC cells, human endometrial carcinoma cells, and human cervical cancer cells. Upon evaluation, IC_50_ values for Pd–Ag NPs against the aforementioned cancer cell lines were determined to be 12.4384 ± 0.39 µg/ml, 13.5043 ± 0.539 µg/mL, and 17.7172 ± 0.782 µg/ml, respectively [298]. Zadehet al. (2022) reported bimetallic nanostructures composed of Cu/Zn, which were synthesized via a biogenic approach using *Lonicera caprifolium* plant extracts. This investigation focused on assessing the cytotoxic activity of copper/zinc bimetallic NPs against the MCF-7 cell line. Upon evaluation, the IC_50_ was determined to be approximately 54 µg/mL. Remarkably, these NPs exhibited notable toxicity towards MCF-7 cells, with cytotoxic effects reaching 100% when administered at concentrations exceeding 600 µg/mL [299]. The enlist of the plant species and their extracts for synthesizing metal and metal oxide NPs and their effectiveness against various BC cell lines have been comprehended in Table 1.

**Table 1:** Plant species and their extracts for synthesizing metal and metal oxide nanoparticles and their effectiveness against different breast cancer cell lines

Plant name	Plant part	Nanoparticle	Size (nm)	Shape	Breast cancer cell line	References
*Acalypha indica Linn*	Leaf	Au/Ag-NPs	20–30	Spherical	MDA-MB-231	[251]
*Commiphora wightii*	Leaf	Au-NPs	20	Spherical/triangular	MCF-7	[252]
*Punica granatum*	Plant	Au-NPs	70–80	Spherical	MCF-7	[253]
*Foeniculum Vulgare*	Seed	Au-NPs	17–20	Spherical	HCC1187, T47D, ZR-75–30	[254]
*Ziziphus spina-christi*	Leaf	Au-NPs	540	Spherical	MCF-7	[255]
*Spondias dulcis*	Peel	Au-NPs	11–36	Spherical	MCF-7	[256]
*Aloe vera*/*Gymnema sylvestre*	Leaf	Au-NPs	20–50	Spherical	MCF-7	[257]
*Podophyllum. hexandrum*	Plant	Au/Ag-NPs	20–80	Spherical	MCF-7, MDA-MB-231	[259]
*Calendula officinalis*	Leaf	Sn-NPs	16–60	Variable shape	MCF7, Hs 319.T, MCF10	[260]
*Syzygium aromaticum*	Plant	Ag-NPs	5–50	Spherical	MCF-7, A549	[261]
*Ziziphus mauritiana*	Fruit	Ag-NPs	428	Spherical	MCF-7 breast	[262]
*Cucumis prophetarum*	Leaf	Ag-NPs	30–50	Spherical	MDA-MB-231, HepG2, A459, MCF-7	[215]
*Tamarindus indica*	Fruit	Ag-NPs	20–25	Spherical	MCF-7	[263]
*Fagonia* cretica	Plant	Ag-NPs	50–80	Spherical	MCF-7	[264]
*Brassica oleracea*	Leaves	Ag-NPs	20	Spherical	MCF-7	[265]
*Rubus* fairholmianus	Root	Ag-NPs	30–150	Spherical/roads	MCF-7	[266]
*Berberis vulgaris*	Plant	Ag-NPs	5–20	Spherical/triangular	MCF-7, A549, Hep-2	[269]
*Viburnum nervosum*	Leaf	Ag-NPs	445	Spherical	MCF-7, A431	[268]
*Xanthoria* parietina	Plant	Ag-NPs	10–40	Spherical	MDA-MB-231, HCT116	[267]
*Flavopunctelia* flaventior	Plant	Ag-NPs	10–40	Spherical	MDA-MB-231, HCT116	[267]
*Cystoseira myrica*	Plant	Ag-NPs	12–24	Spherical	MCF-7	[182]
*Gracilaria foliifera*	Plant	Ag-NPs	12–24	Spherical	MCF-7	[182]
*Ulva rigida*	Plant	Ag-NPs	12–24	Spherical	MCF-7	[182]
*Ceropegia bulbosa Roxb*	Tuber	Se-NPs	55	Spherical	MDA-MB-231	[270]
*Punica granatum*	Fruit	CuO-NPs	32	Spherical	Caco-2, HepG-2, MDA-MB-231	[273]
*Annona muricata*	Plant	CuO-NPs	6–32	Spherical	MCF-7, HBL-100, AMJ-13	[274]
*Olea europaea*	Leaf	CuO-NPs	50–100	Spherical	AMJ-13, SKOV-3	[275]
*Eucalyptus globulus*	Leaf	CuO-NPs	20–50	Spherical	MCF-7	[276]
*Helianthus* tuberosus	Plant	CuO-NPs	148	Spherical	MDA-MB-231	[277]
*Wrightia tinctoria*	Plant	Cu-NPs	15–40	Spherical	MCF-7	[279]
*Manilkara* zapota	Leaf	Cu-NPs	18	Spherical	MCF-7	[278]
*Celosia argentea*	Leaf	Iron oxide	10–40	Spherical	MCF-7	[280]
*Aloe vera*	Leaf; seed	Iron oxide	50; 20–40	Spherical	MCF-7	[281]
*Linum usitatissimum*	Leaf; seed	Iron oxide	50; 20–40	Spherical	MCF-7	[281]
*Ocimum basilicum*	Leaf	Fe_3_O_4_-NPs	15	Spherical	MCF-7	[282]
*Albizia adianthifolia*	Leaf	Fe_3_O_4_-NPs	32–100	Spherical	AMJ-13, MCF-7	[283]
*Origanum majorana*	Leaf	ZnO-NPs	32	Spherical	MCF-7, HT-29	[287]
*Cucurbita pepo*	Seed	ZnO-NPs	50–60	-	MDA-MB-231	[286]
*Pandanus* odorifer	Leaf	ZnO-NPs	90	Spherical	MCF-7, HepG2, A-549	[284]
*Hertia intermedia*	Flower	ZnO-NPs	20–80	Spherical	MDA-MB-231 HEK-293	[285]
*Alstonia macrophylla*	Leaf	ZnO-NPs	50–100	Spherical	MCF-7, HepG2, A-549	[288]
*Vitis vinifera*	Grape	NiO-NPs	25	Spherical	MCF-7	[300]
*Andrographis Paniculata*	Leaf	NiO-NPs	24	Spherical	MCF-7	[301]
*Nigella sativa*	Seed	Pt-NPs	1–6	Spherical	MDA-MB-231, HeLa	[271]
*Justicia gendarussa*	Leaf	TiO_2_	40–100	spherical	MCF-7, MDA-MB-231	[289]
*Limonia acidissima*	Peel	TiO_2_	20–35	Spherical	MCF-7	[290]
*Synadenium cupulare*	Plant	CdO/CdCO_3_	200	Spherical	MCF-7, MDA-MB-231	[291]
*Rhaphanus. sativus L*	Root	CdS	2–7	Spherical	MCF-7	[293]
*Camellia sinensis*	Waste leaves	CdS	2–10	Spherical	MCF-7	[294]
*Trapa natans*	Plant	Ag–Ag	40–100	Spherical	MDA-MB-231, HCT116, HeLa	[296]
*Dovyalis caffra*	Plant	Au–Ag	40 60	Spherical	MCF-7	[297]
*Nigella sativa*	Seeds	Pd–Ag	6–10	Spherical	MDA-MB-231, HeLa	[298]
*Lonicera caprifolium*	Plant	Cu–Zn	30–60	-	MCF-7	[299]

## Hurdles and challenges associated with biogenic nanomaterials

Biogenic metal and metal oxide nanoparticles (NPs) hold immense promise for diverse industrial applications, owing to their unique properties and environmentally sustainable synthesis routes. However, the widespread implementation of these nanoparticles requires a thorough understanding and resolution of certain limitations. This review explores the significant factors influencing the size and shape variations of biogenic metallic NPs, highlighting key challenges and proposing strategies to address them. By addressing these limitations, the industrial utilization of biogenic metal and metal oxide NPs can be advanced, paving the way for innovative and sustainable technologies [204]. Additionally, the location of NPs and alterations in the tissue metal ion composition also impact their movement and penetration, resulting in additional nucleation events and increased metal deposition [302]. The diverse makeup of synthesized nanoparticles (NPs) presents obstacles in applications that demand exact dimensions and configurations. This diversity in composition can complicate efforts to precisely control the sizes and shapes of nanoparticles, which are crucial for many applications such as tissue engineering, biosensing, wound healing, drug delivery, breast cancer therapy.

Extraction, isolation, and purification of nanoparticles (NPs) indeed pose significant challenges due to the complexity of biological matrices and the delicate nature of these particles. Biogenic NPs, derived from plants and other organisms, often face additional hurdles due to the presence of rigid cell walls and intricate molecular structures. Here's a more detailed elaboration on the techniques and challenges involved. *Centrifugation*: Centrifugation is a common technique used for separating NPs from biological samples. However, it may not be efficient for all types of NPs, especially those with similar densities to surrounding biomaterials. Additionally, centrifugation can sometimes cause aggregation of NPs, reducing their recovery rates.

*Organic solvents*: Organic solvents are frequently employed for NP extraction and stabilization. While effective in some cases, they can also induce aggregation and may not be suitable for all types of NPs. Moreover, the use of organic solvents raises concerns about potential toxicity and environmental impact. *Surfactants*: Surfactants are used to stabilize NPs and prevent aggregation. However, excessive use of surfactants can lead to unwanted interactions with biological molecules and affect the properties of NPs. *Physicochemical techniques*: Various physicochemical techniques, such as heating, freeze-thawing, and osmotic shock, are employed to disrupt biological structures and facilitate NP extraction. However, these techniques can also cause issues such as aggregation, precipitation, and alterations in NP size and shape. *Cell wall disruption*: The rigid cell walls of plants and other organisms present a significant barrier to NP extraction. Disrupting these cell walls without damaging the NPs themselves is a critical challenge in the purification process. *Selective extraction*: Selective extraction of NPs from complex biological matrices remains a major challenge. Different NPs may require specific extraction methods tailored to their unique properties and interactions with biomolecules.

Despite the potential of various NPs in next-generation theranostic medications, safety concerns persist in clinical studies, particularly regarding biogenic NPs as properties like size, shape, surface charge, and chemistry can influence their pharmacokinetics, pharmacodynamics, and cytotoxicity of these NPs [303]. Nonetheless, advances in the manufacturing and biocompatibility of green-synthesized nanomaterials offer promising avenues for further investigation. However, several issues need to be addressed before the clinical application of these materials.

## Conclusions and future prospects

In conclusion, breast cancer is a complex disease with a high global incidence and mortality rate. The plant-mediated synthesis of biogenic NPs offers a sustainable and environmentally friendly alternative to conventional chemical procedures. Plant extracts rich in bioactive compounds have shown significant anticancer properties against breast cancer, inhibiting cell proliferation, inducing apoptosis, suppressing angiogenesis, and reducing inflammation. Biogenic NPs synthesized using various plant extracts have demonstrated cytotoxic effects on breast cancer cell lines while exhibiting minimal toxicity towards normal cells. These NPs induce apoptosis, alter cell cycle distribution, and modulate p53-mediated DNA damage response. They also offer advantages such as biocompatibility, targeted drug delivery, and reduced harm to healthy cells. However, further research is needed to understand the mechanisms of action, optimize formulation and dosage, and evaluate safety and efficacy in clinical trials. Using plant extracts for biogenic nanoparticle synthesis holds great promise for effective breast cancer therapies.

Future prospects in this field include synthesizing biogenic NPs using plant extracts rich in bioactive compounds, standardizing synthesis methods, optimizing nanoparticle stability, and evaluating underlying mechanisms of antimicrobial activity. It is important to assess the cytotoxic effects of these NPs on breast cancer cell lines while minimizing toxicity to normal cells. Elucidating the mechanisms of action, performing molecular docking analyses, optimizing formulation and dosage, and evaluating biocompatibility and targeted drug delivery capabilities through in vivo studies are also crucial. Additionally, more clinical trials are needed to support the development of highly effective medication delivery systems. Furthermore, additional research should aim to identify key molecules involved in the biogenic nanoparticle synthesis and function of biogenic NPs and address unresolved fundamental questions in this field. Advancements in biogenic nano-medicine have the potential to improve breast cancer therapy and patient outcomes significantly.

## Data Availability

No datasets were generated or analysed during the current study.

## Abbreviations

BC: Breast Cancer
MDR: Multi-drug resistance
DCIS: Ductal carcinoma in situ
LCIS: Lobular cancer in situ
ILC: Invasive lobular carcinoma
TNBC: Triple-negative breast cancer
ER: Estrogen receptor
PR: Progesterone receptor
HER2: Human epidermal growth factor receptor 2
DNA: Deoxyribonucleic acid
BUSI: Breast ultrasound image dataset
ML: Multiple machine learning
MRI: Magnetic Resonance Imaging
SERS: Surface-Enhanced Raman Spectroscopy
EPR: Enhanced Permeability and Retention
DOX: Doxorubicin
HR-TEM: High-resolution transmission electron microscopy
SEM: Scanning electron microscopy
EDX: Energy-dispersive X-ray spectroscopy
XRD: X-ray diffraction
XPS: X-ray photoelectron spectroscopy
BET: Brunauer–Emmett–Teller
Ag NPs: Silver nanoparticles
Au NPs: Gold nanoparticles
MO: Metal oxide (M = Ni, Cu, Zn, Fe, Si, Co, Mg etc.)
CFO: Cobalt spinel ferrite NPs
QD: Quantum dot
